# Identifying Structural Factors Governing the Photodynamic Activity of Phthalocyanines

**DOI:** 10.1021/acs.jmedchem.5c03090

**Published:** 2026-02-06

**Authors:** Magdalena Kozlikova, Mary Angelia Alfred, Miloslav Machacek, Fabienne Dumoulin, Andrés de la Escosura, Tomasz Goslinski, Marie Halaskova, Jian-Dong Huang, Mei-Rong Ke, Saad Makhseed, Dariusz T. Mlynarczyk, Dennis K. P. Ng, Tomás Torres, Roy C. H. Wong, Petr Zimcik, Veronika Novakova

**Affiliations:** † Faculty of Pharmacy in Hradec Kralove, Charles University, Ak. Heyrovskeho 1203, Hradec Kralove 500 03, Czech Republic; ∇ Faculty of Engineering and Natural Sciences, Department of Biomedical Engineering and Department of Chemistry, Acibadem Mehmet Ali Aydınlar University, Ataşehir, Istanbul 34752, Türkiye; § Acibadem Mehmet Ali Aydinlar University, Graduate School of Natural and Applied Sciences, Ataşehir, Istanbul 34752, Türkiye; ∥ Department of Organic Chemistry and Institute for Advanced Research in Chemistry (IAdChem), 16722 Universidad Autónoma de Madrid, C/Francisco Tomás y Valiente 7, 28049 Madrid, Spain; ⊥ Chair and Department of Chemical Technology of Drugs, Poznan University of Medical Sciences, Rokietnicka 3, 60-806 Poznań, Poland; # College of Chemistry, Fujian Provincial Key Laboratory of Cancer Metastasis Chemoprevention and Chemotherapy, 12423 Fuzhou University, Fuzhou 350116, China; ○ Department of Chemistry, 37603 Kuwait University, PO Box 5969, Safat 13060, Kuwait; ☆ Department of Chemistry, The Chinese University of Hong Kong, Shatin, New Territories, Hong Kong 999077, China; ◆ IMDEA-Nanociencia, Campus de Cantoblanco, 28049 Madrid, Spain

## Abstract

Phthalocyanines (Pcs) are promising photosensitizers (PSs) for photodynamic therapy (PDT). However, the variability in experimental conditions in *in vitro* experiments among reported derivatives complicates clear comparisons. In this study, we systematically evaluated a diverse set of more than 40 cationic, anionic, nonionic Zn, Mg, or metal-free or axially substituted silicon Pcs and compared them under standardized conditions. Their spectral and photophysical properties, interactions with bovine serum albumin, subcellular localization, and *in vitro* PDT efficacy in three human cancer cell lines were assessed. Structural features influencing PDT efficacy include their presence in the monomeric state through axial substitution (in silicon Pcs) or rigid bulky peripheral groups with the latter enhancing activity in cationic derivatives while reducing it in nonionic derivatives. Amphiphilic structures significantly improved the PDT efficacy, especially for nonionic and anionic Pcs. The results of this study provide clear design principles for the future development of highly efficient PSs for PDT.

## Graphical abstract

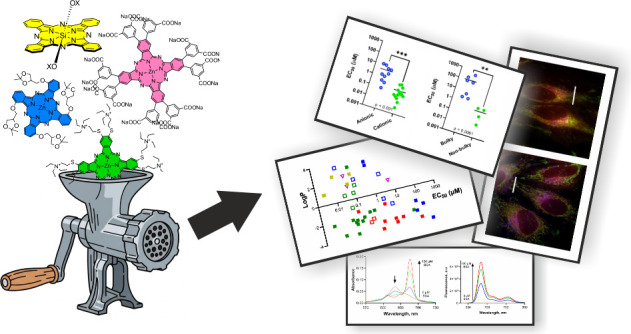

## Introduction

Photodynamic therapy (PDT) has emerged as a promising minimally invasive treatment modality for cancer. Its principle is based on the accumulation of a photosensitizer (PS) in cancerous tissues, followed by its activation with light of a specific wavelength. This activation leads to the generation of reactive oxygen species, primarily singlet oxygen (^1^O_2_), which induce cytotoxic effects and ultimately lead to cancer cell death. Many structural types of PSs have been shown to have photodynamic properties, and mainly the representatives of porphyrins and phthalocyanines (Pcs) have been used in clinical cancer treatment.^1^ This is probably due to their unique spectral and photophysical properties (i.e., high singlet oxygen production), good photostability, and the possibility of fine-tuning their properties (e.g., water solubility, aggregation suppression, and position of absorption maxima). Compared with porphyrins, Pcs have the advantage of strong absorption in the far-red and near-infrared regions of the visible spectrum, enabling deeper penetration of activating light through the tissues. This is why interest in these macrocycles is growing exponentially.^2,3^

As of June 2025, more than 5,300 publications related to the use of Pcs in PDT have been indexed in the Web of Science (search terms: *phthalocyanine*, *photodynamic*). Of these, approximately 1,500 reports include *in vitro* investigations (additional search term: *in vitro*). Collectively, this body of literature constitutes a substantial resource that, in principle, could enable the identification of key structural determinants and the rational design of optimized therapeutic candidates. However, deriving such conclusions remains challenging because of the considerable heterogeneity in the experimental conditions used across *in vitro* studies, including the type of light source (LEDs, lasers, or broad-spectrum lamps), irradiation intensity, incubation time of Pcs with cells, drug-to-light interval, irradiation duration, and choice of cell lines. Consequently, reported values of the half-maximum effective concentration (EC_50_) are often not directly comparable across studies.

To address the limitations associated with cross-study comparisons, more than 40 representative Pcs with a variety of substitutions (Figure 1), which have already been reported to show favorable photodynamic properties, were analyzed. Their photophysical characteristics and photodynamic efficacy *in vitro* on three cell lines were systematically evaluated under standardized experimental conditions. Through this comprehensive comparative experimental study, we aimed to elucidate the basic structure–activity relationships underlying the efficacy of Pcs in PDT, ultimately leading to structural recommendations for the development of more effective PSs for cancer treatment.

**Figure 1:**
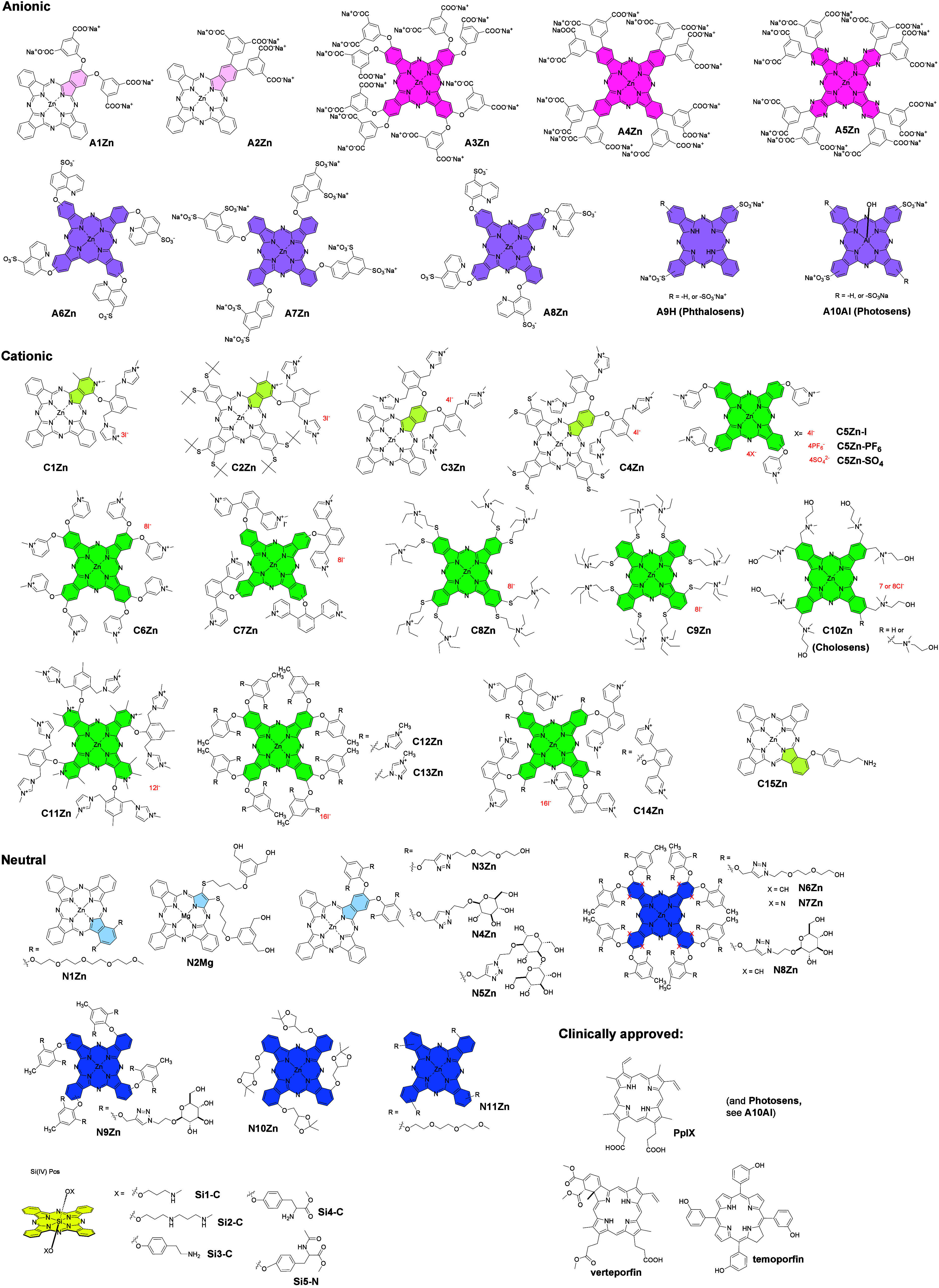
Structures of the compounds involved in the study. Color code always indicates the isoindole unit that is modified with the hydrophilic substituent. Light version of the color stresses the unsymmetrical composition of the substitution. Pink – anionic Pcs with carboxylic function, magenta – anionic Pcs with sulfonate functions, green – cationic Pcs, blue – nonionic Pcs, yellow – silicon Pcs, irrespective of the axial substitution. Note: **C10Zn** (known as Cholosens) is a mixture of Zn­(II)­Pcs having either seven or eight *N*-(2-hydroxyethyl)-*N*,*N*-dimethylammoniomethyl groups with an average substitution degree of 7.5.

## Results and Discussion

### Compounds Involved in the Study

The compounds investigated in this study were provided by research groups worldwide with expertise in PDT. The set includes a diverse array of structurally distinct Pcs, which demonstrated notable activity, as reported in the original publications. In this study, we did not consider the use of any drug delivery system, carriers or conjugation with targeting moieties or the formation of nanoscale structures (which may change solubility, aggregation, targeting, etc.)^4–7^ but focused only on compounds that are directly applied to cells. Several factors were considered for the selection of compounds in this study (Figure 1).

Hydrophilicity is a crucial property since a biological application is intended. Thus, compounds are decorated with charged (i.e., anionic (**A-series**), cationic (**C-series**)) or neutral polar (**N-series**) substituents to achieve sufficient water solubility. Among anionic derivatives, compounds carry either COO^–^ (**A1-A5Zn**) or SO_3_^–^ (**A6-A8Zn**, **A9H**, and **A10Al**) functions. Quaternized nitrogen is present in all of the compounds of the cationic series (**C1-C14Zn**), except **C15Zn**, which contains aliphatic NH_2_ groups. This group is believed to be protonated under physiological conditions.^8^ Therefore, it has been classified as a cationic derivative as well. The neutral polar substituents come from either short PEG chains (**N1Zn**, **N3Zn**, **N6Zn**, **N7Zn**, and **N11Zn**), aliphatic alcohols (**N2Mg**), or carbohydrate moieties (**N4Zn**, **N5Zn**, **N8Zn**, and **N9Zn**). Additionally, **N10Zn** with ketal-protected glycerol moieties (potentially producing diols after acid-induced cleavage in lysosomes) has also been classified as neutral polar.

Compounds in all series also differ in the number and nature of polar groups and, consequently, in log *P* values. Finally, symmetrical hydrophilic derivatives (**A3-A8Zn**, **C5-C14Zn**, and **N6-N11Zn**) as well as unsymmetrical derivatives (**A1-A2Zn**, **C1-C4Zn**, **C15Zn**, **N1Zn**, and **N2Mg**) that are often amphiphilic were included in the study. Amphiphilic character usually favors effective interactions with biomembranes, which can result in changes in the PDT effect.^9,10^ On the other hand, hydrophilic symmetric derivatives may benefit from better monomerization and water solubility. Direct comparison of unsymmetrical and symmetrical hydrophilic derivatives can therefore be very useful.

Aza analogs in which benzene rings are replaced for pyrazines (i.e., tetrapyrazinoporphyrazines) in **A5Zn** and **N7Zn**, as well as for pyridines (i.e., tetra­(3,4-pyrido)­porphyrazines) in **C11Zn**, were included to determine the effect of the type of macrocyclic core.

The central metal plays a significant role in the production of singlet oxygen on the basis of the heavy atom effect.^11–14^ Most of the derivatives in the study have a zinc­(II) center, which is obvious from the code of the respective compounds (e.g., **A1Zn**, **C1Zn**, etc.). There are several exceptions to compounds having magnesium­(II) (**N2Mg**), aluminum­(III) (**A10Al**). and a metal-free form (**A9H**). Other central metals were not investigated in this study, but a recent study also proved that PtPc was a very efficient PS.^15^

Silicon­(IV) Pcs constitute a distinct subgroup of compounds (**Si series**), because of the semimetal character of silicon and the important structural features this atom provides. Unlike other complexes, SiPcs allow for axial modification, which can reduce aggregation. The compounds of this subgroup are labeled as **SiX-C** or **SiX-N** in which the letter X denotes the compound number and C or N indicates the presence of a cationic or neutral axial ligand, respectively.

Clinically approved PSs^16^ such as **protoporhyrin IX** (**PpIX**, the metabolic product of the 5-aminolevulinic acid (ALA) prodrug), **verteporfin**, **temoporfin**, and **Photosens** (i.e., **A10Al**) were used for comparison.

The synthetic procedures and details of the characterization of all the compounds involved in the study can be found in the original publications in the references listed in Table 1. In the following text, the physicochemical and photophysical properties are first described, followed by an evaluation of the photodynamic activity *in vitro* with attempts to correlate the efficacy with several structural parameters. All the experiments, if not otherwise stated, were performed under the same conditions in the same laboratory to avoid variability in the experimental conditions.

**Table 1:** Photophysical Data of Investigated Derivatives in DMF^a^

Cpd.	λ_A_/nm	ε/M^–1^cm^–1^	λ_F_/nm	Φ_F_	τ_F1_/ns	τ_F2_/ns	Φ_Δ_	Log *P*	Synthesis in ref
**A1Zn**	673	165 600	682	0.035	0.44 (15%)	2.51 (85%)	0.26	–2.70	^17^
	681	164 000							
**A2Zn**	677	152 090	688	0.02	0.23 (16%)	2.66 (84%)	0.22	–2.42	^17^
**A3Zn**	677	172 640	688	0.03 (0.19)^b^	0.82 (18%)	2.57 (82%)	0.24 (0.42)^b^	–2.88	^17^
**A4Zn**	694	168 630	708	0.01 (0.12)^b^	0.36 (4%)	2.78 (96%)	0.45 (0.45)^b^	–3.32	^17^
**A5Zn**	659	138 740	675	0.03	0.20 (56%)	1.11 (44%)	0.21	–3.27	^17^
**A6Zn**	681	122 420	692	0.28	3.14		0.58	–2.89	^18^
**A7Zn**	696	109 950	707	0.23	2.57		0.55	–2.92	^19^
**A8Zn**	696	197 610	708	0.26	2.83		0.69	–3.64	^18^
**A9H** ^c^	694, 662	104 650	703	0.22	1.97 (18%)	3.87 (82%)	0.19	–1.35	^20^
**A10Al** ^d^	711, 688	105 250	717	0.62	4.14 (3%)	5.60 (97%)	0.25	–1.20	^20^
**C1Zn**	689	100 240	696	0.15	1.88		0.66	0.53	^17^
**C2Zn**	708	177 880	718	0.30	1.67		0.70	1.38	^10^
**C3Zn**	673	177 190	682	0.19	2.47		0.53	–0.84	^17^
**C4Zn**	697	106 360	709	0.21	1.64		0.44	0.62	^10^
**C5Zn-I**	674	130 860	682	0.25	2.38 (97%)	6.53 (3%)	0.49	–2.19	^21^
**C5Zn-PF**_**6**_	674	86 800	682	0.28	2.32 (97%)	6.31 (3%)	0.45	–2.37	^22^
**C5Zn-SO**_**4**_	674	82 140	682	0.08	2.47 (94%)	4.93 (6%)	0.28	–3.57	^21^
**C6Zn**	681	41 730	683	0.001	0.07 (3%)	2.84 (97%)	0.02	–2.59	^23^
**C7Zn**	684	121 730	693	0.23	2.07 (95%)	4.86 (5%)	0.37	–2.47	^24^
**C8Zn**	704	281 600	714	0.29	1.52 (14%)	2.41 (86%)	0.63	–1.59	^25^
**C9Zn**	757	134 170	783	0.06	0.62 (8%)	1.14 (92%)	0.64	–1.65	^25^
**C10Zn**	686	40 580	695	0.12	1.56 (25%)	2.37 (75%)	0.41	–2.03	^20^
**C11Zn**	716	160 750	726	0.21	1.41		0.53	–2.25	^17^
**C12Zn**	679	244 030	687	0.20	0.62 (7%)	2.23 (93%)	0.48	–2.08	^26^
**C13Zn**	679	236 490	687	0.20	0.60 (4%)	2.27 (96%)	0.37	–3.47	^27^
**C14Zn**	690	222 730	698	0.22	0.79 (34%)	2.04 (66%)	0.40	–2.38	^24^
**C15Zn**	675	136 590	684	0.20	1.23 (7%)	2.96 (93%)	0.53	3.32	^8^
**N1Zn**	689	82 550	702	0.24	2.41		0.67	3.52	^28^
**N2Mg**	684	15 950	687	0.16	1.29 (25%)	3.39 (75%)	0.17	1.97	^29^
**N3Zn**	672	280 340	681	0.28	3.43		0.48	1.90	^30^
**N4Zn**	672	293 750	681	0.29	3.52		0.59	–0.65	^30^
**N5Zn**	672	209 500	681	0.28	3.44		0.47	–1.33	^30^
**N6Zn**	681	237 520	690	0.26	1.04 (3%)	3.17 (97%)	0.46	–2.59	^31^
**N7Zn**	629	195 490	641	0.18	0.74 (5%)	2.45 (95%)	0.58	–1.60	^31^
**N8Zn**	680	275 660	689	0.25	3.30		0.45	–3.64	^32^
**N9Zn**	700	248 160	712	0.24	2.63		0.57	–2.02	^32^
**N10Zn**	697	147 000	708	0.23	2.34		0.68	3.48	^33^
**N11Zn**	699	146 930	710	0.21	2.43		0.53	3.10	^34^
**Si1-C**	676	214 560	683	0.15	1.00 (50%)	4.50 (50%)	0.20	2.44	^35^
**Si2-C**	671	229 280	679	0.25	1.00 (9%)	5.15 (91%)	0.23	1.64	^35^
**Si3-C**	680	204 080	687	0.06	0.55 (65%)	4.63 (35%)	0.05	2.57	^8^
**Si4-C**	680	236 170	689	0.07	0.96 (96%)	2.28 (4%)	0.06	2.69	^36^
**Si5-N**	680	132 950	689	0.10	1.23 (69%)	1.68 (31%)	0.085	1.05	^36^
**PpIX**	630		634	0.12	1.79 (20%)	13.8 (80%)	0.73^e^	1.06	commercial
**verteporfin**	690		696	0.18	6.05		0.75^e^	2.51	commercial
**temoporfin**	651		656	0.24	9.66		0.76^e^	1.40	commercial

a Absorption maximum in the Q-band (λ_A_); extinction coefficient (ε); fluorescence emission maximum (λ_F_); fluorescence quantum yield (Φ_F_) determined by the comparative method using unsubstituted zinc phthalocyanine as reference (Φ_F_(ZnPc) = 0.32 in THF); fluorescence lifetime (τ_F_); quantum yield of singlet oxygen production (Φ_Δ_) determined by the comparative method using unsubstituted zinc phthalocyanine (Φ_Δ_(ZnPc) = 0.56 in DMF) for all studied Pcs or Rose Bengal (RB) (Φ_Δ_(RB) = 0.47 in DMF for PpIX, verteporfin, and temoporfin) as reference and DPBF as a scavenger; experimentally determined partition coefficient between 1-octanol and PBS in logarithmic scale (log *P*).

b Determined for their nonionized analogues containing free carboxylic acid; value taken from ref 17.

c Phthalosense.

d Photosens.

e With RB as reference.

### Spectral Properties

Since the effect of PDT is based on the activation of the PS by light, spectral properties represent important parameters that may respond to the environment. Detailed spectral studies were therefore carried out in DMF, where the vast majority of the compounds are monomeric, as well as in aqueous media (phosphate-buffered saline (PBS) and water), where the compounds can be partially or fully aggregated. Aggregation is undesirable in PDT because it leads to energy dissipation predominantly through internal conversion, thereby reducing or even completely inhibiting singlet oxygen production. Whereas a steep narrow Q-band is typical for monomeric species, aggregation is normally manifested by the broadening of the Q-band and the appearance of a new blueshifted band corresponding to H-aggregates (see spectra of representative examples in Figure 2 and the Supporting Information for all compounds).

**Figure 2:**
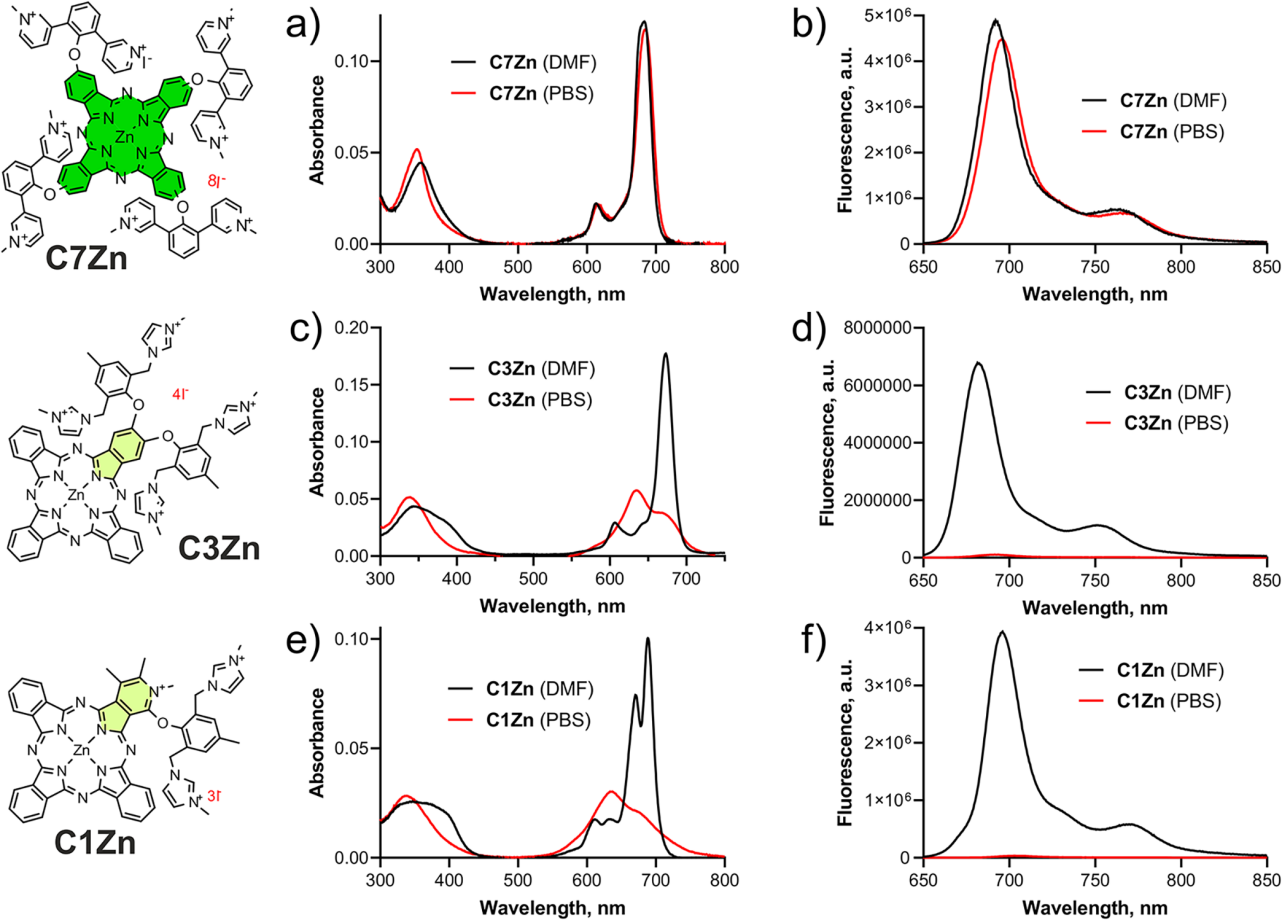
Representative examples (*c* = 1 μM) of absorption (a, c, e) and fluorescence emission spectra (b, d, f) of compound **C7Zn**, which is monomeric in both DMF and aqueous medium (e.g., PBS) (a, b); **C1Zn** and **C3Zn**, which are monomeric in DMF but are aggregated in PBS (c, d, e, f), and compound **C1Zn** having a split Q-band in monomeric form (e, black line). Spectra of all compounds of the series are shown in Supporting Information Figures S1–S43.

Owing to the Pc core, most compounds of the series exhibit strong absorption with a low-energy Q-band between 660 and 710 nm and extinction coefficients between 150 and 300 000 M^–1^ cm^–1^ in DMF (Table 1). The position and shape of the Q-band are related to the type of core, the position of the substituents, and the central atom. Owing to the C_4_-symmetry of the macrocycle, the Q bands of all the symmetrical compounds of the series in DMF were nonsplit (Figure 2a). In the case of unsymmetrical derivatives, both split and nonsplit Q bands were observed, which were dependent on the characteristics of the substituents. If the substituents do not have a strong electron-donating or electron-withdrawing effect or if they have a comparable effect, then splitting is usually not observed, even if the macrocycle has low symmetry. For example, splitting is significant at **C1Zn** (Figure 2e, black line) but less pronounced at **C2Zn**. Nonsplit Q bands are present in the spectra of **C3Zn**, **C4Zn**, and most of the other low-symmetrical Pcs of the series (Figure 2c, black line). Significant splitting can be observed in the case of metal-free **A9H**, again as a result of the loss of symmetry due to the presence of two central hydrogens instead of a metal ion. Notably, the splitting of **A10Al** in DMF is probably caused by an inconsistent sample composition due to the variable number of SO_3_H groups, and splitting disappeared in aqueous media, where a nonsplit Q-band was present.

In terms of the position of the Q-band, compared to the corresponding Pc analogues **A4Zn** and **N6Zn**, the pyrazine analogs **A5Zn** and **N7Zn** exhibit well-known blueshifts of 52 and 35 nm, respectively.^37^ On the other hand, significant red-shifts can be achieved by shifting the substituents from peripheral (β positions) to nonperipheral positions (α positions),^38^ which is reflected by the difference of 53 nm between the Q bands of **C9Zn** (eight α, 757 nm) and **C8Zn** (eight β, 704 nm). Similarly, a redshift of approximately 20 nm can be seen in **N9-N11Zn** (four α, ∼700 nm) compared with the structurally similar **N6Zn** and **N8Zn** derivatives (both four β, ∼680 nm).

### Hydrophilicity

Because these compounds are intended for biological applications, studying their behavior in an aqueous environment is necessary. The Pc core is inherently highly hydrophobic and prone to π–π stacking, but the hydrophilicity of the whole molecule can be modulated to a variable degree through the appropriate substituents. These modifications significantly influence their water solubility as well as their behavior in biological systems (e.g., membrane permeability, interaction with biomolecules, distribution). To examine the effects of various hydrophilizing groups, the partition coefficients between octanol and PBS (log *P*) were determined for the entire series (Table 1). It is evident that a highly hydrophilic character with log *P* values <−2.0, guaranteeing excellent water solubility, can be achieved by all types of peripheral substituents—anionic (**A1**-**A8Zn**), cationic (**C5**-**C7Zn**, **C10**-**C15Zn**), and neutral (**N6Zn**, **N8-9Zn**). The strongest effect on the hydrophilic properties is, however, through the introduction of anionic groups. It was clear from the comparison of unsymmetrical derivatives: anionic derivatives **A1-A2Zn** with the same number of hydrophilic groups exhibited log *P* values that were significantly lower than those of cationic **C3-C4Zn** with the same number of charged substituents, neutral hydrophilic PEGs (**N3Zn**), or carbohydrate (**N4Zn**) moieties. Interestingly, increasing the number of charges in the molecule is not the key to ensuring the high hydrophilicity of the molecule; already with 4 anionic charges (e.g., **A1-A2Zn**), a log *P* of ∼−2.5 can be achieved, whereas 16 such charges (**A3-A5Zn**) lead to only a slight reduction to a value of log *P* ∼ −3.0. With respect to the Si­(IV)­Pcs series, **Si1-Si5** are lipophilic derivatives that are characterized by log *P* values greater than 0; therefore, the use of stock solutions in DMF or DMSO (instead of water) was required for spectral studies in aqueous media or *in vitro* tests (for details, see the Supporting Information).

### Aggregation

The aggregation of various PSs in PDT is generally an unwanted property, as it decreases the activity of Pcs by the aggregation-caused quenching (ACQ) of the excited states. On the other hand, the aggregation of Pcs can also be beneficial, as it may introduce specific properties to the resulting aggregates leading to emerging applications^39^ such as conversion to efficient type I PSs^40,41^ or PSs for sonodynamic therapy,^42^ photothermal therapy,^43^ photoacoustic imaging,^44^ or even uncommon phenomena, such as an aggregation-enhanced photodynamic effect.^45^ In this work, we focused on the factors leading to a decrease in aggregation and ACQ.

In terms of the degree of aggregation in general, it appears that some of the structural factors promoting monomerization in water are specific to a certain type of compound (A, C, or N series), while others are generally applicable to all types of Pcs. For example, rigid bulky substituents with properly oriented (perpendicular to the macrocyclic ring) polar groups seem to be the universal key tool for all types of Pcs. The polar groups are then forced to be placed above and below the core, thus shielding the lipophilic core from water and maintaining the monomeric state. For the N-series, this is the only possibility evidenced by the fact that only **N6-N8Zn** (8 × β) or **N9Zn** (4 × α) showed a sharp Q-band in water. In the case of anionic derivatives, the presence of 16 charged groups on the periphery (i.e., **A3-A5Zn**) ensured monomerization in aqueous media on the basis of strong repulsive forces. Anionic compounds with 4 or fewer anionic groups showed characteristic features of aggregation. Similar trends were observed in cationic derivatives, where 16 quaternized nitrogens in **C12-C14Zn** also enabled full monomerization. However, the C-series clearly revealed that, if the molecule is properly designed, even fewer charges can be sufficient for full monomerization. The key structural feature proved to be the rigidity of the peripheral arrangement, such that the polar groups cannot bend out and displace from their shielding position. Eight quaternized nitrogens on a flexible aliphatic linker did not sufficiently suppress aggregation (**C6Zn**, **C8Zn**, and **C9Zn**), whereas eight quaternized methylpyridine units incorporated into rigid aryloxy substituents led to complete monomerization (**C7Zn**). Additionally, **C11Zn** achieves full monomerization by combining the rigidity of the arrangement with the introduction of a substituent into sterically more demanding nonperipheral (α-) positions and the introduction of an additional charge into the core itself. Notably, compared with peripheral substituents, all types of Si­(IV)­Pcs (**Si1-Si5**) are advantageous because their axial ligands reduce the level of aggregation more efficiently. However, full monomerization in aqueous solutions was achieved only by those containing axial amino groups (**Si1-Si4**) that are basic and ionized in aqueous solutions (particularly in deionized water, which is more acidic than the physiological pH of 7.4 in PBS).

However, the conclusions from the aggregation must be interpreted very carefully. The biological environment is very complex, and interactions with various components, such as lipids, biomembranes, or proteins, may induce monomerization, as shown below in the example of interactions with bovine serum albumin (BSA). For this reason, the level of aggregation cannot be considered separately.

### Photophysical Properties

The potential of the studied compounds to be good photosensitizers was first studied in solution, where the basic photophysical parameters, i.e., the quantum yield of singlet oxygen production (Φ_Δ_), quantum yield of fluorescence (Φ_F_), and fluorescence lifetime (τ_F_), were determined. Because even partial aggregation would bias the measured values, which could lead to misinterpretation of structure–activity relationships, experiments were performed in DMF, thus ensuring monomerization of the involved Pcs (see above). Comparative methods using unsubstituted zinc­(II) Pc as a reference (Φ_Δ_ = 0.56 in DMF,^46^ Φ_F_ = 0.32 in THF^47^) or Rose Bengal (Φ_Δ_ = 0.47 in DMF; for porphyrin derivatives only)^48^ were employed. The experimental details can be found in the Supporting Information.

The fluorescence emission spectra in both DMF and water were mirror images of particular absorption spectra (Figure 2b,d,f) with Stokes shifts of approximately 10 nm, which is a typical value for Pcs and related macrocycles.^49,50^ In PBS and water, however, the fluorescence signal was often weaker when aggregation occurred (see Figure 2b, red line).

Most of compounds exhibit high singlet oxygen production (Φ_Δ_ ∼ 0.50–0.60) while retaining reasonable fluorescence emission (Φ_F_ ∼ 0.20–0.30). This is true for anionic derivatives with −SO_3_H groups (**A6-A8Zn**), all compounds from the N-series (**N1-N11**), and all cationic derivatives **C1-C15Zn** (except **C6Zn**, which suffers from partial aggregation in DMF). Both Φ_Δ_ and Φ_F_ substantially decreased in anionic **A1-A5Zn** bearing COONa, which was caused by solubility issues associated with these derivatives in DMF. Similar problems were faced before,^17^ and the quantum yields had to be determined for the corresponding free acids, which reached values comparable to those observed for most of the PSs in this study (e.g., Φ_Δ_ = 0.42 and 0.45; Φ_F_ = 0.19 and 0.12 for **A3Zn­(COOH)** and **A4Zn­(COOH)**, respectively).^17^

The heavy atom effect^12^ is obvious because, compared with metal free **A9H**, magnesium **N2Mg**, and Al­(III) **A10Al**, zinc derivatives generally have higher quantum yields. On the other hand, the latter two have higher fluorescence emission. Notably, the counteranion seems to have no effect on Φ_Δ_ and Φ_F_ if complete monomerization is ensured. Thus, fully monomeric **C5Zn-I** and **C5Zn-PF**_**6**_ have identical photophysical parameters (Φ_F_ ∼ 0.27, Φ_Δ_ ∼ 0.47), whereas **C5Zn-SO**_**4**_ tends to aggregate (see comparison in Figure S44), leading to a significant decrease in values (Φ_F_ = 0.077, Φ_Δ_ = 0.28).

The Φ_Δ_ and Φ_F_ values of Si­(IV)­Pcs **Si1-Si5** were substantially lower despite being fully monomeric. For **Si5-N**, the reason is not entirely clear; in the case of **Si1-Si4** containing aliphatic amines, it is probably due to quenching of excited states via photoinduced electron transfer (PET) from the axial nitrogens to the macrocycle, as described previously.^35^ Consequently, PET leads to decreasing values of Φ_Δ_ = 0.005–0.23 and Φ_F_ = 0.055–0.25 only. Under physiological conditions, these nitrogens are protonated, restoring efficient singlet oxygen production and fluorescence emission as also demonstrated previously.^35^

The trends in the values of the fluorescence lifetimes (τ_F_) (see Table 1) correlated well with the values of Φ_F_ discussed above. The compounds with the highest Φ_F_ have τ_F_ of ∼3 ns, and the higher the Φ_F_, the greater is the τ_F_. If the quenching process occurred (PET or aggregation caused by solvent effects), biexponential decay with an additional fast component of τ_F_ < 1 ns was typically present.

In general, many of the studied Pcs had greater singlet oxygen production than the only clinically approved Pc for PDT (i.e., **Photosens**, Φ_Δ(DMF)_ = 0.25, Φ_F(DMF)_ = 0.62), but they had comparable or slightly lower Φ_Δ_ values than those of clinically approved porphyrins: **PpIX**, **verteporfin**, and **temoporfin** exhibited high singlet oxygen production (Φ_Δ(DMF)_ = 0.73–0.76, Φ_Δ(EtOH)_ = 0.51–0.67) because of slightly decreased fluorescence emission (Φ_F(DMF)_ = 0.12–0.24). Despite this, Pcs are a promising group of PSs because of their much stronger absorption (ε typically an order of magnitude higher than that of porphyrins) in the optical window of biological tissues, which enables the use of lower light or drug doses for efficient activation.

### Interaction with BSA

To obtain deeper insight into biologically relevant media, the interaction of the compounds with serum proteins were investigated since serum proteins play important roles in the biodistribution of drugs, affecting their distribution, efficacy, and elimination. Albumin, which is the most abundant one, is well-known to strongly bind anionic drugs, mainly into the positively charged binding sites formed predominantly by basic amino acid residues (Lys, Arg, and His).^51^ This finding is in accordance with several records in the literature, which show that anionic Pcs strongly bind to BSA^17^ or aid in the specific disassembly of anionic Pc nanostructures.^52^ The amount of BSA was chosen to mimic the typical concentration of BSA in serum-containing medium (35 μM), half of that amount (17.5 μM), and an excess (100 μM).

Spectral studies performed in PBS with different amounts of BSA (Figures S46–S89) revealed that almost all types of derivatives were affected by BSA in some way. In the case of the aggregated compounds of the A-series, the addition of BSA aided in the partial (**A2Zn**, **A6Zn**, and **A9H**) or complete (**A1Zn**, **A7Zn**, and **A8Zn**) disaggregation of the compounds into monomerized compounds, which was also obvious from increased fluorescence emission (Figure 3a). The addition of BSA to nonaggregating derivatives (**A3Zn**, **A4Zn**, **A5Zn**, **A10Al**) led to a slight bathochromic shift in the Q-band accompanied by partial quenching of the excited states, which was observed as a decrease in the Q-band maximum and fluorescence intensity (Figure 3b), which was negligible in the case of Pcs (**A3Zn**, **A4Zn**, and **A10Al**) but significant in the case of the aza analog **A5Zn**. Thus, although interaction with BSA increases the monomeric level of anionic Pcs, it can lead to quenching of the excited states of some of them (in particular, AzaPcs), which in turn can worsen the final photophysical properties, as has been shown for symmetric monomeric substances.

**Figure 3:**
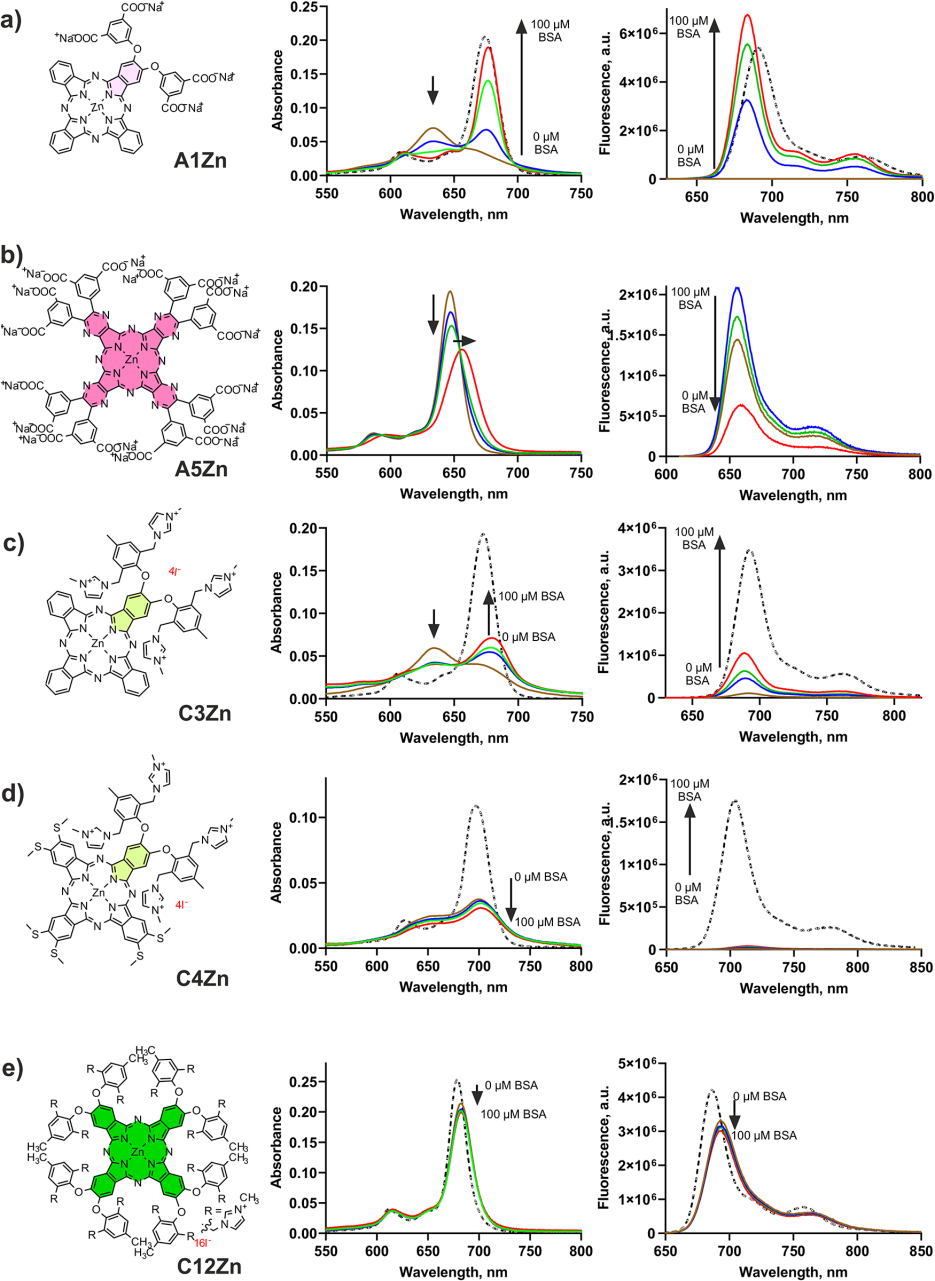
Representative examples of changes in absorption and fluorescence emission spectra upon interaction with BSA. Spectra of 1 μM Pc in PBS (brown line) were taken, and then, BSA was added to attain final concentrations of 17.5 μM (blue line), 35 μM (green line), and 100 μM (red line) in a cuvette. For comparison, spectra in monomeric state (DMSO (a), DMF (c–e)) are added as dashed black lines. Compound **A5Zn** is not well soluble in organic solvent but monomeric already in PBS. Spectra of all compounds of the series are shown in Supporting Information Figures S45–S87.

Different behaviors were observed in the C-series, where complete monomerization was not observed for any of the aggregated derivatives, indicating rather limited interactions with BSA. Partial monomerization evident from changes in the absorption spectra and an increase in fluorescence emission occurred in the case of strongly aggregated low-symmetrical **C1Zn**, **C3Zn**, and **C15Zn** (Figure 3c). In the case of less aggregated derivatives (**C4Zn**, **C5Zn**, and **C8Zn**), the interaction of BSA led to a slight decrease in the absorption band (without a shift in the spectrum) but was accompanied by a slight increase in the fluorescence intensity (Figure 3d), which indicates a probable effect of monomerization. Nevertheless, the level of monomerization was rather low for the C-series. These findings are in agreement with recent publication on the interactions of a series of cationic Pcs with different numbers of cationic functions with BSA, where rather limited spectral changes were observed even at high (200 μM) concentrations of BSA.^53^ Finally, the absorption and fluorescence emission spectra of monomeric derivatives of the C-series (**C7Zn**, **C9Zn**, and **C11Zn-C14Zn**) were almost unaffected (Figure 3e), which proves that, in contrast to anionic derivatives, cationic derivatives do not bind significantly with BSA.

Similarly to the C-series, the ability of BSA to monomerize aggregated Pcs was also evident in the N-series, where the fluorescence was slightly enhanced for the strongly aggregated compounds **N2Mg**, **N10Zn**, and **N11Zn**, although no changes were observed in their absorption spectra. In the case of less aggregated **N1Zn** and **N3-N5Zn**, monomerization was apparent even from the absorption spectra, again with a significant increase in the monomeric form documented by the increase in the fluorescence intensity. The addition of BSA to the monomeric **N6-N9Zn** derivatives resulted in no change in spectral properties, similar to those observed for monomeric cationic species, demonstrating that the interaction of uncharged Pcs with BSA did not occur.

Owing to the monomeric character of the Si-series, their interactions with BSA correspond to those of the above-mentioned derivatives in the fully monomeric state. Thus, the absorption spectra of cationic Si­(IV)­Pcs were not affected by the presence of BSA; only in the case of **Si3-C** was a decrease in the Q-band accompanied by fluorescence quenching. On the other hand, the addition of BSA to aggregated **Si5-N** enhanced fluorescence emission, while the absorption spectra remained unchanged, again indicating rather limited interaction.

In summary, interactions with BSA seem to be important, particularly for the A-series. It may quench the excited states of some PSs (which is rather an exception) but simultaneously aid in the monomerization of aggregated species, thus increasing the number of photoactive species. Therefore, even aggregated PSs may become active in biological environments. Notably, the attachment of Pcs to proteins may, however, result in strong chemical quenching of produced singlet oxygen by its reaction with susceptible amino acids in BSA as the closest target.^17^ These crucial cytotoxic species may not be amenable to the destruction of other biomolecules in cells. Once the Pcs are attached to the BSA, they are taken up by cells through endocytosis in this complex.

### *In Vitro* Studies

The photophysical properties and spectral analysis of Pcs provide useful insights for predicting photodynamic activity; however, such parameters are not always fully predictive, because of the complexity of the biological environment. Additional factors—including cellular uptake and localization, interactions with serum proteins and biomembranes, and variations in pH across different cellular compartments—can substantially influence biological outcomes. To account for these variables, all of the Pcs examined in this study were subjected to biological evaluation to better define the structural features relevant to PDT activity.

Detailed *in vitro* studies involving subcellular localization and determination of the EC_50_ in three different cell lines (human cervical carcinoma—HeLa, human breast adenocarcinoma—MCF-7, and human skin melanoma—SK-MEL-28) were performed. A broadband light source (ozone-free 450 W Xe-lamp, Newport) equipped with the long-pass filter Schott OG570 was used (12.4 mW/cm^2^, 15 min, 11.2 J/cm^2^). This setup provided a stable irradiance between 600 and 800 nm (see Figure S129), thereby minimizing variability in the activation of different Pcs and enabling reliable comparison of results across compounds. The *in vitro* findings are summarized in Table 2 and Figures 4–6 and S88–S128.

**Table 2:** Photodynamic Activity of All Derivatives Assessed on HeLa (Human Cervical Carcinoma), MCF-7 (Human Lung Carcinoma), and SK-MEL-28 (Human Skin Melanoma) Cell Lines

Cpd.	EC_50_ (nM) HeLa	Ref^a^	EC_50_ (nM) MCF-7	Ref^a^	EC_50_ (nM) SK-MEL-28	Ref^a^	Subcellular localization (HeLa)	Ref^a^
**A1Zn**	290 ± 78	^17^	453 ± 26	^17^	530 ± 30	t.w.	Ly, Me	t.w.
**A2Zn**	410 ± 156	^17^	584 ± 88	^17^	700 ± 50	t.w.	Ly, Me	t.w.
**A3Zn**	5160 ± 1030	^17^	3470 ± 670	^17^	2000 ± 300	t.w.	Ly	t.w.
**A4Zn**	10 310 ± 1020	^17^	5090 ± 1240	^17^	12 700 ± 2400	t.w.	Ly^17^	t.w.
**A5Zn**	5700 ± 1100	^54^	3010 ± 740	^17^	10 800 ± 2100	t.w.	Ly^54^	t.w.
**A6Zn**	12 000 ± 3000	t.w.	22 100 ± 6600	t.w.	10 400 ± 3600	t.w.	Ly	t.w.
**A7Zn**	1300 ± 400	t.w.	5100 ± 350	t.w.	2120 ± 560	t.w.	Ly	t.w.
**A8Zn**	1000 ± 300	t.w.	2470 ± 810	t.w.	1200 ± 280	t.w.	Ly	t.w.
**A9H**	85 ± 7	t.w.	347 ± 125	t.w.	300 ± 77	t.w.	Ly, Me	t.w.
**A10Al**	2070 ± 290	^55^	2040 ± 310	^17^	1900 ± 200	t.w.	Ly	t.w.
**C1Zn**	27 ± 9	^17^	35 ± 2	^17^	22 ± 3	t.w.	Ly, Me	^17^
**C2Zn**	105 ± 34	^10^	65 ± 8	^10^	62 ± 11	t.w.	Ly, Me	^10^
**C3Zn**	48 ± 19	^17^	21 ± 8	^17^	40 ± 4	t.w.	Ly, Me	^17^
**C4Zn**	79 ± 20	^10^	61 ± 9	^10^	108 ± 24	t.w.	Ly, Me	^10^
**C5Zn-I**	62 ± 13	t.w.	60 ± 10	t.w.	110 ± 10	t.w.	Ly	t.w.
**C5Zn-PF**_**6**_	52 ± 26	t.w.	100 ± 20	t.w.	190 ± 40	t.w.	Ly	t.w.
**C5Zn-SO**_**4**_	53 ± 9	t.w.	80 ± 30	t.w.	150 ± 50	t.w.	Ly	t.w.
**C6Zn**	58 ± 20	t.w.	110 ± 30	t.w.	150 ± 50	t.w.	Ly	t.w.
**C7Zn**	480 ± 250	^24^	50 ± 14	^24^	1880 ± 370	t.w.	Ly	^24^
**C8Zn**	540 ± 90	^25^	280 ± 40	t.w.	320 ± 48	^25^	Ly	t.w.
**C9Zn**	310 ± 121	^25^	210 ± 10	t.w.	220 ± 21	^25^	Ly	^25^
**C10Zn**	148 ± 45	t.w.	186 ± 76	t.w.	524 ± 152	t.w.	Ly	t.w.
**C11Zn**	3.8 ± 0.2	^55^	2.8 ± 0.1	^55^	3.8 ± 0.6	^55^	Ly	^55^
**C12Zn**	37 ± 6	^26^	11 ± 3	^17^	45 ± 7	t.w.	Ly	^26^
**C13Zn**	12 ± 4	^27^	5.3 ± 0.8	^27^	56 ± 6	t.w.	Ly	^27^
**C14Zn**	110 ± 27	^24^	47 ± 16	^24^	64 ± 2	t.w.	Ly	^24^
**C15Zn**	74 ± 22	t.w.	50 ± 8	t.w.	25 ± 6	t.w.	Ly	t.w.
**N1Zn**	17 ± 5	t.w.	6 ± 1	t.w.	16 ± 3	t.w.	Mi	t.w.
**N2Mg**	107 ± 1	t.w.	190 ± 40	t.w.	119 ± 14	t.w.	Ly	t.w.
**N3Zn**	2200 ± 160	^30^	1500 ± 300	^30^	900 ± 200	^30^	Ly	t.w.
**N4Zn**	2070 ± 60	^30^	1200 ± 100	^30^	500 ± 100	^30^	Ly	t.w.
**N5Zn**	4970 ± 930	^30^	2400 ± 300	^30^	1030 ± 160 ^30^	t.w.	Ly	^30^
**N6Zn**	69 700 ± 10 700	^30^	233 000 ± 6000	t.w.	305 000 ± 29 000	t.w.	Ly	t.w.
**N7Zn**	75 700 ± 17 100	^30^	228 000 ± 9000	t.w.	306 000 ± 46 000	t.w.	Ly	t.w.
**N8Zn**	354 000 ± 42 000	^32^	287 000 ± 47 000	t.w.	183 000 ± 44 000	t.w.	Ly	t.w.
**N9Zn**	163 000 ± 66 000	^32^	170 000 ± 23 000	t.w.	189 000 ± 34 000	t.w.	Ly	t.w.
**N10Zn**	540 ± 80	t.w.	430 ± 60	t.w.	360 ± 50	t.w.	Ly	t.w.
**N11Zn**	560 ± 80	t.w.	31 ± 12	t.w.	45 ± 3	t.w.	Ly	t.w.
**Si1-C**	49.2 ± 26.0	t.w.	11 ± 4	t.w.	11 ± 3	t.w.	Ly	t.w.
**Si2-C**	80 ± 30	t.w.	54 ± 7	t.w.	49 ± 6	t.w.	Ly	t.w.
**Si3-C**	10 ± 3	t.w.	13 ± 4	t.w.	11 ± 3	t.w.	Ly, Mi	t.w.
**Si4-C**	2.7 ± 0.6	t.w.	5.5 ± 1.3	t.w.	7.5 ± 1.1	t.w.	Ly	t.w.
**Si5-N**	2.5 ± 0.3	t.w.	9.2 ± 2.4	t.w.	6.6 ± 1.1	t.w.	Mi	t.w.
**PpIX**	5500 ± 600	t.w.	5700 ± 800	t.w.	6200 ± 300	t.w.		
**verteporfin**	36 ± 10	^55^	33 ± 3	t.w.	21 ± 3	t.w.		
**temoporfin**	45 ± 7	^55^	210 ± 74	t.w.	103 ± 17	t.w.		

a The data with literature references have been determined previously under the same experimental conditions at the same workplace and published. t.w. = this work. Cells were incubated with the compounds for 12 h, washed, and subsequently irradiated with a Xe-lamp for 15 min (λ > 570 nm, 11.2 J/cm^2^). Results are expressed as mean ± SD. Ly, lysosomes; Me, membrane; Mi, mitochondria. EC_50_ values are reported in the same range (nM) for easy comparison of photodynamic activities among all compounds. For values using only significant figures, please see Figures S88–S93.

**Figure 4:**
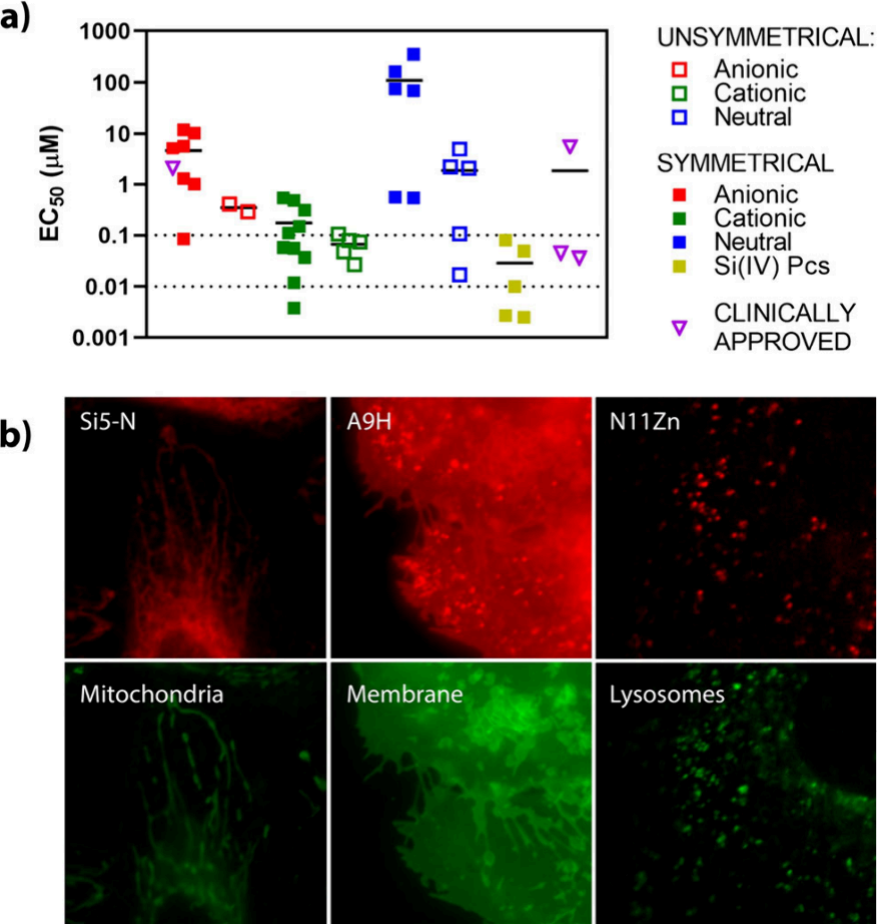
*In vitro* experiments on HeLa cells: (a) depiction of EC_50_ values of all studied compounds grouped by their structural characteristics (charge, position of substituents); silicon derivatives represent separate groups (analogical data for MCF-7 and SK-MEL-28 cell lines can be found in Supporting Information Figure S122). Compound **A10Al** (Photosens) is included in the “symmetrical anionic” group (red full squares) and marked as a purple open triangle (clinically approved); (b) subcellular localization of selected derivatives localizing into mitochondria (**Si5-N**), cytoplasmic membrane (**A9H**), and endolysosomal compartment (**N11Zn**); red–photosensitizer, green–organelle-specific probes.

Initial analysis was performed by using HeLa cells, although the observations are generally applicable to the other cell lines examined. The results revealed an extremely wide range of photodynamic activity with EC_50_ (HeLa) ranging from 2.5 nM to 354 μM with no consistent correlation with the determined Φ_Δ_ (Figure 5a). For example, the symmetrical derivative **A4Zn** was monomeric in water and strongly produced singlet oxygen in DMF (Φ_Δ_ = 0.45) but had relatively low photodynamic activity (EC_50_ = 10.31 μM, HeLa cells). In contrast, structurally related unsymmetrical **A2Zn** with an identical peripheral group reached 25× higher photodynamic activity under identical conditions (EC_50_ = 0.41 μM) despite being aggregated in water and having half the value of Φ_Δ_ in DMF (Φ_Δ_ = 0.22). Direct correlations of the EC_50_ values with the Φ_Δ_ or log *P* parameters (Figure 5) did not yield clear structure–activity relationships. The only consistent trend was the enhanced photodynamic activity observed for the N-series with increasing lipophilicity (Figure 5b, blue). These findings highlight the necessity of a broader, multifactorial interpretation of the results.

**Figure 5:**
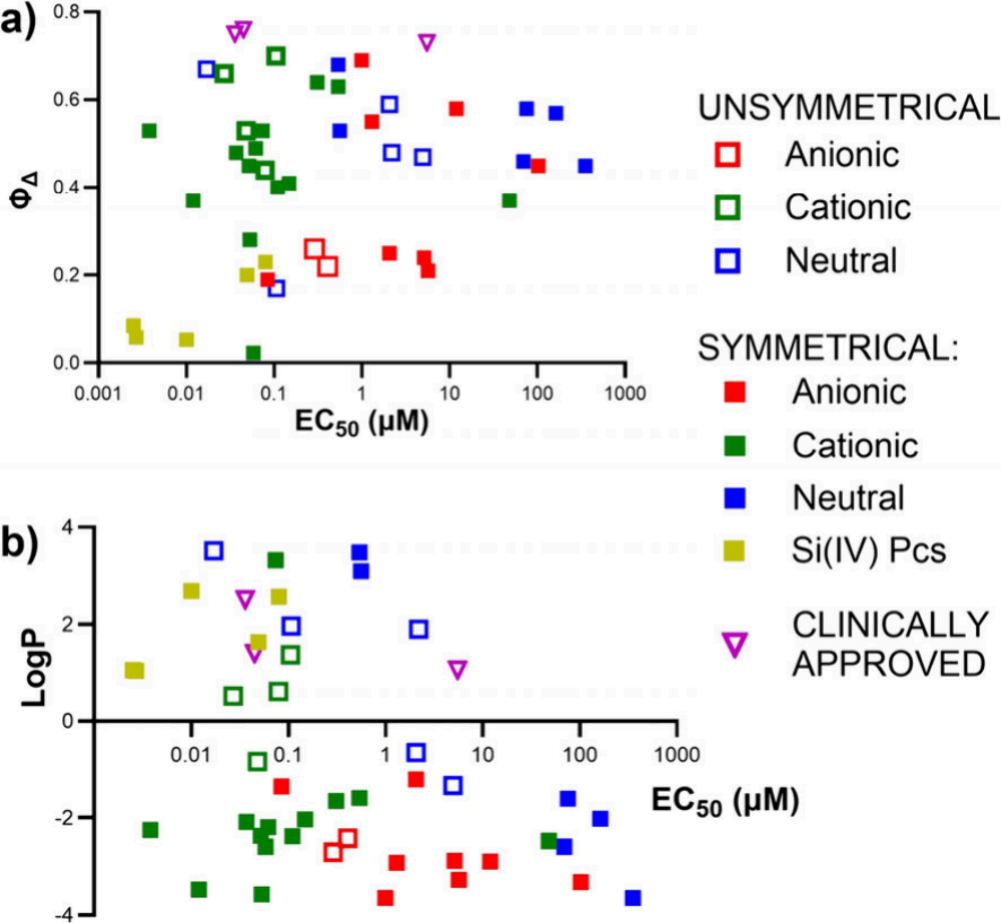
Correlation of EC_50_ (HeLa) with Φ_Δ_ (a) or log *P* (b) parameters.

For a long time, subcellular localization was deemed to be one of the critical factors of the resulting PDT effect.^56^ This was largely attributed to the limited diffusion distance of singlet oxygen from its site of generation.^57^ It has been repeatedly demonstrated that the localization of PSs within target cells influences not only the photodynamic activity but also the predominant type of cell death (together with the light dose).^58^ Therefore, the subcellular localization of all the studied derivatives was assessed in the HeLa cell line (human cervical carcinoma), the most widely used model cell line.^59^ Nearly all of the derivatives investigated in this study were predominantly localized in endolysosomal compartments (Figures 4b, S94–S97, S99–S105, and S107–S117) with several compounds also detected in additional organelles. Notably, **Si3-C** exhibited dual localization in both lysosomes and mitochondria (Figures 4b and S116). Moreover, compounds **Si5-N** (Figure S118) and **N1Zn** (Figure S106) were the only PSs that were not detected in lysosomes at all, indicating exclusive mitochondrial localization. Cytoplasmic membrane localization (in addition to that of lysosomes) was observed with some charged unsymmetrical Pcs (**A1Zn**, **A2Zn**, and **C1-C4Zn**) and low-symmetry **A9H** (Figures 4B and S98). Localization to adiposomes,^60^ the Golgi apparatus,^61^ nuclei,^62^ or other organelles was not detected, unlike for some other PSs, PS-containing delivery systems, or PS containing targeting moieties reported in the literature.

Given that almost all of the derivatives were localized to lysosomes, subcellular localization can hardly be used as a parameter for predicting photodynamic activity, especially considering the wide range of EC_50_ values of derivatives localized in lysosomes, starting at 2.5 nM (**Si5-N**) and reaching values as high as 354 μM (**N8Zn**). Although PSs localizing to mitochondria are among the most active derivatives (**N1Zn**, **Si3-C**, and **Si6-N** with EC_50_ values of 17 nM, 10 nM, and 2.5 nM, respectively), this localization is not determining the high activity, as derivatives such as **Si4-C** (2.7 nM), **C11Zn** (3.8 nM), or **C13Zn** (12 nM) are solely localized to lysosomes but still retain comparable photodynamic activity upon irradiation.

With respect to peripherally decorated Pcs, most members of the **A-** and **N-series** are less active than those of the **C-series** compounds. The overall situation when the compounds are divided into subgroups (symmetrical, unsymmetrical, cationic, anionic, and neutral) is shown in Figure 4, and subsequent subanalyses of the specific situations (type of function and flexibility) are shown in detail in Figure 6 (including statistical analysis) and are described in the following paragraph.

**Figure 6:**
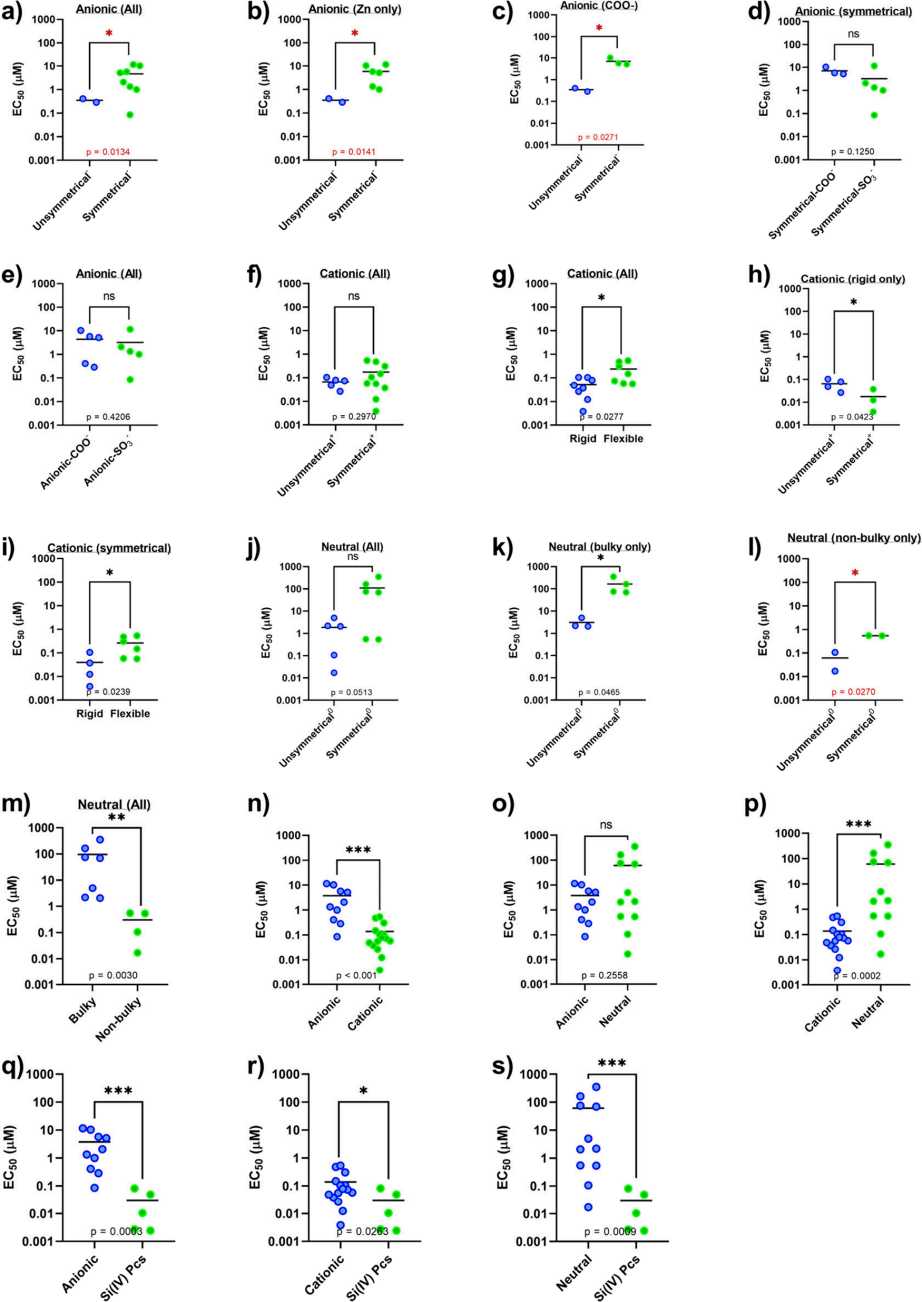
Comparison of the photodynamic activities of different groups of photosensitizers against HeLa cells. Analysis (*t* test) is marked red if at least one group contains only two members. (a–e) Anionic compounds, (f–i) cationic compounds, and (j–m) neutral compounds. (n–s) Comparison of all main groups. For a detailed description of the statistical analysis, see the Supporting Information.

The difference between the **C-series** and the other two series was statistically significant (Figure 6n,p), whereas the difference between the **A-series** and **N-series** was not significant (Figure 6o). For both **A-** (Figure 6a–c) and **N-series** (Figure 6j–l), symmetrical derivatives were generally less active than unsymmetrical derivatives, whereas the **C-series** compounds exhibited the opposite trend (Figure 6h); however, it was strongly dependent on whether the comparison is made with compounds with similar types of substituent (i.e., in a rigid or flexible arrangement (see below)). Notably, unlike the two other series, all of the anionic derivatives strongly interacted with BSA, which may explain their weaker effect. One of the literature studies also reported that much higher activity of anionic Pcs in vitro was observed when the cells were treated in serum-free medium, whereas no difference was observed in cationic derivatives.^17^

With respect to the **A-series**, amphiphilic anionic derivatives (**A1Zn** and **A2Zn**) were ∼21 times more active than their symmetrical hydrophilic counterparts (**A3Zn** and **A4Zn**), which can be attributed to the preservation of the monomeric state because of the interaction of unsymmetrical derivatives with the membranes,^17^ and this was also consistent when the whole series was analyzed (Figure 6a). Water-soluble symmetrical anionic derivatives may be negatively influenced by low intralysosomal pH via charge neutralization and subsequent aggregation in this environment, as we previously demonstrated.^17^ On the other hand, this does not have to be the only factor since no improvement was observed in those Pcs bearing more acidic sulfonate functions (Figure 6d,e).

In the **N-series**, the PDT activity correlated well with the lipophilicity expressed as log *P* (Figure 5b). An increase in activity was observed with less hydrophilic unsymmetrical analogs compared with their fully hydrophilic symmetrical analogs: **N3Zn** (2.20 μM), **N4Zn** (2.07 μM), and **N5Zn** (4.97 μM) versus **N6Zn** (69.71 μM) and **N8Zn** (354 μM) (Figure 6j–l). The bulkiness and high polarity of the substituents seem to play significant roles (Figure 6m), as they represent obstacles to efficient cellular uptake. It has also been independently reported in the literature recently that symmetrical derivatives of the **N-series** with lower activity had substantially lower cellular uptake than their unsymmetrical more lipophilic derivatives.^30,63,64^ This is further supported by the highest photodynamic activity of unsymmetrical derivatives with nonbulky moieties **N1Zn** and **N2Mg** with EC_50_ values of 17 and 107 nM, respectively, which are significantly more lipophilic, with log *P* ≥ 2. They are closely followed by symmetrical nonbulky derivatives **N10Zn** and **N11Zn**. Statistical analysis of the **N-series** containing either bulky or nonbulky moieties also revealed significant differences between unsymmetrical and symmetrical compounds (Figure 6k,l), which might be a consequence of the lower lipophilicity of the latter.

The activity of cationic derivatives (**C-series**) was among the highest in this investigation and was affected by two main structural characteristics: the bulkiness and rigidity of peripheral moieties (Figure 6g) and, as outlined above, the introduction of amphiphilic character. Moieties in cationic derivatives that are rather small (e.g., in **C8Zn** and **C9Zn**) are flexibly linked to the core, and their position relative to the core may change, leading to inefficient protection against aggregation in aqueous environments. Consequently, they were characterized by decreased activity in this series (EC_50_ = 540 and 310 nM, respectively; Figure S89). Compounds bearing more bulky moieties that are still flexible (e.g., **C5Zn-I**, **-PF**_**6**_, and **-SO**_**4**_) had lower EC_50_ values (62, 52, and 53 nM, respectively; Figure S89). However, derivatives with very bulky moieties in a rigid arrangement (e.g., **C11Zn** and **C13Zn**) do not enable repositioning of the charges relative to the core (therefore possessing great protection against aggregation) and represent very potent PSs with extremely low EC_50_ values (3.8 and 12 nM, respectively; Figure S90). Therefore, unlike the **N-series** (Figure 6m), increasing the bulkiness of the moieties increases the photodynamic activity of the **C-series** derivatives because of their rigid arrangement (Figure 6g). The second difference is in the behavior of the unsymmetrical analogs (Figure 6h), where the statistical analysis indicates better results for the symmetrical Pcs. In direct comparison, unsymmetrical cationic derivatives (e.g., **C1Zn** and **C2Zn**, one cationic moiety bearing 3 charges) were slightly less potent than their symmetrical counterparts (**C11Zn**, four cationic moieties bearing 12 charges in total). Similar results were also obtained from the comparison of unsymmetrical **C3Zn** and **C4Zn** (two cationic substituents with 4 charges in total) with symmetrical **C12Zn** analogs (eight cationic moieties with 16 charges in total). Notably, the activity of **C5Zn-I**, **-PF**_**6**_, and **-SO**_**4**_ did not significantly differ; therefore, the influence of these counterions on the photodynamic activity is likely very low (Figure S119).

Silicon derivatives stand out from the whole series because they are not decorated with moieties on the periphery but contain axial substituents linked to the central Si­(IV) atom (Figure 1). Owing to this feature, all Si­(IV)­Pcs are well protected against aggregation, leading to very efficient PSs with the best activities (with EC_50_ values ranging from 2.5 to 80 nM), which were significantly better than those of the **A-** or **N-series** and with comparable results to those of the whole **C-series** (Figure 6q,r,s) (Table 2, Figure S92).

In general, **C-series** and **Si-series** were more potent PSs than the **A-series** and **N-series**, and the **A-series** vs **N-series** did not significantly differ (Figure 6n–s).

Notably, all the above-mentioned results were obtained using the HeLa cell line, but two other malignant cell lines were used to further support the behavior of all the studied derivatives: the human breast carcinoma cell line MCF-7 and the human skin melanoma cell line SK-MEL-28. Comparisons between the effects of the analogs on these cell lines and statistical analyses revealed similar trends (Figures S120 and S121), which were sometimes even more pronounced: e.g., the increase in the activity of **C12Zn** in comparison with that of **C3Zn** was just 1.3× greater in HeLa cells but 2× and 12× greater in MCF-7 and SK-MEL-28 cells, respectively. However, overall, the EC_50_ values were comparable between the cell lines (Table 2, Figures S88–S93) with no significant difference when the whole series was considered (Figure S123), although for particular derivatives, small differences were observed (Figures S124–S128).

Notably, some of the clinically approved photosensitizers were included in this study: porphyrin-based PSs **verteporfin**, **temoporfin**, and **PpIX** (the active form of the prodrug δ-aminolaevulinic acid and its derivatives) and the above-mentioned **Photosens** (**A10Al**). While the photodynamic activity of **PpIX** (EC_50_ = 5.5 μM) was similar to that of anionic symmetrical analogs (including **A10Al**), **verteporfin** and **temoporfin** reached reasonably high photodynamic activity comparable, e.g., with some cationic derivatives with EC_50_ values of 36 nM and 45 nM, respectively (Table 2, Figures S88–S93). The activity of **Photosens** perfectly corresponded to the relationships derived for anionic symmetrical Pcs (see Figure 4a).

## Conclusions

There are a number of factors that may affect the (photo)­toxicity and subsequent usability of any drug in treatment. This extensive study of different types of Pcs under the same experimental conditions enabled us to define some of the factors influencing the spectral, photophysical, and PDT properties. Compared with porphyrins and chlorins, which form a typical group of clinically approved PSs, Pcs absorb strongly in the Q-band area (ε ∼ 150–300 000 M^–1^ cm^–1^), which is advantageous and may lead to a stronger PDT effect and a reduction in the dose of PS during cancer treatment. A significant red-shift of the Q-band can be achieved by the introduction of substituents to nonperipheral (α-) positions, which may further increase the therapeutic impact of PSs since red light penetrates deeper into tissues.

Owing to the planar macrocycles, the lipophilic *Pc* core tends to aggregate in aqueous medium. A comparison of the Pcs in this series clearly revealed that the most efficient way to achieve monomerization is to either use Si­(IV)­Pcs or introduce charged bulky groups into a rigid arrangement. Furthermore, aggregation may be partially suppressed by BSA present in biological media, especially in the case of anionic derivatives, whose strongest interactions are based on electrostatic interactions. The photophysical parameters of the monomeric forms of the studied Pcs (in DMF) were almost identical within the whole series with Φ_Δ_ ∼ 0.50–0.60 and Φ_F_ ∼ 0.20–0.30, following well-known rules such as the heavy atom effect, higher Φ_Δ_ for nonperipherally substituted derivatives, and quenching by PET (in the case that an amine is present in the molecule).

There are no measurable in-solution parameters that can be used to simply predict PDT activity. More key structural factors must be combined to obtain effective PSs. Notably, this may change if carriers are used to transport the PSs. The use of carriers, supramolecular assemblies, and various drug delivery systems as alternative ways to modify different parameters of PSs, such as aggregation, cellular uptake, localization, and ROS generation at the site of action, was not the aim of this study. The key findings from this study can be summarized as follows:

- Cationic Pcs generally possess higher activity (lower EC_50_ values) than anionic and neutral Pcs probably because of limited binding to BSA and other factors described elsewhere.^17^
- Si­(IV)­Pcs are superior PSs in general because they effectively protect against aggregation because of axial substituents.
- Introduction of rigid bulky substituents to the periphery is the key tool to achieve full monomerization for all types of Pcs (anionic, cationic, or neutral). Bulkiness increases the PDT activity of cationic derivatives but decreases activity of neutral derivatives.
- Interaction with biomolecules (such as serum albumin) may help in monomerization but also partially quenches the excited states of some derivatives, particularly aza-analogues. Interaction with BSA also seems to be the cause for the lower activity of anionic Pcs.
- Low-symmetry character resulting in amphiphilic molecules substantially improves the activity of neutral and anionic derivatives but has rather limited effects on cationic derivatives.

On the basis of these observations, several recommendations for the design of Pcs for PDT can be proposed to maximize the effects. Axial substitution in SiPcs is favorable for modification, despite lower singlet oxygen production. In other metal complexes, the optimal strategy depends on the nature of the substituents. For neutral groups (e.g., PEG, sugars, and alcohols), smaller substituents are favored, maintaining high lipophilicity while retaining sufficient water solubility to enable cellular application. Cationic derivatives generally exhibit high photodynamic activity; to further enhance efficacy, bulky and rigid substituents positioned above and below the macrocyclic core are recommended with no clear requirement regarding symmetrical versus unsymmetrical substitution. In the case of anionic derivatives, unsymmetrical substitution, leading to amphiphilic structures, is preferable.

The results of this study provide a general framework for structural features that may influence *Pc*-mediated PDT activity *in vitro*. However, it remains challenging to consolidate these findings into a universal design principle, as multiple factors can contribute to the overall effect. Therefore, the conclusions presented here should be regarded as guiding considerations rather than definitive rules and are intended to serve as a basis for further critical evaluation in the design of new PSs.

## Experimental Section

### General

The UV/vis spectra were recorded on a Shimadzu UV-2600 spectrophotometer (Shimadzu, Kyoto, Japan). The fluorescence spectra were recorded on a FLS-1000 or FS-5 Photoluminescence Spectrometer (Edinburg Instruments, Edinburg, United Kingdom). Statement of purity: The present study utilized compounds that have been previously synthesized and characterized in peer-reviewed publications. As these compounds underwent prior analytical validation and their purity was verified during the original studies, additional purity testing was not repeated herein. The samples were employed as obtained from the cited sources under the assumption that their reported purity was adequate for the intended evaluations.

### Sample Source

All of the compounds were obtained from the authors of their first publication. The synthesis and source of the compounds are mentioned Table 1.

### Absorption and Emission Spectra

A 100 μM stock solution of the Pc was prepared in water (**A3Zn**, **A4Zn**, **A5Zn**, **A7Zn**, **C5**, and **C6Zn**), DMF/water 10:1 (**A1Zn**, **A2Zn**, and **C9Zn**), or DMF (all other samples of the series). An absorption spectrum of 2.475 mL of solvent (water, DMF, or PBS) was taken (i.e., absorption spectrum of baseline); then, 25 μL of stock solution was added to reach the final concentration of 1 μM in the cuvette, and the absorption and emission spectra were taken. Baseline of the solvent was subtracted from the obtained absorption spectrum to get the final absorption spectrum of the sample. The excitation wavelengths were as follows: 575 nm (**N7Zn**, **PpIX**), 600 nm (**A5Zn**, **verteporfin**, **temoporfin**), 605 nm (**C5Zn-I**, **C5Zn-PF**_**6**_, **C5Zn-SO**_**4**_, **C10Zn**, **Si1-C**, **Si2-C**), 610 nm (**A1Zn**, **A2Zn**, **A3Zn**, **A6Zn**, **C1Zn**, **C3Zn**, **C12Zn**, **C13Zn**, **C15Zn**, **N3Zn**, **N4Zn**, **N5Zn**, **N8Zn**, **Si3-C**, **Si4-C**, **Si6-N**), 613 nm (**N6Zn**), 615 nm (**C6Zn**, **C7Zn**, **C14Zn**), 624 nm (**A9H**, **A10Al**, **N1Zn**, **N2Mg**, **N10Zn**, **N11Zn**, **Si5-N**), 625 nm (**A7Zn**, **A8Zn**), 630 nm (**C2Zn**, **C4Zn**, **C8Zn**, **C11Zn**, **N9Zn**), 635 nm (**A4Zn**), and 665 nm (**C9Zn**).

### Determination of Quantum Yields of Singlet Oxygen Production

The quantum yields of singlet oxygen were calculated using the comparative method with unsubstituted zinc­(II) phthalocyanine (ZnPc) as a reference (Φ_Δ(ZnPc)_ = 0.56 in DMF^46^) and monitored using the decomposition of 1,3-diphenylisobenzofuran (DPBF) as a ^1^O_2_ scavenger. The detailed procedure involved the transfer of 2.5 mL of stock solution of DPBF in DMF (5 × 10^–5^ M) into a 10 × 10 mm quartz cuvette, and it was bubbled with oxygen for 40 s. Eight μL of the 100 μM stock solution of the dye in DMF (or DMF + water or water; see above) was added. A xenon lamp (100 W, ozone free XE DC short arc lamp, New port) was used to irradiate the sample while stirring. The incident light was filtered through a water filter (6 cm) and cut off filter OG530 eliminating heat and light under 523 nm, respectively. For the porphyrin derivatives (**PpIX**, **verteporfin**, and **temoporfin**), a 20CGA-455 filter, and Rose Bengal (RB) as the reference (Φ_Δ(RB)_ 0.68 in DMF^48^) were used. The decrease of DPBF in solution with irradiation time was monitored at 415 nm. The values of Φ_Δ_ of the dyes were calculated by the following equation:

$$\phi_{\Delta }^{S}=\phi_{\Delta }^{R}\left(\frac{k^{S}I_{aT}^{R}}{k^{R}I_{aT}^{S}}\right)$$

where *k* is the slope of the plot of the dependence of $\ln\left(\frac{A_{0}}{A_{t}}\right)$ on irradiation time *t* with *A*_0_ and *A*_*t*_ being the absorbances of the DPBF at 415 nm before irradiation and after irradiation time *t*, respectively. *I*_aT_ is the total amount of light absorbed by the dye sample. Superscripts R and S indicate the reference and sample, respectively. *I*_aT_ is calculated as a sum of intensities of the absorbed light *I*_a_ at wavelengths from 523 to 850 nm (step 0.5 nm). Light under 523 nm is completely filtered by an OG530 filter, and light above 850 nm is not absorbed by the studied dye sample. *I*_a_ at a given wavelength is calculated using Beer–Lambert’s law as

$$I_{a}=I_{0}\left(1-e^{-2.3A}\right)$$

where *I*_0_ is transmittance of the filter at the given wavelength and *A* is the absorbance of the dye at this wavelength. All experiments were performed three times, and the data presented is the mean value of the three obtained results with estimated error ± 10%.

### Determination of Fluorescence Quantum Yields

The fluorescence quantum yields (Φ_F_) were determined by a comparative method with ZnPc as the reference (Φ_F_ = 0.32 in THF^47^). The Φ_F_ values were calculated using the following formula:

$$\phi_{F}^{S}=\phi_{F}^{R}\left(\frac{F^{S}}{F^{R}}\right)\left(\frac{1-10^{-A^{R}}}{1-10^{-A^{S}}}\right){\left(\frac{\eta^{S}}{\eta^{R}}\right)}^{2}$$

where *F* is the integrated area under the emission spectrum and *A* is the absorbance at the excitation wavelength. Superscripts R and S indicate the reference and sample, respectively. η stands for the refractive index of the solvent. Absorption in the Q-band was kept below 0.05 to eliminate the inner filter effect. Excitation wavelengths were the same as those used for emission spectra (see above). The emission wavelengths were set to their emission maxima to avoid exceeding the detector limit with stimated error ± 15%.

### Study of Interaction with Bovine Serum Albumin (BSA)

To a 1 μM solution of the dye in PBS (0.01 M phosphate buffer, 0.0027 M potassium chloride, and 0.137 M sodium chloride, pH 7.4 in deionized water) in a quartz cuvette, prepared by dilution of the appropriate 100 μM stock solution of dye (see above), a 0.45 mM solution of BSA in PBS buffer was added sequentially to attain final concentrations of 17.5 μM, 35 μM, and 100 μM in the cuvette, and stirred for a few seconds at rt, and absorption and emission spectra were recorded after each addition.

### Determination of Log *P*

400 μL portion of *n*-octanol and 400 μL of PBS buffer were mixed in a plastic Eppendorf vial. Then, 20 μL of the 100 μM stock solution of the sample (see above) was added to this mixture, vortexed for 5 min at rt, and then centrifuged for 10 min (10 000 rpm, rt). 50 μL of each of these layers was taken into 2.5 mL of DMF in a quartz cuvette, and their emission spectra (excitation wavelengths in Table 1) were recorded. The log *P* value was then computed using the area under the curve of the emission spectra of *n*-octanol (*F*_oct_) and PBS buffer (*F*_PBS_) layers as follows:

$$\log P=\log\left(\frac{F_{oct}}{F_{PBS}}\right)$$

Triplicate measurements were performed, and the data reported are the mean of the obtained results.

### *In Vitro* Assessment of EC_50_ Values on HeLa, SK-MEL-28, and MCF-7 Cell Lines

For the cytotoxicity experiments (phototoxicity), cells were seeded at 100 μL per well into 96-well plates (TPP, Switzerland) at a density of 7.5 × 10^3^ (HeLa) or 1.0 × 10^4^ (MCF-7 and SK-MEL-28) cells per well. Cells were left to grow for 24 h (until they reached confluency) in a controlled environment of a CO_2_ incubator (37 °C, 5% CO_2_ atmosphere, constant high humidity). Subsequently, studied compounds were added in a wide concentration range and incubated with the cells for 12 h. Cells were washed; fresh cell culture medium was added, and the cells were irradiated using a 450 W Xe lamp (Newport, USA). The lamp was equipped with a long pass filter (Newport OG570) and an 8 cm water filter (λ > 570 nm, 12.4 mW/cm^2^, 15 min, 11.2 J/cm^2^). Cellular viability was assessed after an additional 24 h by the neutral red (NR; Sigma, Merck, USA) uptake assay. At least three independent experiments, each in triplicate, were performed. The soluble NR was measured at λ = 540 nm using a Tecan Infinite 200 M plate reader (Tecan, Austria). The viability of each experimental group was expressed as the percentage of the untreated cells (100% viability) after subtraction of the signal from cells treated with a lethal dose of hydrogen peroxide (0% viability). Results are shown on Figures S88–S93 and expressed as EC_50_ values (mean ± SD). EC_50_ values were calculated, and graphs were created by using GraphPad Prism 10.3.1 (Graph Pad software, USA).

### Subcellular Localization on HeLa Cells

Approximately 7.5 × 10^4^ HeLa cells were seeded on glass-bottom 35 mm Petri dishes suitable for confocal microscopy (WillCo Wells, The Netherlands) in SCM and incubated for 12 h with photosensitizers (concentration is listed in each respective image caption, Figures S94–S118) in a CO_2_ incubator. The medium was removed, and the cells were washed twice with a prewarmed fresh medium. LysoTracker Blue DND-22 (0.3 μM; Molecular Probes, Thermofisher Scientific) and MitoTracker Green FM (0.3 μM; Molecular Probes, Thermofisher Scientific) were added, and the cells were incubated for an additional 15 min. After incubation, the cells were rinsed twice with prewarmed medium, and the samples were immediately examined using a Nikon Eclipse Ti-E (Nikon, Japan) fluorescence microscope equipped with an Andor Zyla 5.5 cooled digital sCMOS camera (Andor Technology, United Kingdom) and NIS Elements AR 5.3 software (Laboratory Imaging, Czech Republic). DAPI, FITC, and Cy5 filter sets were used for visualization. NIS Elements AR 5.3 software was also used to create fluorescence intensity profiles for each acquired data set. Analogical workflow was also used for the determination of subcellular localization to the cytoplasmic membrane. Instead of probes for mitochondria and lysosomes, probes for cytoplasmic membrane (1× CellMask Plasma Membrane Stain; Invitrogen, Thermofisher Scientific) and nuclei (10 nM Hoechst 33342; Invitrogen, Thermofisher Scientific) were used.

## Supplementary Material





## Data Availability

The original photophysical data (absorption and emission spectra) and results from biological evaluation of the compounds have been deposited at Zenodo and are available at: https://zenodo.org/records/17284919.

## Notes

Absorption and emission spectra in DMF, water and PBS; changes in absorption and emission spectra upon interaction with BSA; *in vitro* assessment of EC_50_ values of HeLa, SK-MEL-28, and MCF-7 cell lines; subcellular localization on HeLa cells; graphs with statistical analysis; spectrum of lamp used for sample irradiation during *in vitro* studies (PDF)
Molecular formula strings and associated biological data (CSV)

## Abbreviations

^1^O_2_: singlet oxygen
BSA: bovine serum albumin
EC_50_: half-maximal effective concentration
Pcs: phthalocyanines
PDT: photodynamic therapy
PS: photosensitizer
TLC: thin layer chromatography
Φ_Δ_: quantum yield of singlet oxygen production
Φ_F_: quantum yield of fluorescence
τ_F_: fluorescence lifetime
