# Supplementary material for: Identifying Structural Factors Governing the Photodynamic Activity of Phthalocyanines
Source: J Med Chem. 2026 Feb 6;69(4):4391–407. doi: 10.1021/acs.jmedchem.5c03090 (PMC12951568; doi:10.1021/acs.jmedchem.5c03090)
Supplement: Supplementary file 1 [file jm5c03090_si_001.pdf]

Supporting Information

Identifying Structural Factors Governing the Photodynamic Activity of Phthalocyanines

Magdalena Kozlikova,[a‡] Mary Angelia Alfred,[a‡] Miloslav Machacek,[a‡*] Fabienne Dumoulin,[b,c] Andrés de la Escosura,[d] Tomasz Goslinski,[e] Marie Halaskova,[a] Jian-Dong Huang,[f] Mei-Rong Ke,[f] Saad Makhseed,[g] Dariusz T. Mlynarczyk,[e] Dennis K. P. Ng,[h] Tomás Torres,[d,i] Roy C. H. Wong,[h] Petr Zimcik,[a*] Veronika Novakova[a*]

‡ These authors contributed equally

[a]Faculty of Pharmacy in Hradec Kralove, Charles University, Ak. Heyrovskeho 1203, Hradec Kralove, 500 03, Czech Republic

[b]Faculty of Engineering and Natural Sciences, Department of Biomedical Engineering and Department of Chemistry, Acibadem Mehmet Ali Aydınlar University, Ataşehir, Istanbul 34752, Türkiye

[c]Acibadem Mehmet Ali Aydinlar University, Graduate School of Natural and Applied Sciences, Ataşehir, 34752 Istanbul, Türkiye

[d]Department of Organic Chemistry, Universidad Autónoma de Madrid, C/Francisco Tomás y Valiente 7, 28049 Madrid, Spain and Institute for Advanced Research in Chemistry (IAdChem), Cantoblanco

[e]Chair and Department of Chemical Technology of Drugs, Poznan University of Medical Sciences, Rokietnicka 3, 60-806, Poznań, Poland

[f]College of Chemistry, Fujian Provincial Key Laboratory of Cancer Metastasis Chemoprevention and Chemotherapy, Fuzhou University, Fuzhou, 350116, China

[g]Department of Chemistry, Kuwait University, PO Box 5969, Safat, 13060, Kuwait

[h]Department of Chemistry, The Chinese University of Hong Kong, Shatin, N.T., Hong Kong, 999077, China

[i]IMDEA-Nanociencia, Campus de Cantoblanco, 28049 Madrid, Spain.

Corresponding authors: machamil@faf.cuni.cz, veronika.novakova@faf.cuni.cz, zimcik@faf.cuni.cz

Content:

Content: S2

Absorption and fluorescence emission spectra in DMF, water and PBS S3

  Anionic derivatives S3

  Cationic derivatives S7

  Neutral derivatives S13

  Silicon derivatives S17

Comparison of absorption spectra of **C5Zn** with different counter anions S19

Interaction with bovine-serum albumin (BSA) S20

  Anionic derivatives S20

  Cationic derivatives S23

  Neutral derivatives S29

  Silicon derivatives S33

In vitro assessment of EC_{50} values on HeLa, SK-MEL-28 and MCF-7 cell lines S35

  Anionic derivatives S35

  Cationic derivatives S36

  Neutral derivatives S38

  Si(IV) Pcs derivatives S39

  Protoporphyrin IX, temoporfin and verteporfin S39

Subcellular localization on HeLa cells S40

  Anionic derivatives S40

  Cationic derivatives S42

  Neutral derivatives S44

  Si(IV) Pcs derivatives S46

Statistical analysis S48

  Comparing the effects of counterion using one-way ANOVA S48

  Comparing groups of photosensitizers using t-test S48

Overview of EC_{50} values S51

Overview of the differences among cell lines S51

Statistical evaluation of differences in EC_{50} values of individual compounds among cell lines using one-way ANOVA S52

Spectrum of the lamp for *in vitro* studies S54

References S55

Absorption and fluorescence emission spectra in DMF, water and PBS

Anionic derivatives

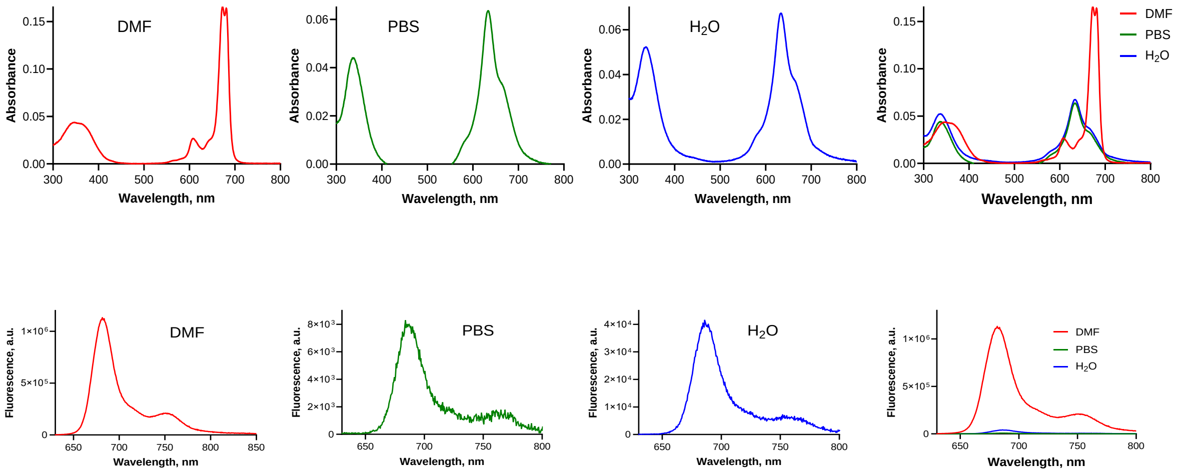

Fig. S1: Absorption and fluorescence emission spectra of **A1Zn** (1 µM) in DMF (red), PBS (green) and water (blue).

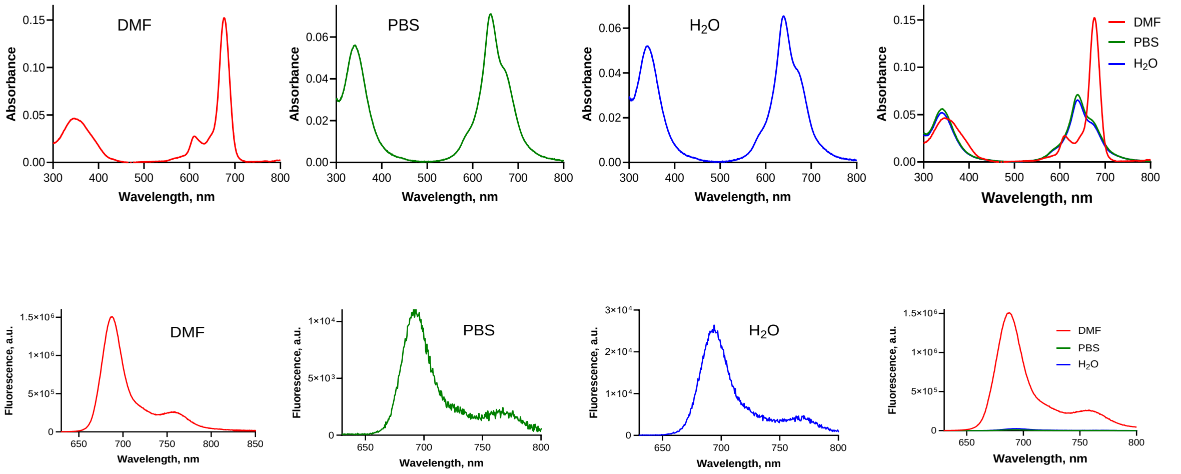

Fig. S2: Absorption and fluorescence emission spectra of **A2Zn** (1µM) in DMF (red), PBS (green) and water (blue).

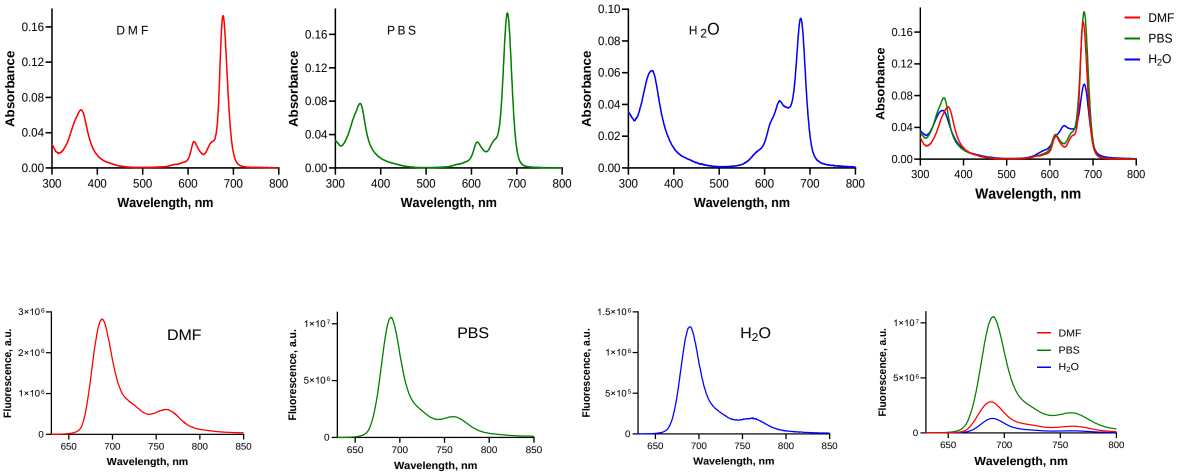

Fig. S3: Absorption and fluorescence emission spectra of **A3Zn** (1μM) in DMF (red), PBS (green) and water (blue).

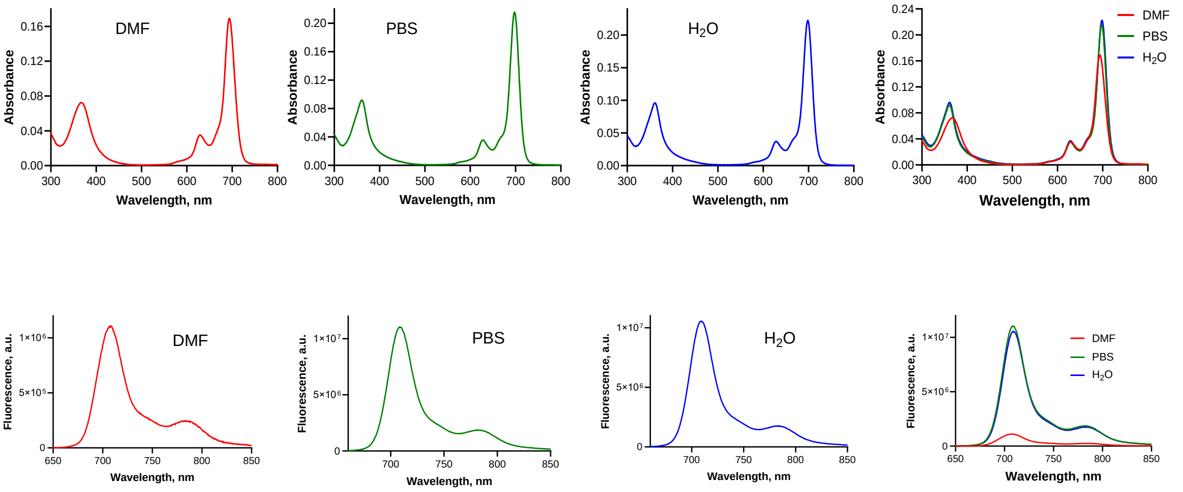

Fig. S4: Absorption and fluorescence emission spectra of **A4Zn** (1μM) in DMF (red), PBS (green) and water (blue).

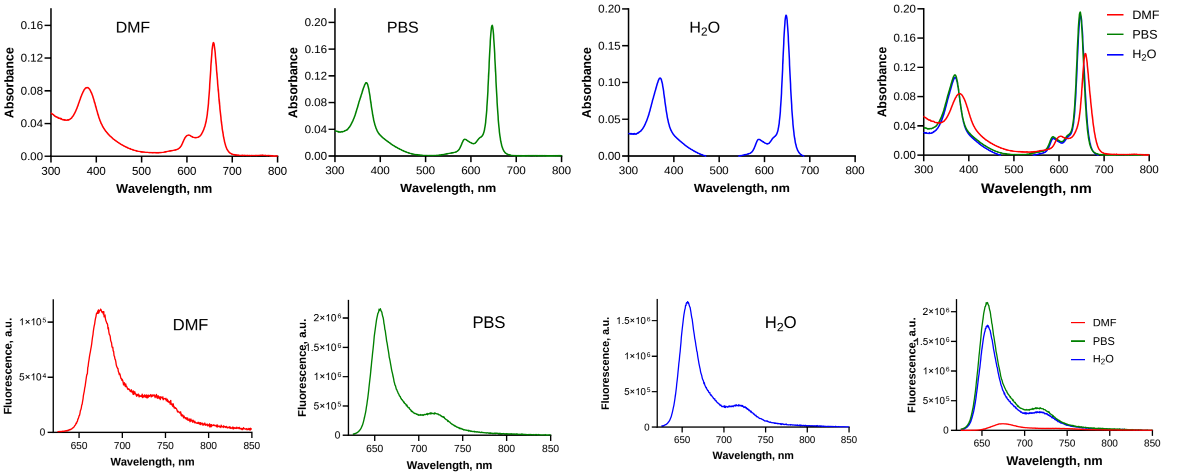

Fig. S5: Absorption and fluorescence emission spectra of **A5Zn** (1μM) in DMF (red), PBS (green) and water (blue).

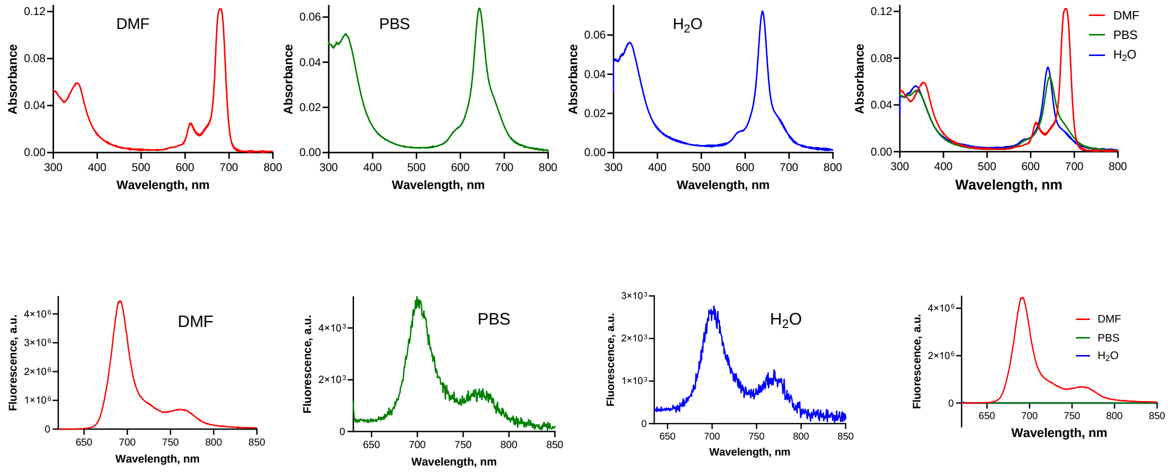

Fig. S6: Absorption and fluorescence emission spectra of **A6Zn** (1μM) in DMF (red), PBS (green) and water (blue).

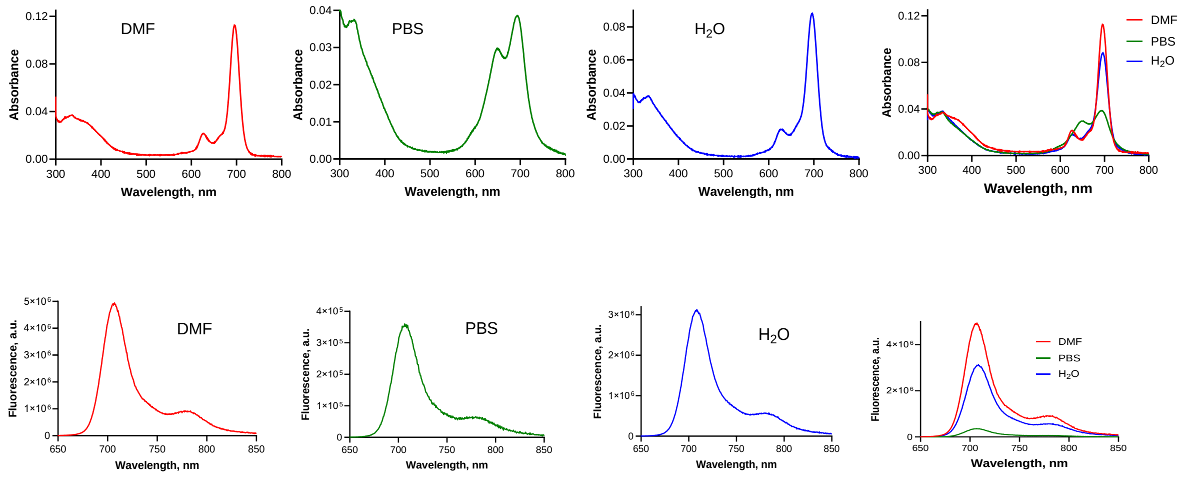

Fig. S7: Absorption and fluorescence emission spectra of **A7Zn** (1μM) in DMF (red), PBS (green) and water (blue).

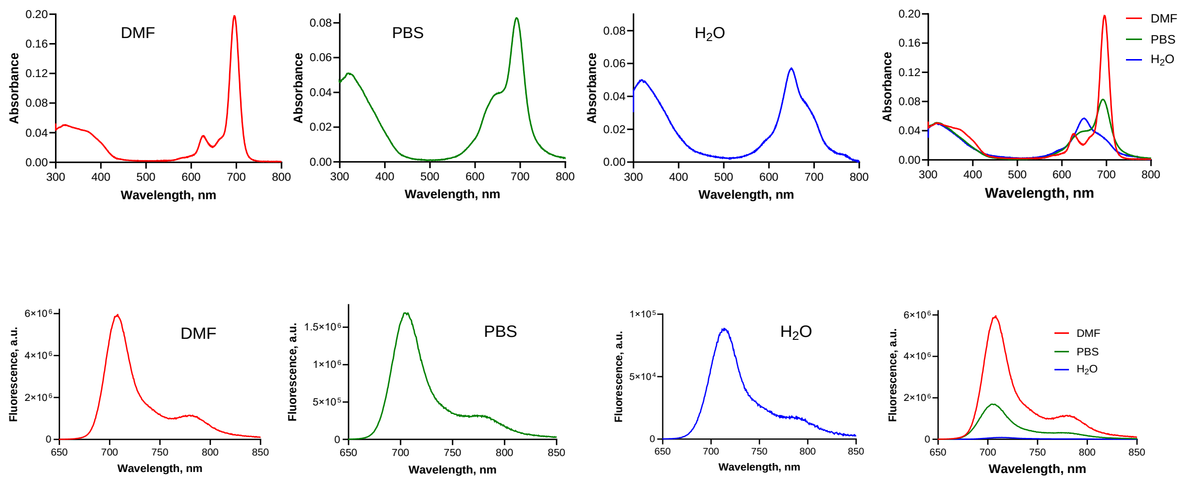

Fig. S8: Absorption and fluorescence emission spectra of **A8Zn** (1μM) in DMF (red), PBS (green) and water (blue).

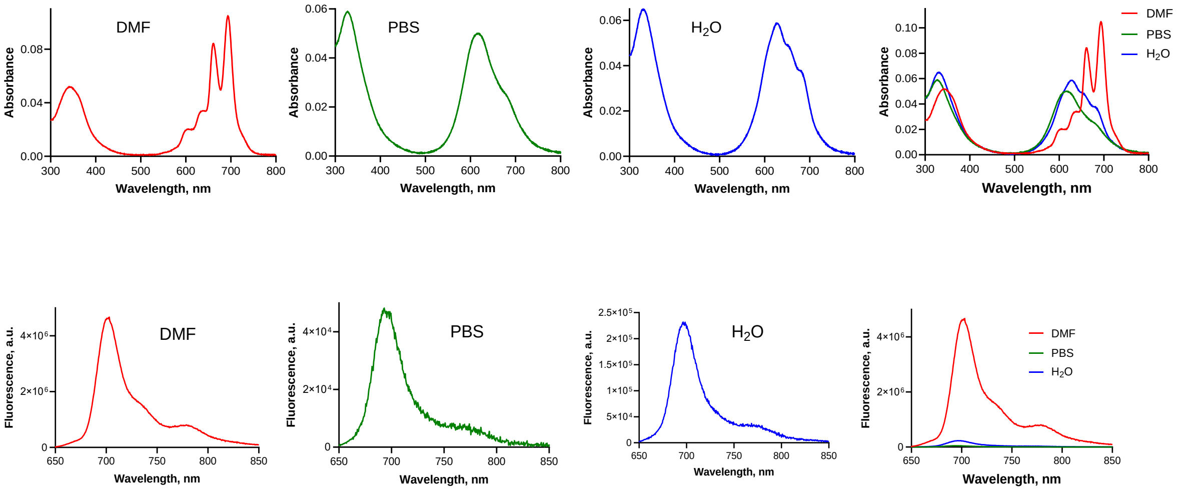

Fig. S9: Absorption and fluorescence emission spectra of **A9H** (1μM) in DMF (red), PBS (green) and water (blue).

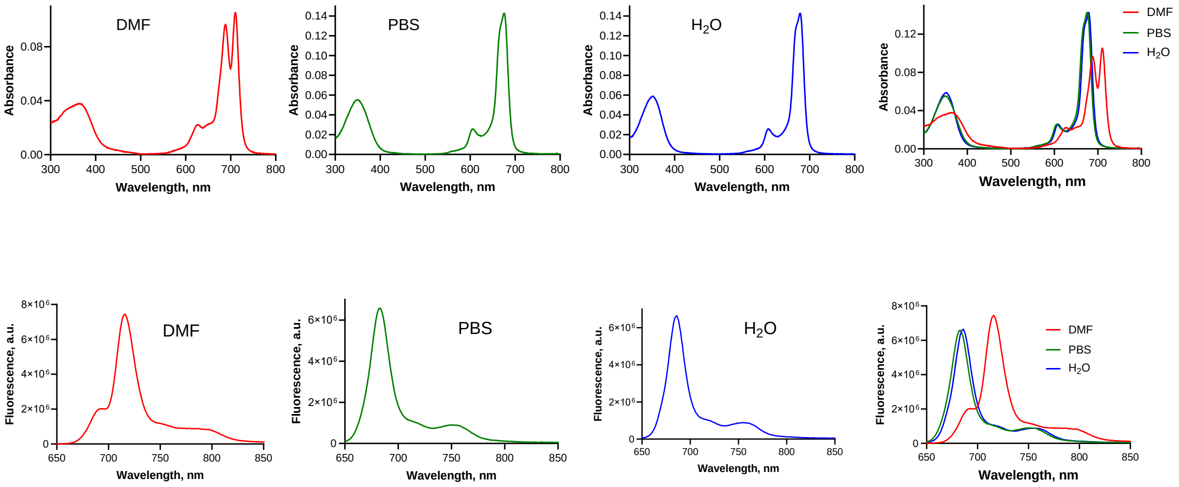

Fig. S10: Absorption and fluorescence emission spectra of **A10Al** (1μM) in DMF (red), PBS (green) and water (blue).

Cationic derivatives

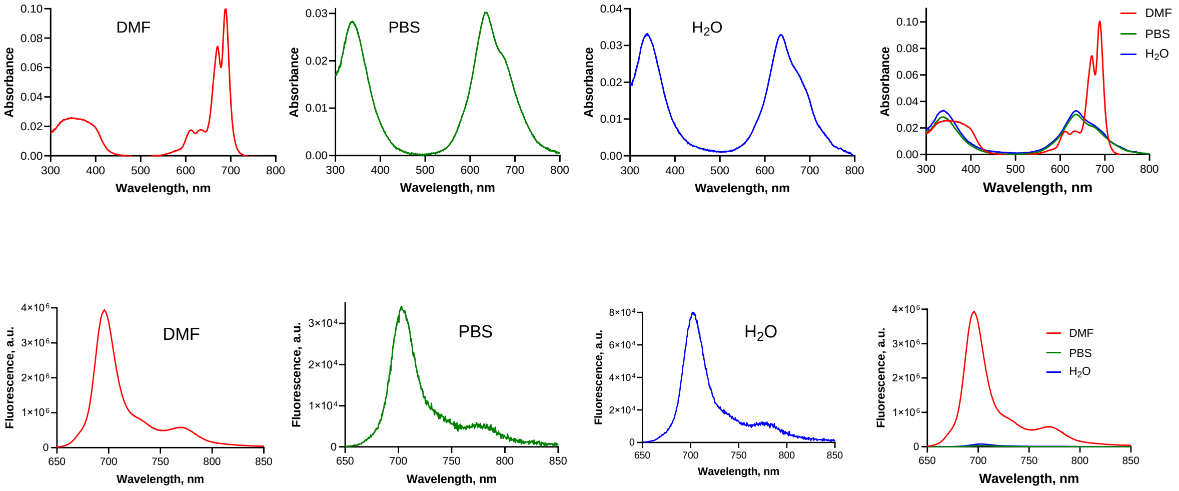

Fig. S11: Absorption and fluorescence emission spectra of **C1Zn** (1μM) in DMF (red), PBS (green) and water (blue).

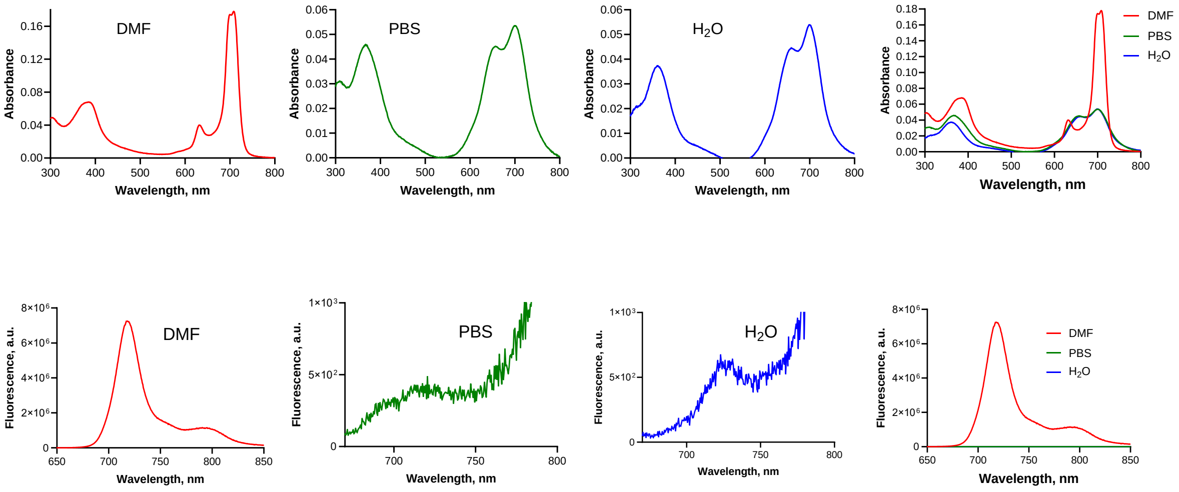

Fig. S12: Absorption and fluorescence emission spectra of **C2Zn** (1μM) in DMF (red), PBS (green) and water (blue).

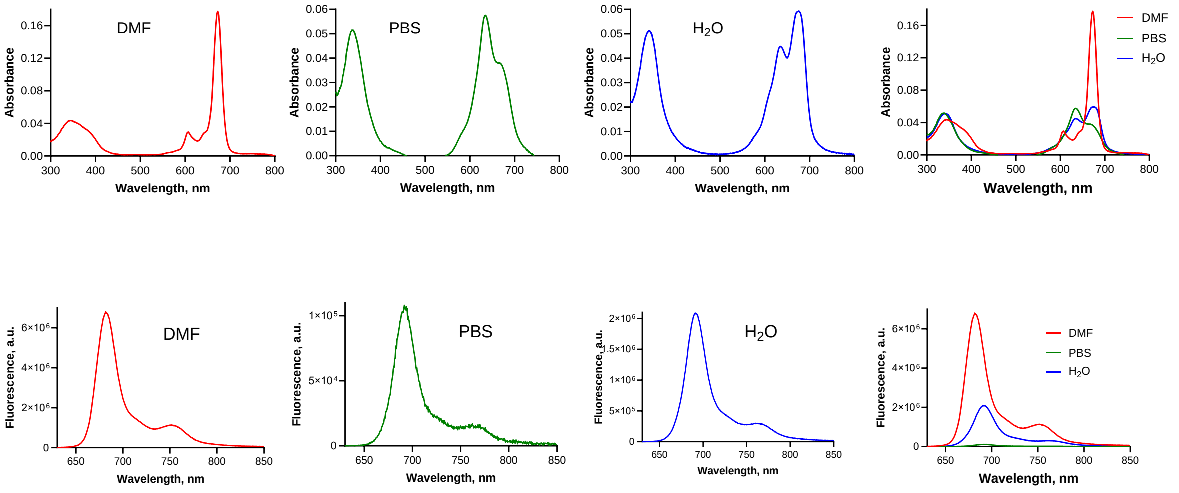

Fig. S13: Absorption and fluorescence emission spectra of **C3Zn** (1μM) in DMF (red), PBS (green) and water (blue).

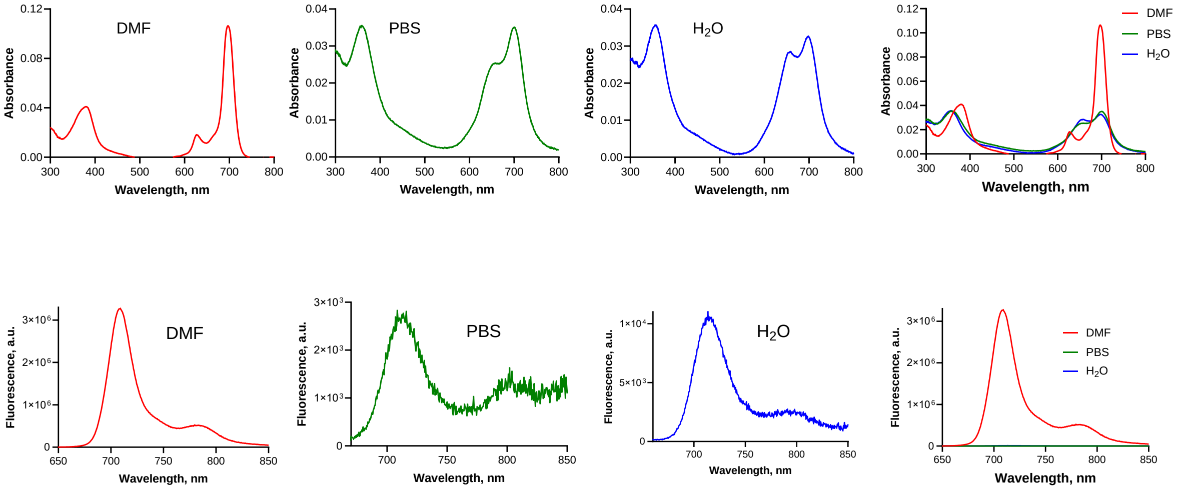

Fig. S14: Absorption and fluorescence emission spectra of **C4Zn** (1µM) in DMF (red), PBS (green) and water (blue).

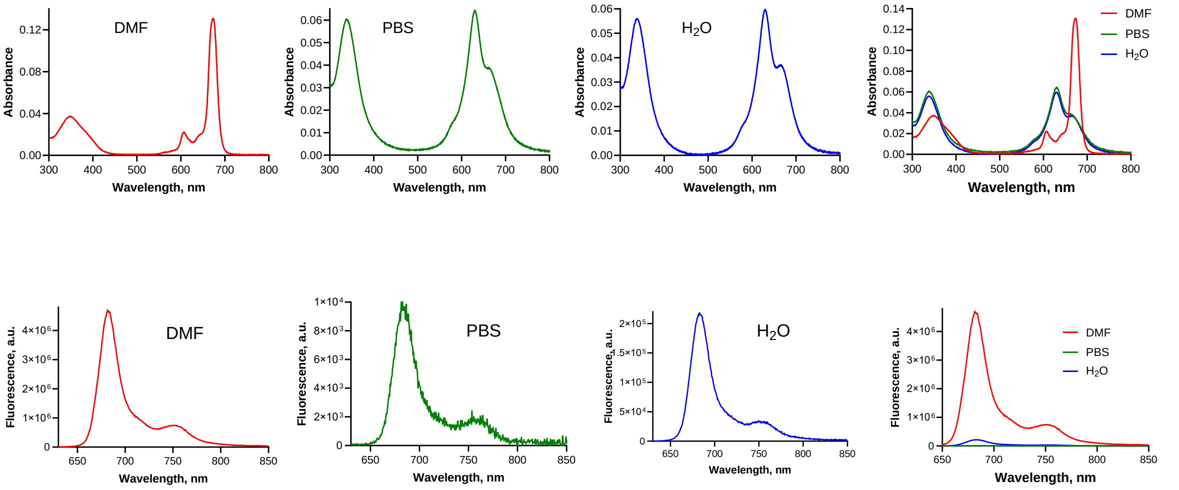

Fig. S15: Absorption and fluorescence emission spectra of **C5Zn-I** (1µM) in DMF (red), PBS (green) and water (blue).

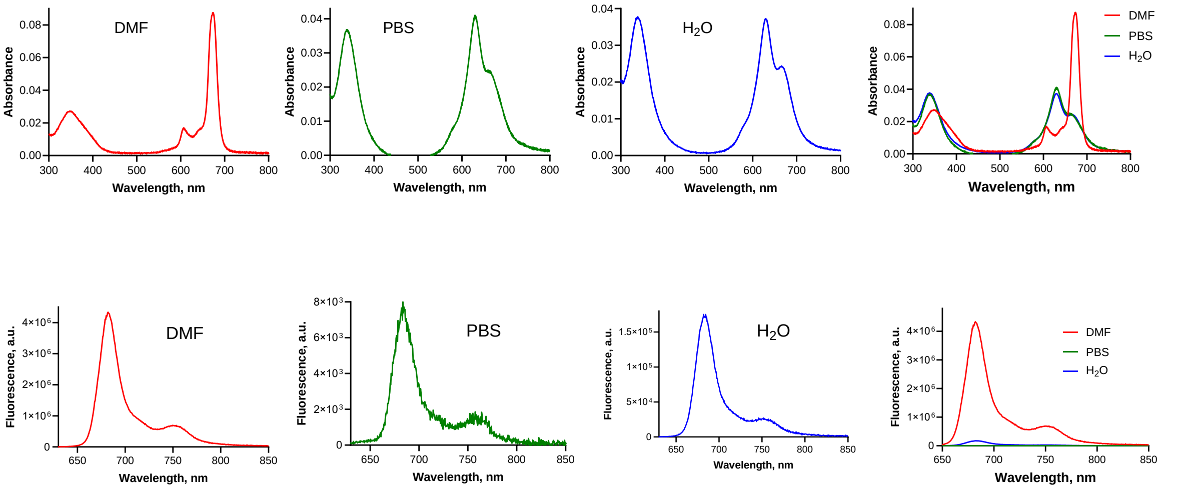

Fig. S16: Absorption and fluorescence emission spectra of **C5Zn-PF$_6$** (1µM) in DMF (red), PBS (green) and water (blue).

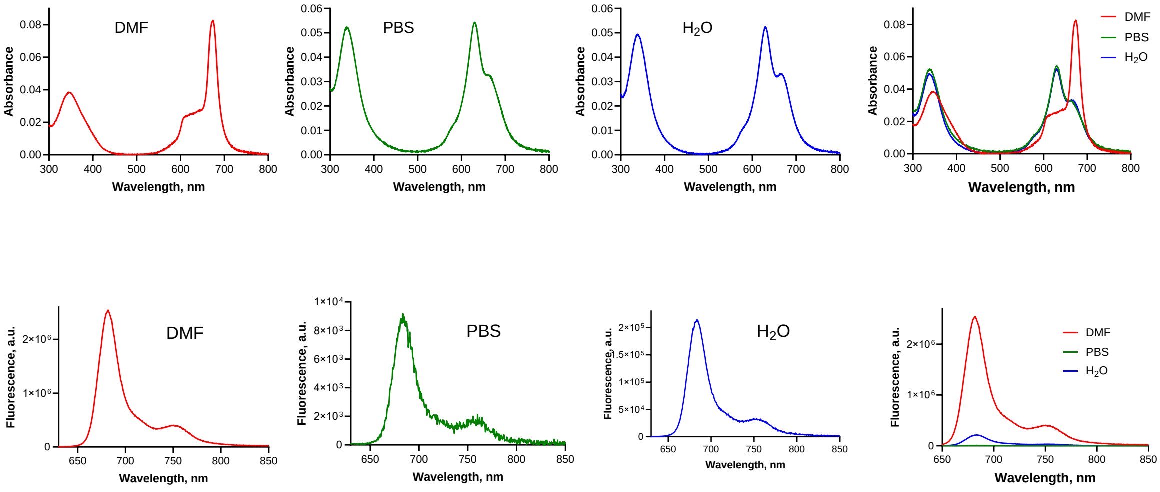

Fig. S17: Absorption and fluorescence emission spectra of **C5Zn-SO$_4$** (1µM) in DMF (red), PBS (green) and water (blue).

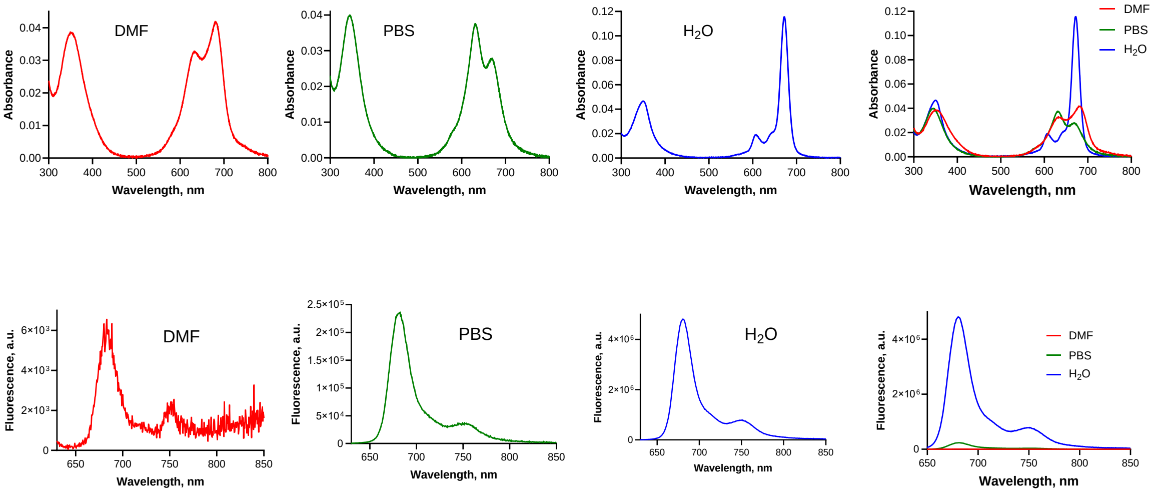

Fig. S18: Absorption and fluorescence emission spectra of **C6Zn** (1µM) in DMF (red), PBS (green) and water (blue).

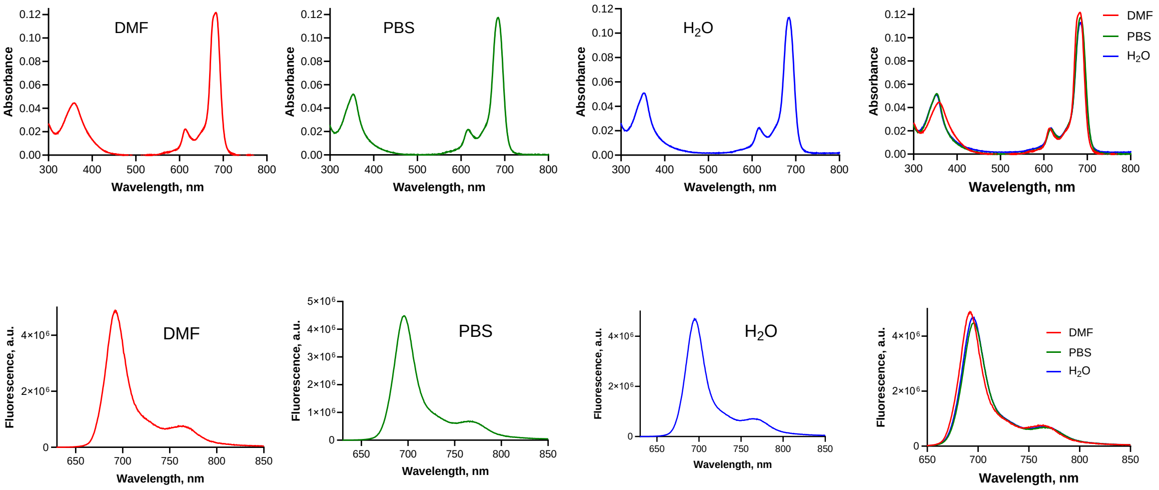

Fig. S19: Absorption and fluorescence emission spectra of **C7Zn** (1µM) in DMF (red), PBS (green) and water (blue).

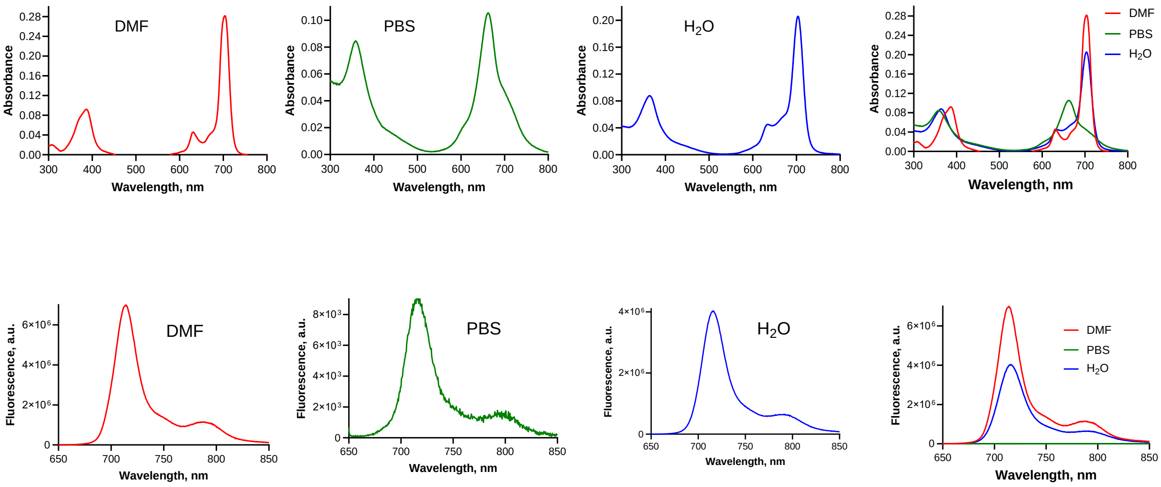

Fig. S20: Absorption and fluorescence emission spectra of **C8Zn** (1µM) in DMF (red), PBS (green) and water (blue).

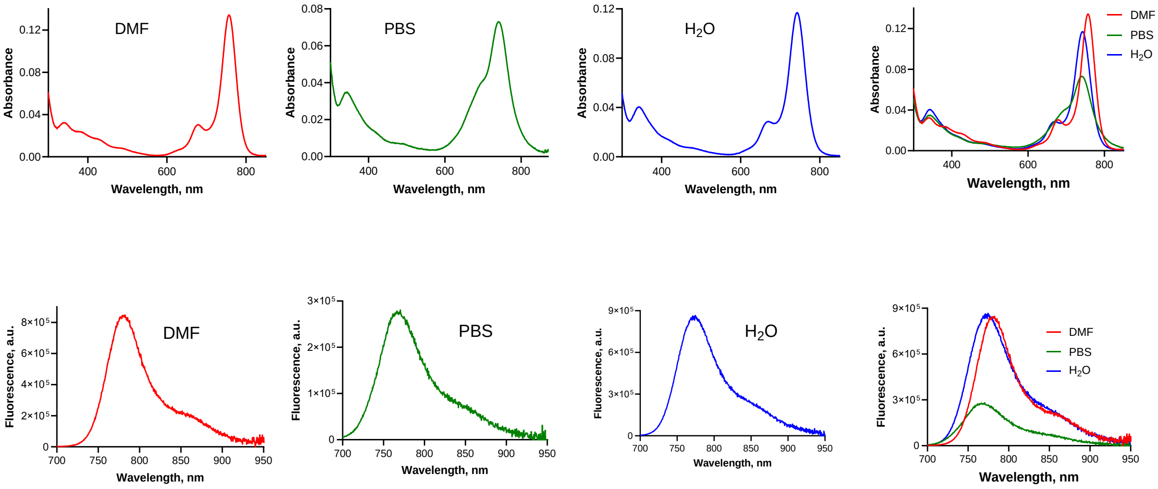

Fig. S21: Absorption and fluorescence emission spectra of **C9Zn** (1µM) in DMF (red), PBS (green) and water (blue).

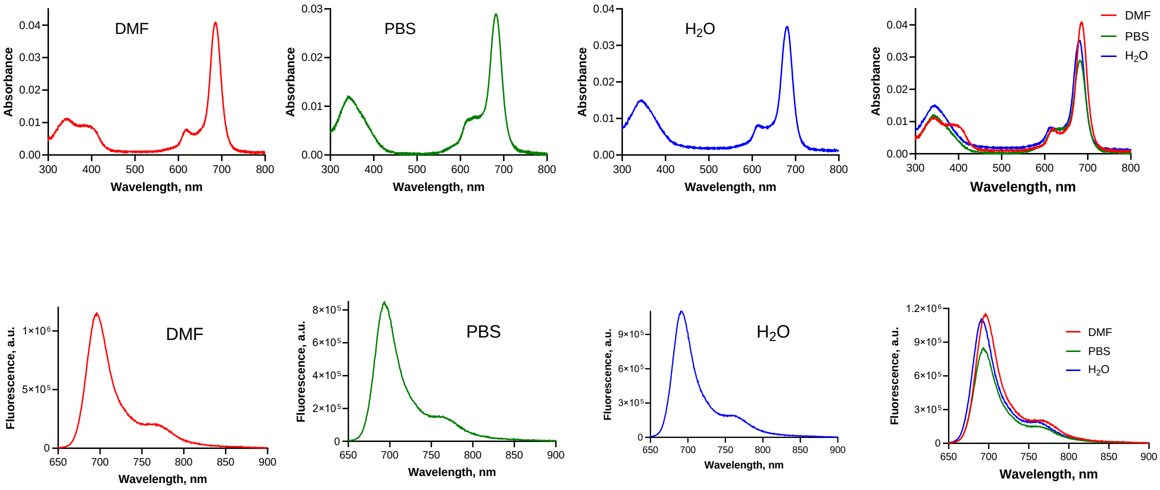

Fig. S22: Absorption and fluorescence emission spectra of **C10Zn** (1µM) in DMF (red), PBS (green) and water (blue).

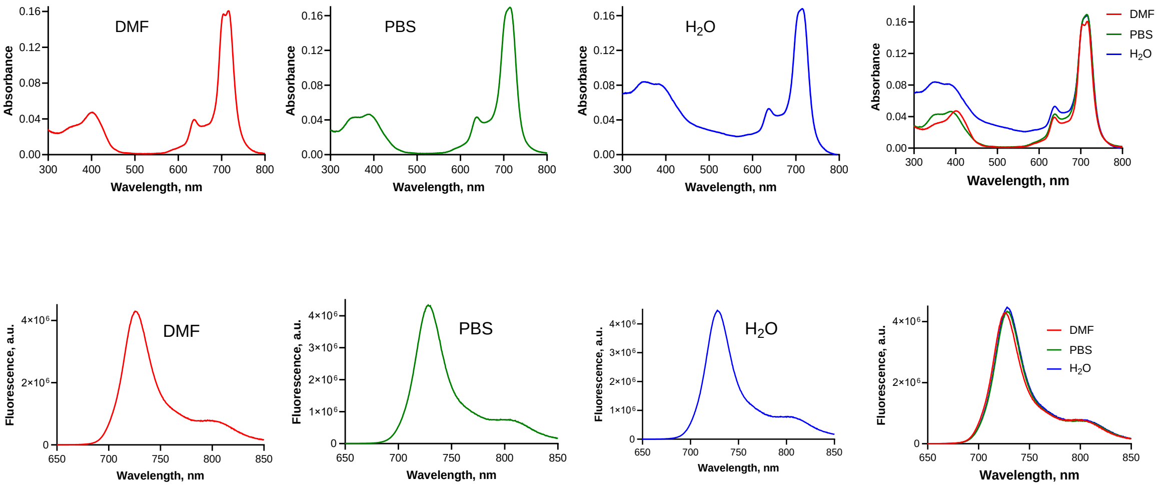

Fig. S23: Absorption and fluorescence emission spectra of **C11Zn** (1µM) in DMF (red), PBS (green) and water (blue).

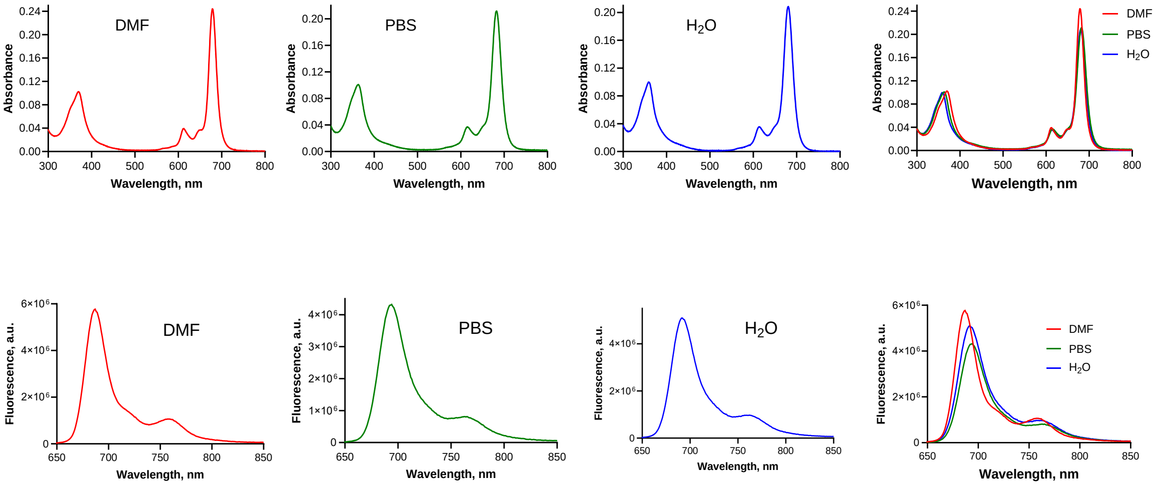

Fig. S24: Absorption and fluorescence emission spectra of **C12Zn** (1µM) in DMF (red), PBS (green) and water (blue).

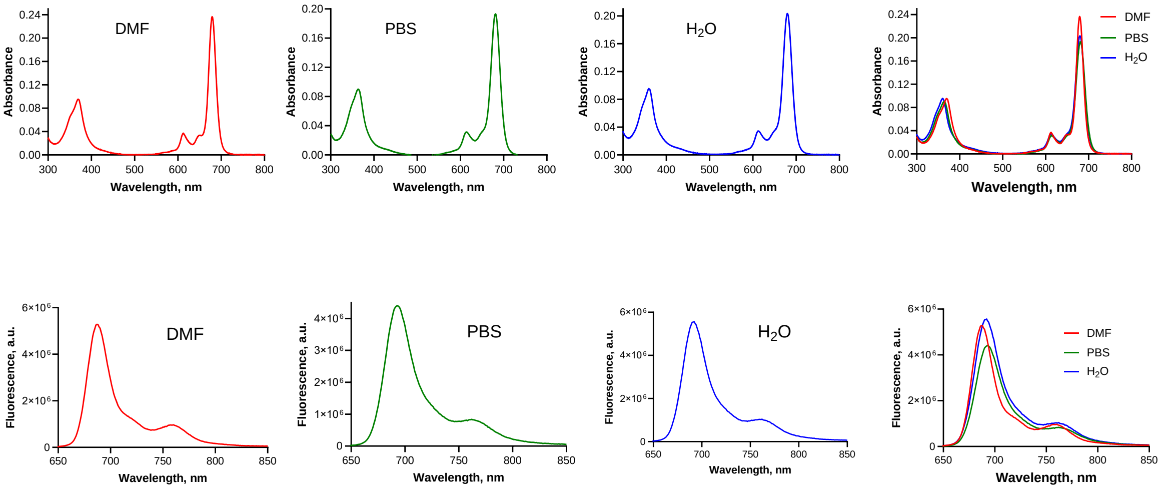

Fig. S25: Absorption and fluorescence emission spectra of **C13Zn** (1µM) in DMF (red), PBS (green) and water (blue).

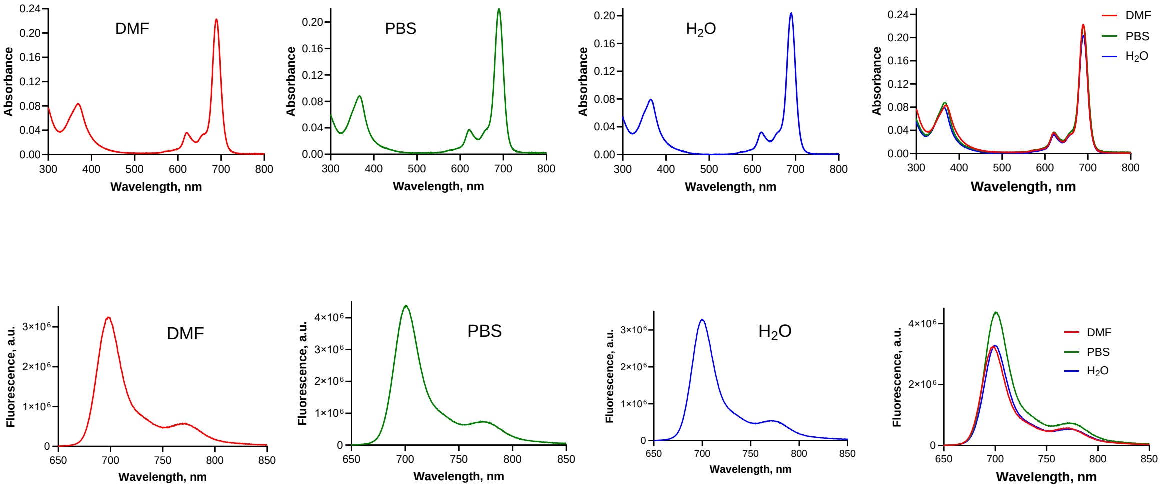

Fig. S26: Absorption and fluorescence emission spectra of **C14Zn** (1µM) in DMF (red), PBS (green) and water (blue).

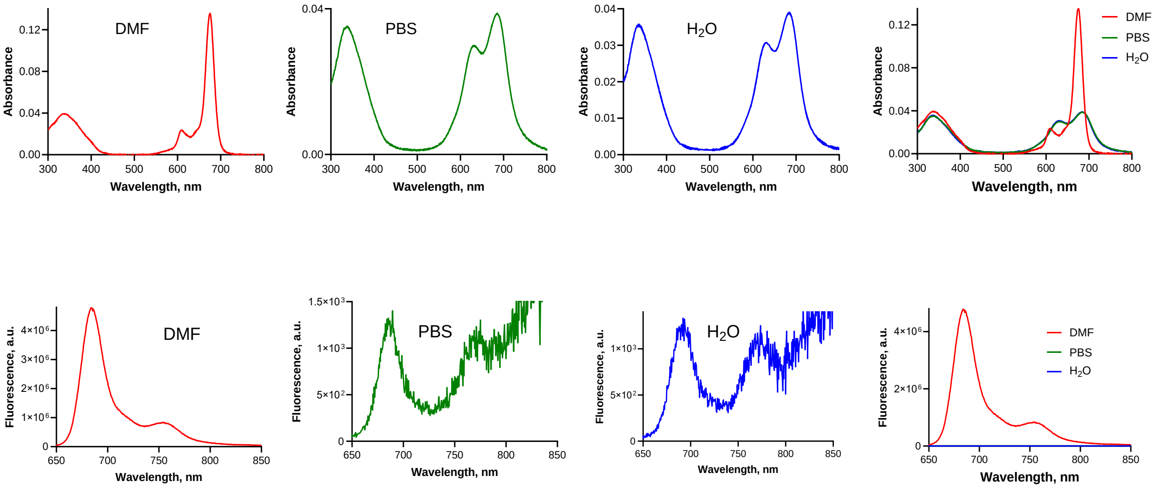

Fig. S27: Absorption and fluorescence emission spectra of **C15Zn** (1µM) in DMF (red), PBS (green) and water (blue).

Neutral derivatives

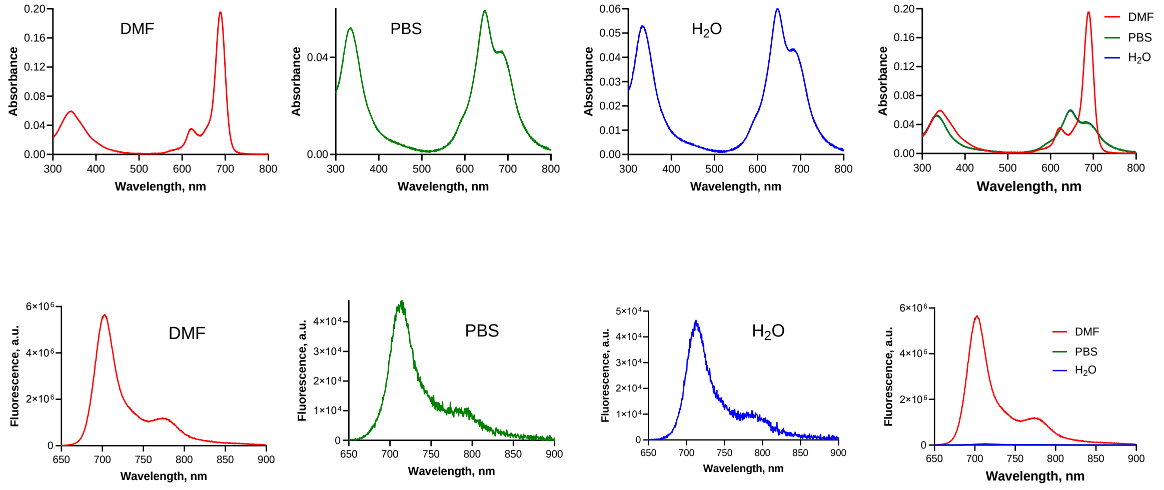

Fig. S28: Absorption and fluorescence emission spectra of **N1Zn** (1µM) in DMF (red), PBS (green) and water (blue).

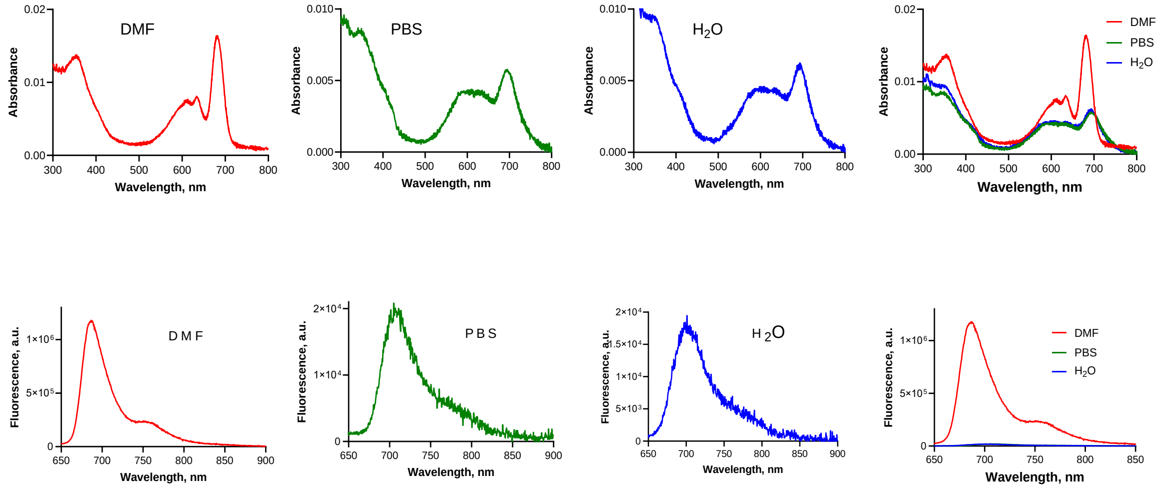

Fig. S29: Absorption and fluorescence emission spectra of **N2Mg** (1µM) in DMF (red), PBS (green) and water (blue).

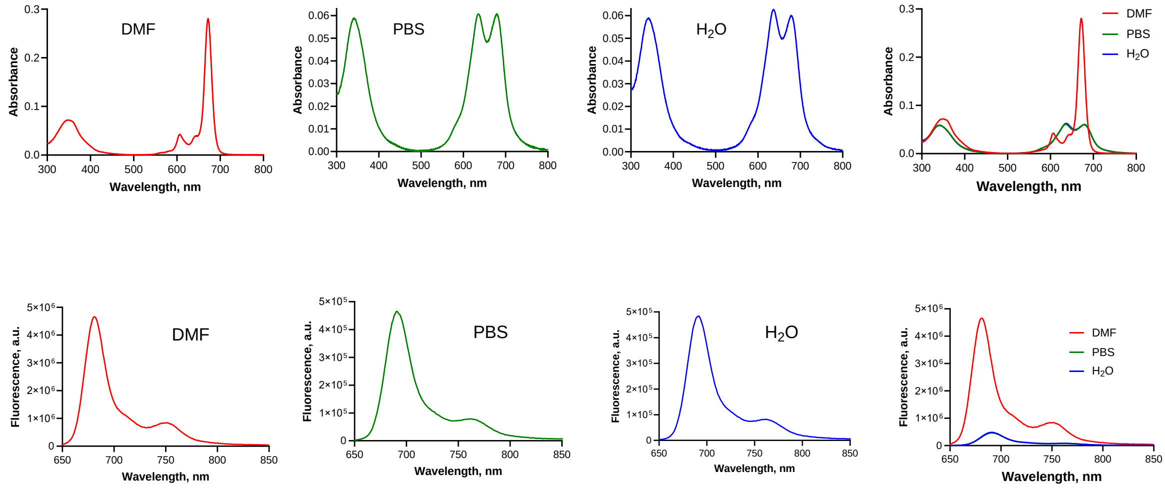

Fig. S30: Absorption and fluorescence emission spectra of **N3Zn** (1µM) in DMF (red), PBS (green) and water (blue).

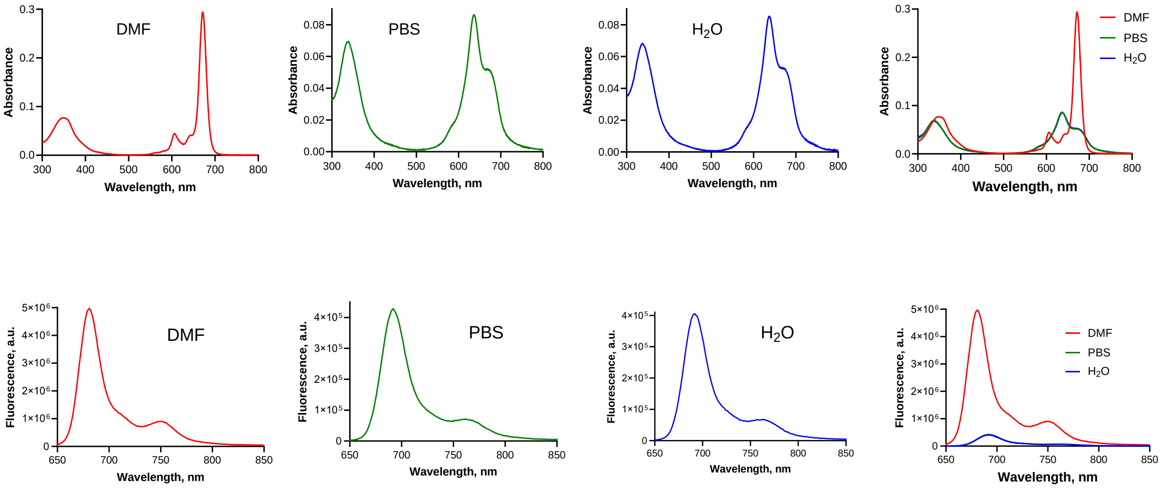

Fig. S31: Absorption and fluorescence emission spectra of **N4Zn** (1µM) in DMF (red), PBS (green) and water (blue).

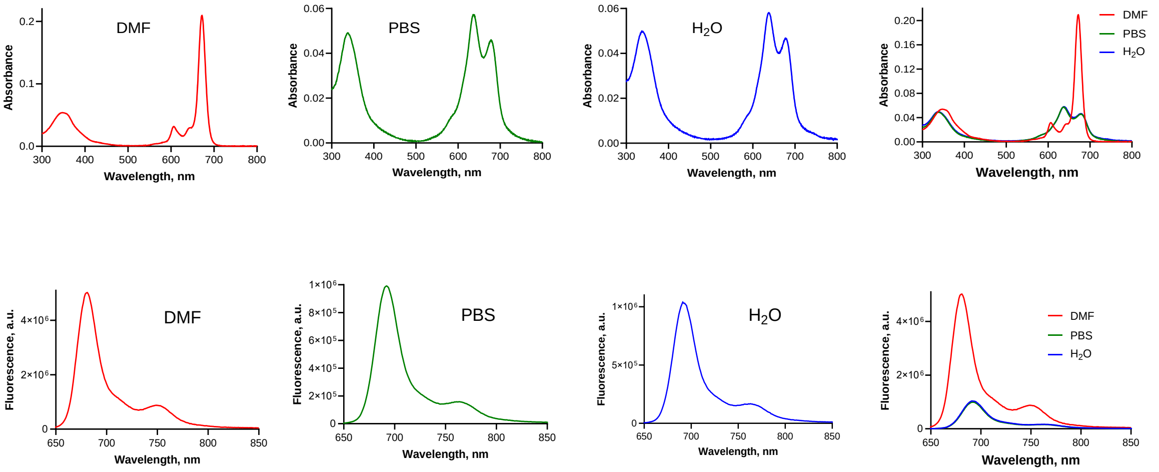

Fig. S32: Absorption and fluorescence emission spectra of **N5Zn** (1µM) in DMF (red), PBS (green) and water (blue).

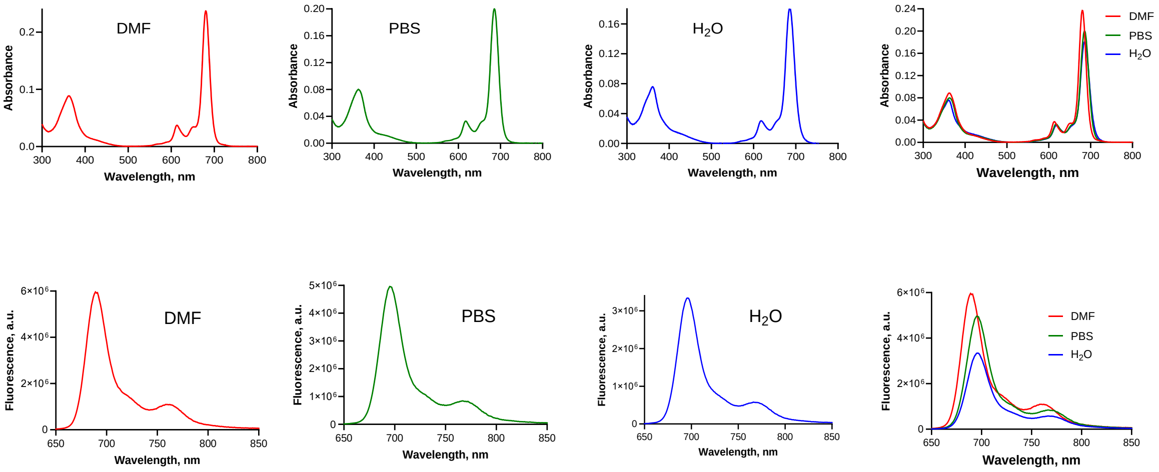

Fig. S33: Absorption and fluorescence emission spectra of **N6Zn** (1µM) in DMF (red), PBS (green) and water (blue).

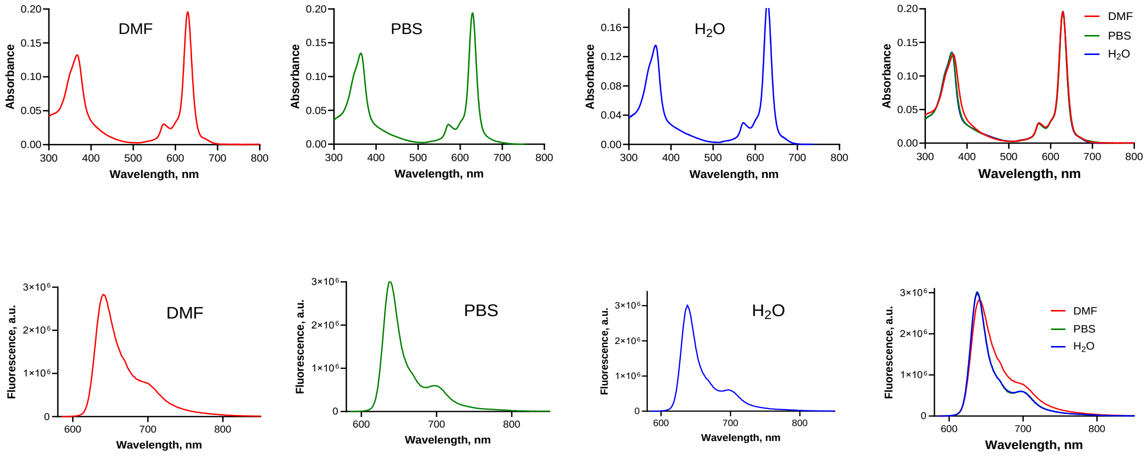

Fig. S34: Absorption and fluorescence emission spectra of **N7Zn** (1µM) in DMF (red), PBS (green) and water (blue).

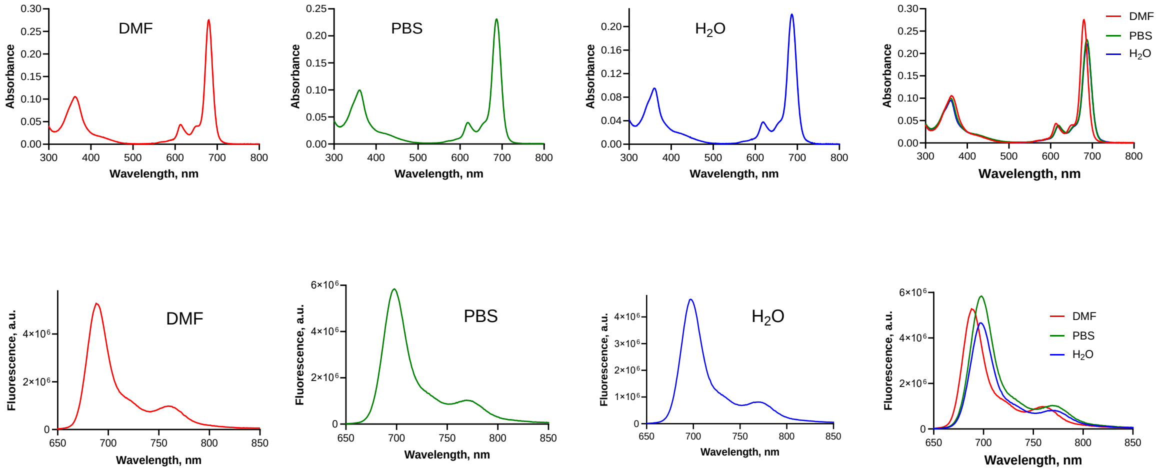

Fig. S35: Absorption and fluorescence emission spectra of **N8Zn** (1µM) in DMF (red), PBS (green) and water (blue).

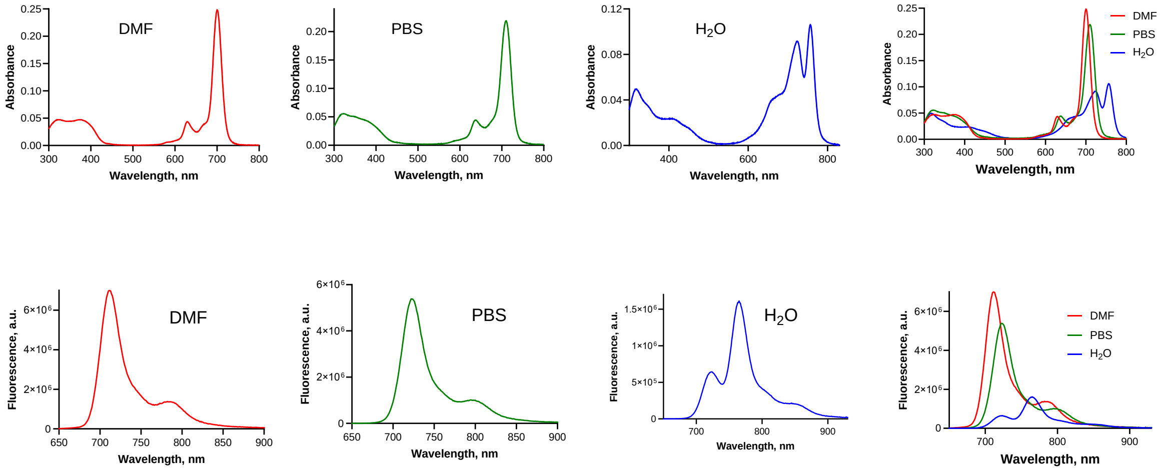

Fig. S36: Absorption and fluorescence emission spectra of **N9Zn** (1µM) in DMF (red), PBS (green) and water (blue).

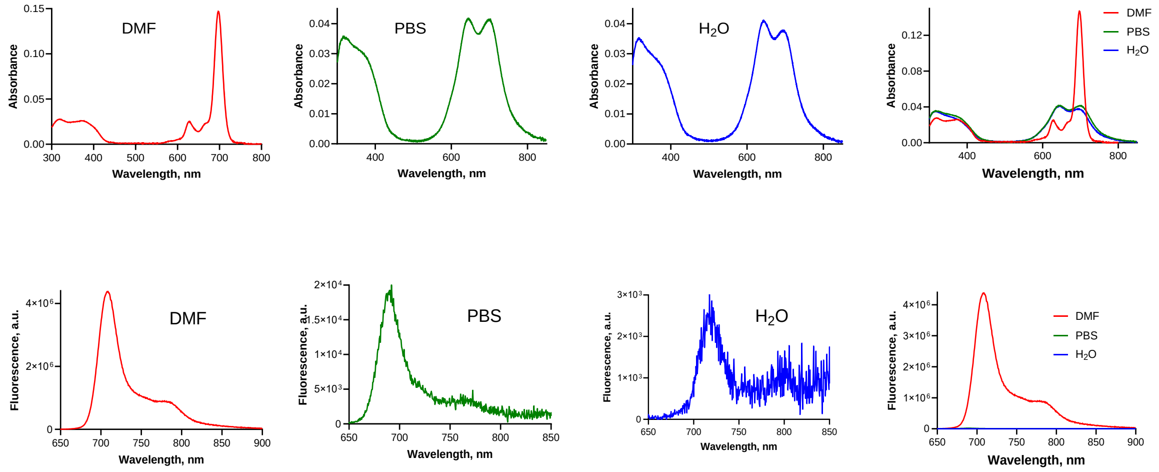

Fig. S37: Absorption and fluorescence emission spectra of **N10Zn** (1µM) in DMF (red), PBS (green) and water (blue).

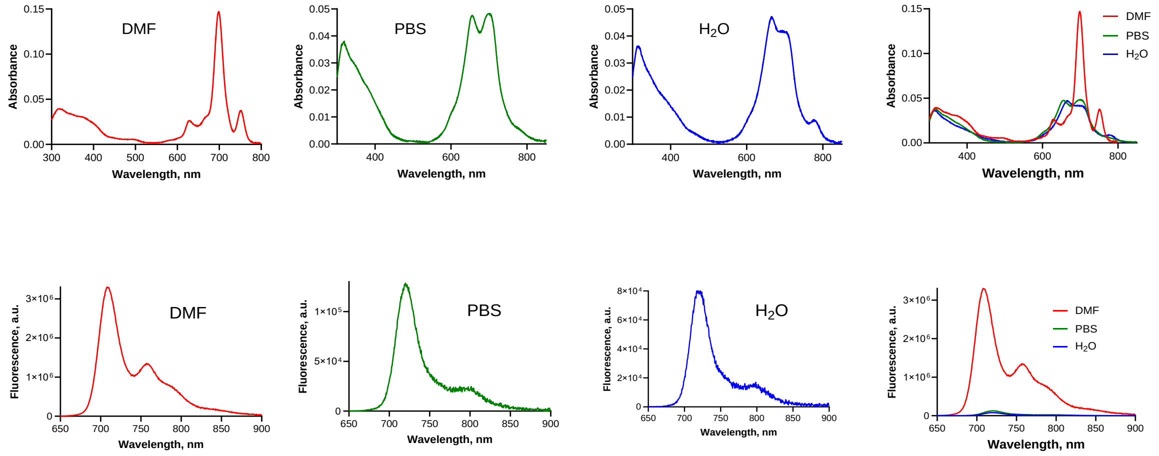

Fig. S38: Absorption and fluorescence emission spectra of **N11Zn** (1µM) in DMF (red), PBS (green) and water (blue)

.

Silicon derivatives

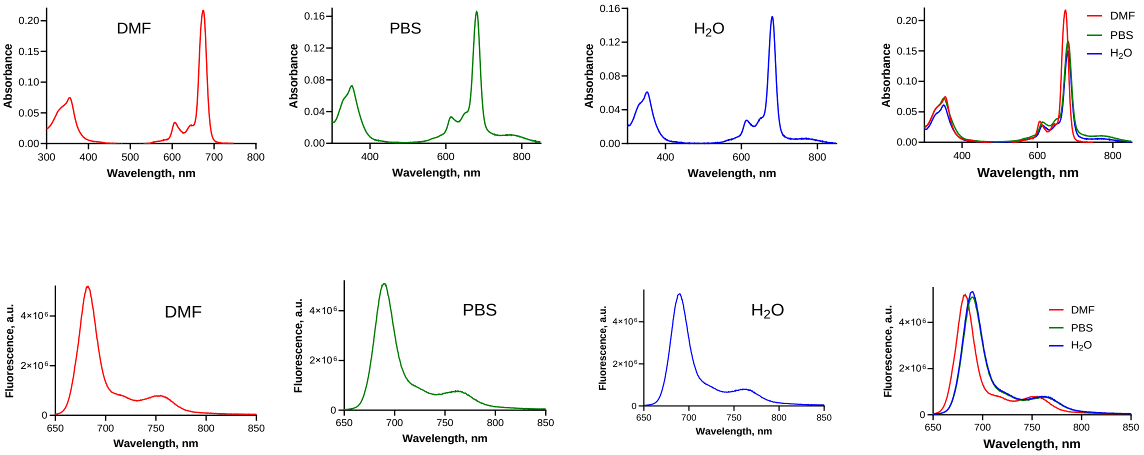

Fig. S39: Absorption and fluorescence emission spectra of **Si1-C** (1µM) in DMF (red), PBS (green) and water (blue).

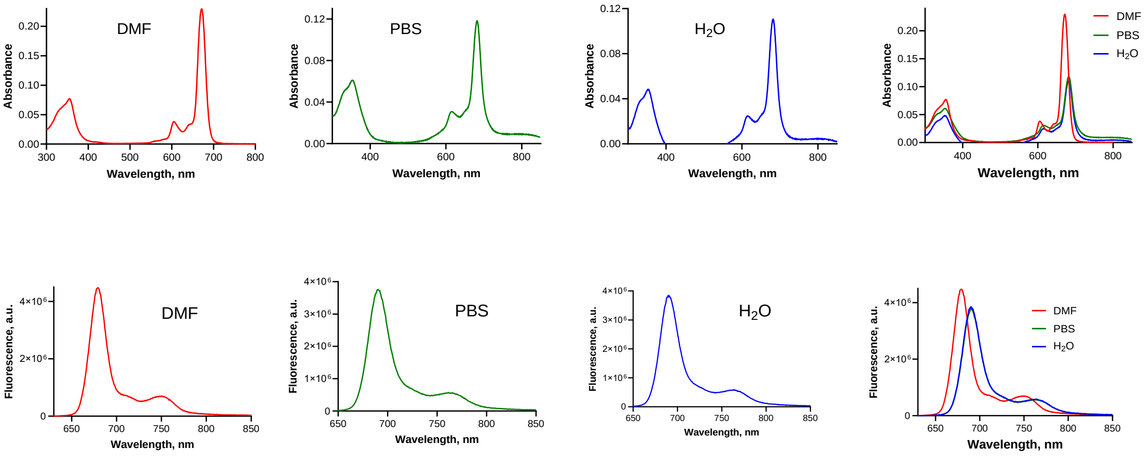

Fig. S40: Absorption and fluorescence emission spectra of **Si2-C** (1µM) in DMF (red), PBS (green) and water (blue).

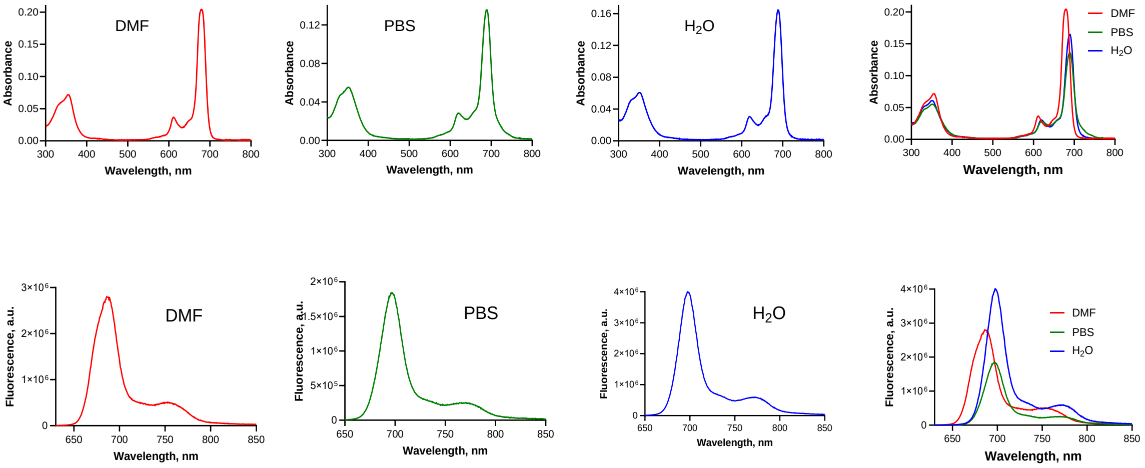

Fig. S41: Absorption and fluorescence emission spectra of **Si3-C** (1µM) in DMF (red), PBS (green) and water (blue).

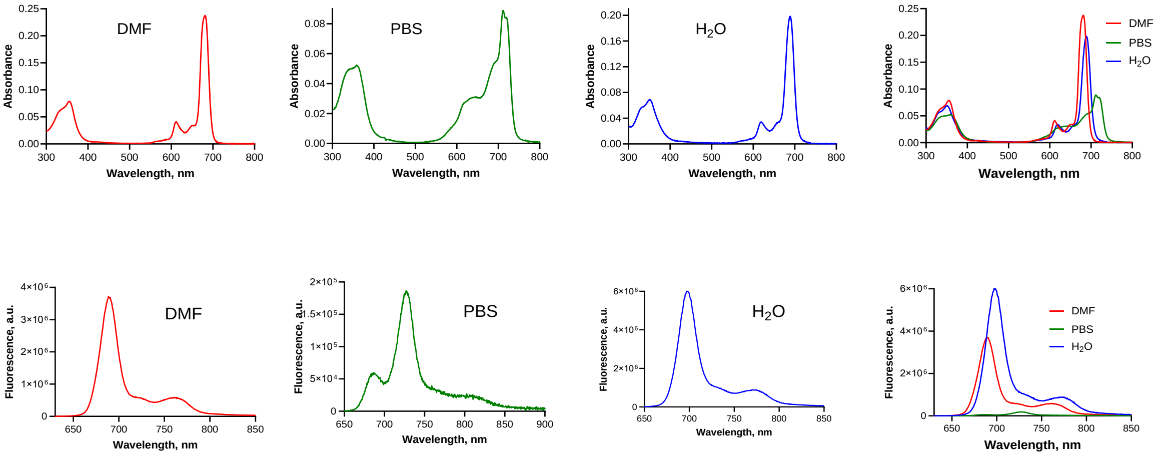

Fig. S42: Absorption and fluorescence emission spectra of **Si4-C** (1µM) in DMF (red), PBS (green) and water (blue).

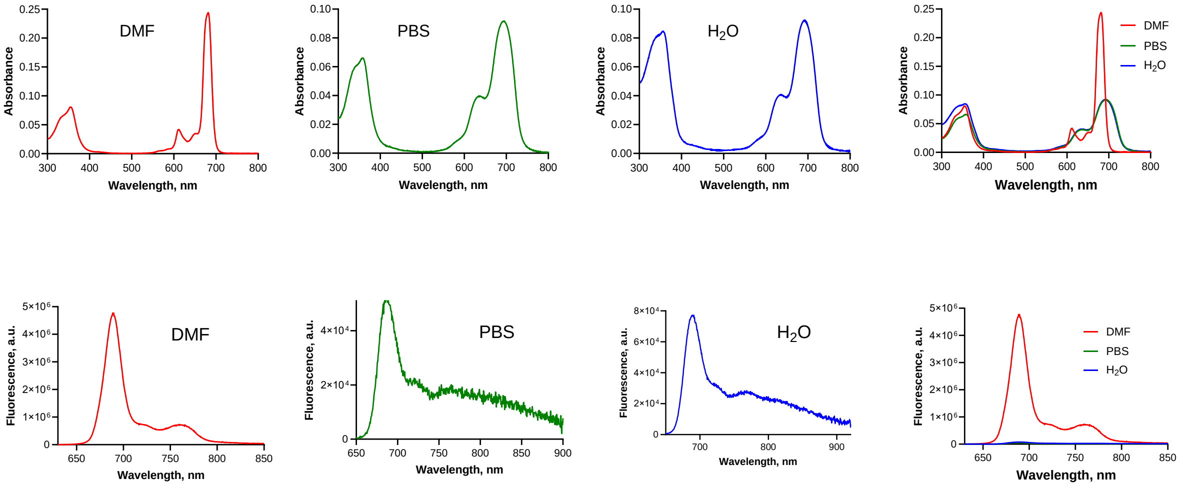

Fig. S43: Absorption and fluorescence emission spectra of **Si5-N** (1µM) in DMF (red), PBS (green) and water (blue).

Comparison of absorption spectra of **C5Zn** with different counter anions

a)

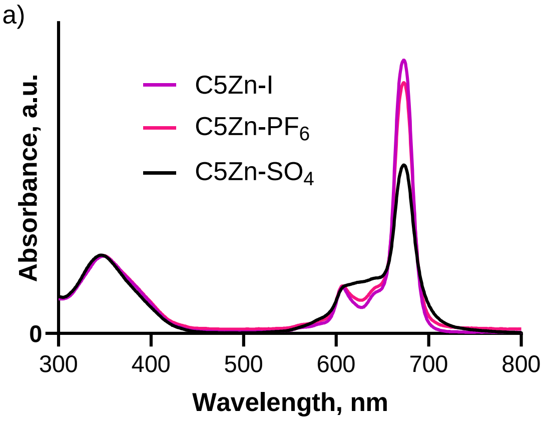

Fig. S44: Comparison of absorption spectra of **C5Zn-I**, **C5Zn-PF$_6$** and **C5Zn-SO$_4$** (in DMF, c = 1µM). Spectra are normalized at B band.

Interaction with bovine-serum albumin (BSA)

Anionic derivatives

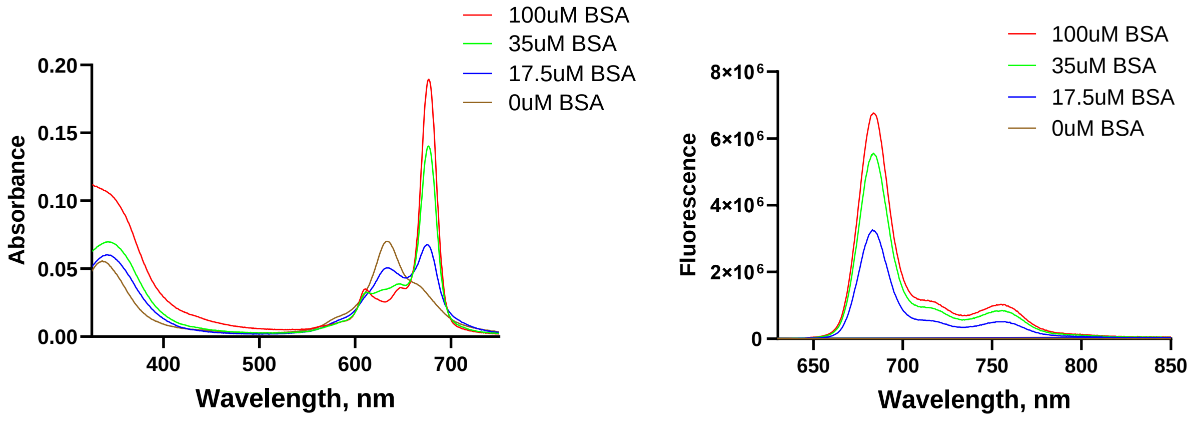

Fig. S45: Changes in absorption and fluorescence emission spectra of **A1Zn** (1µM) upon addition of bovine-serum albumin (BSA).

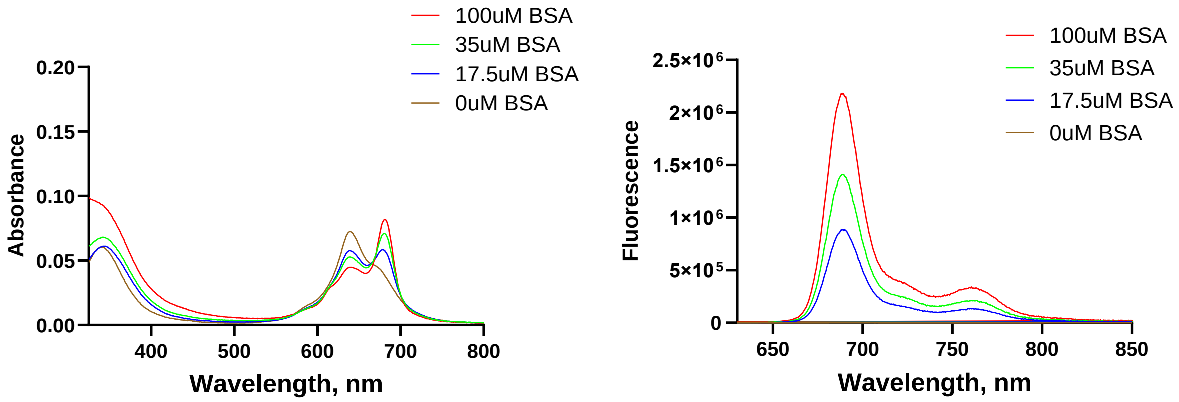

Fig. S46: Changes in absorption and fluorescence emission spectra of **A2Zn** (1µM) upon addition of bovine-serum albumin (BSA).

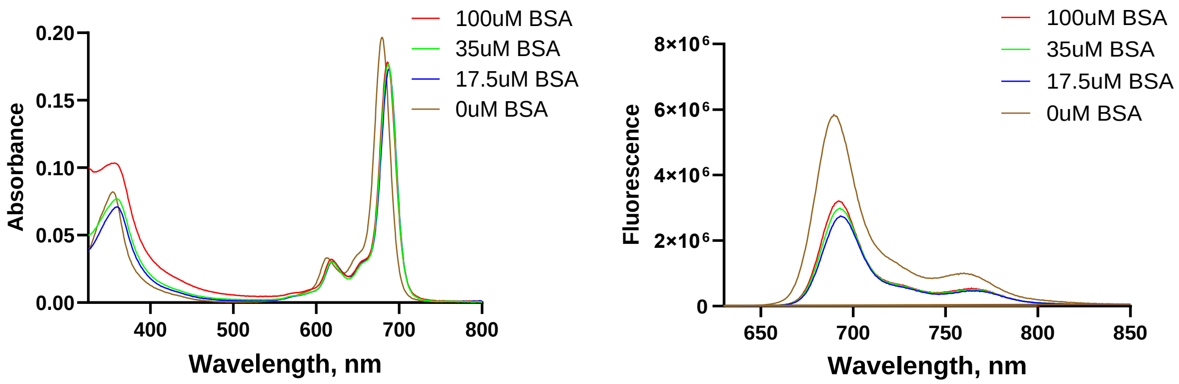

Fig. S47: Changes in absorption and fluorescence emission spectra of **A3Zn** (1µM) upon addition of bovine-serum albumin (BSA).

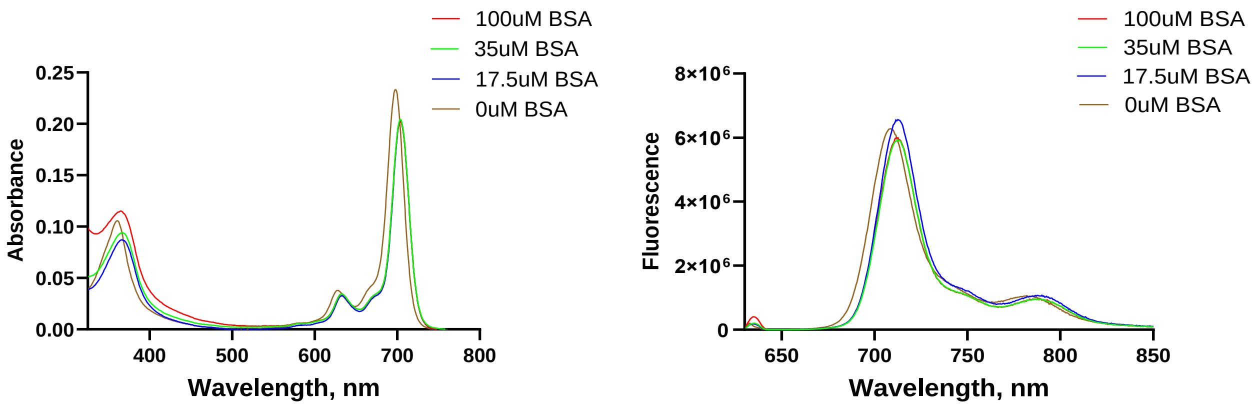

Fig. S48: Changes in absorption and fluorescence emission spectra of **A4Zn** (1µM) upon addition of bovine-serum albumin (BSA).

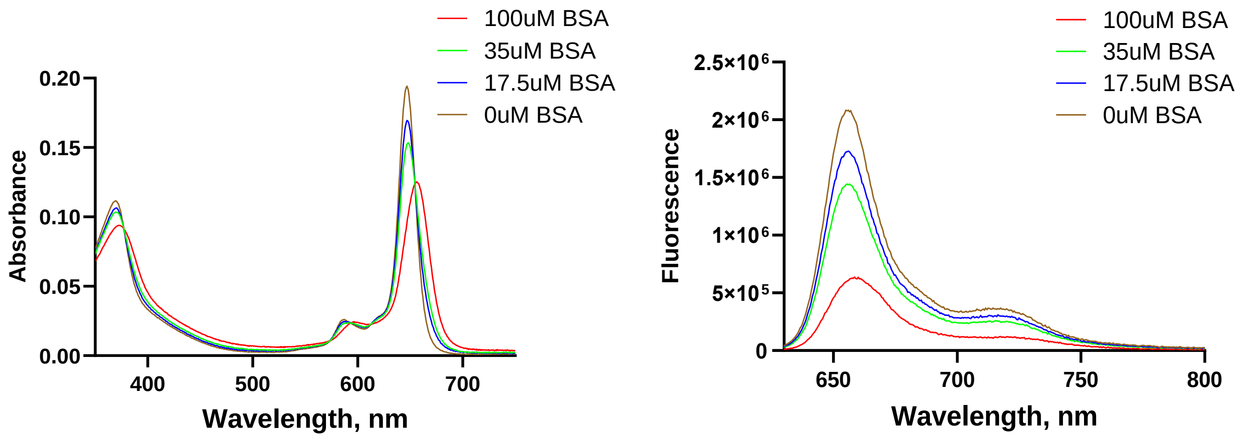

Fig. S49: Changes in absorption and fluorescence emission spectra of **A5Zn** (1µM) upon addition of bovine-serum albumin (BSA).

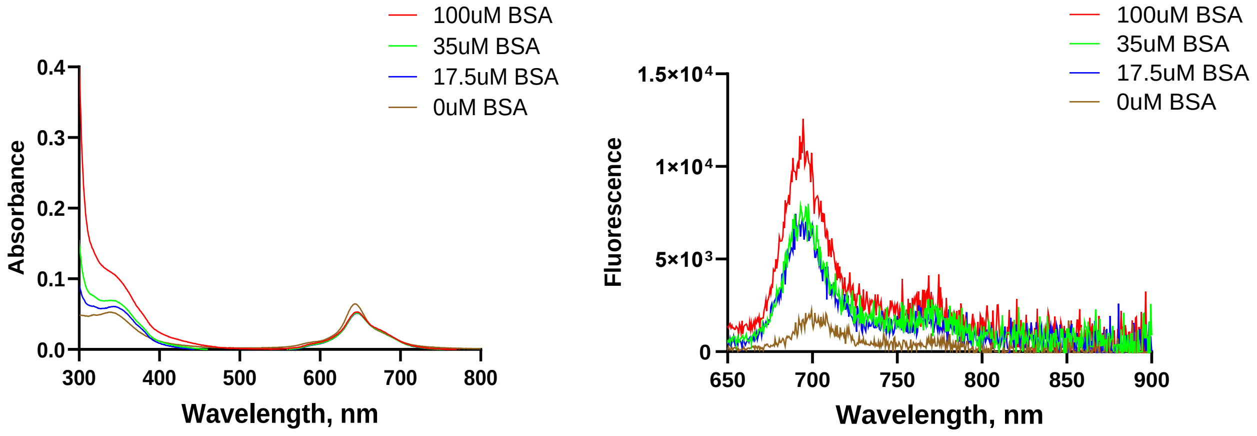

Fig. S50: Changes in absorption and fluorescence emission spectra of **A6Zn** (1µM) upon addition of bovine-serum albumin (BSA).

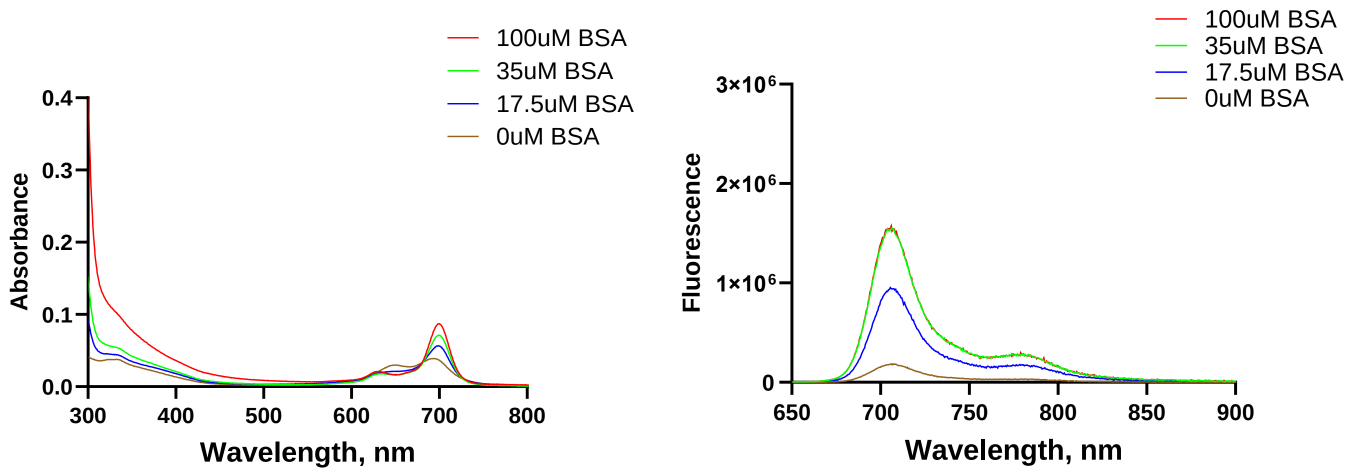

Fig. S51: Changes in absorption and fluorescence emission spectra of **A7Zn** (1µM) upon addition of bovine-serum albumin (BSA).

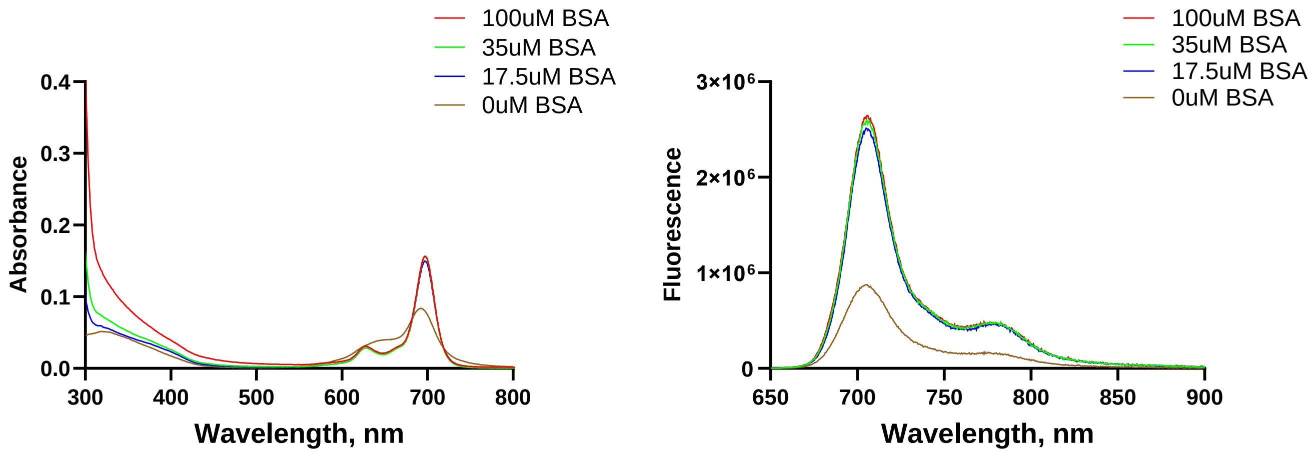

Fig. S52: Changes in absorption and fluorescence emission spectra of **A8Zn** (1µM) upon addition of bovine-serum albumin (BSA).

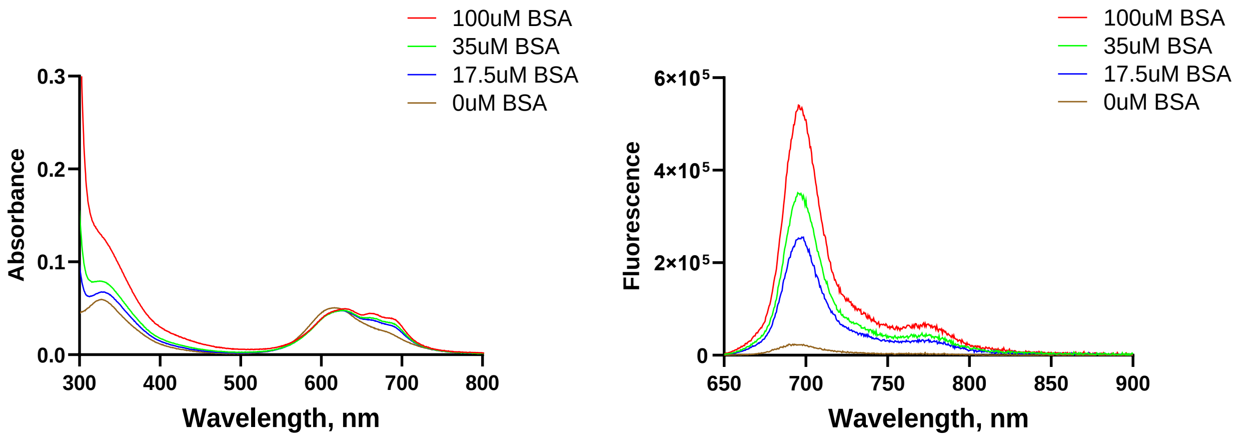

Fig. S53: Changes in absorption and fluorescence emission spectra of **A9H** (1µM) upon addition of bovine-serum albumin (BSA).

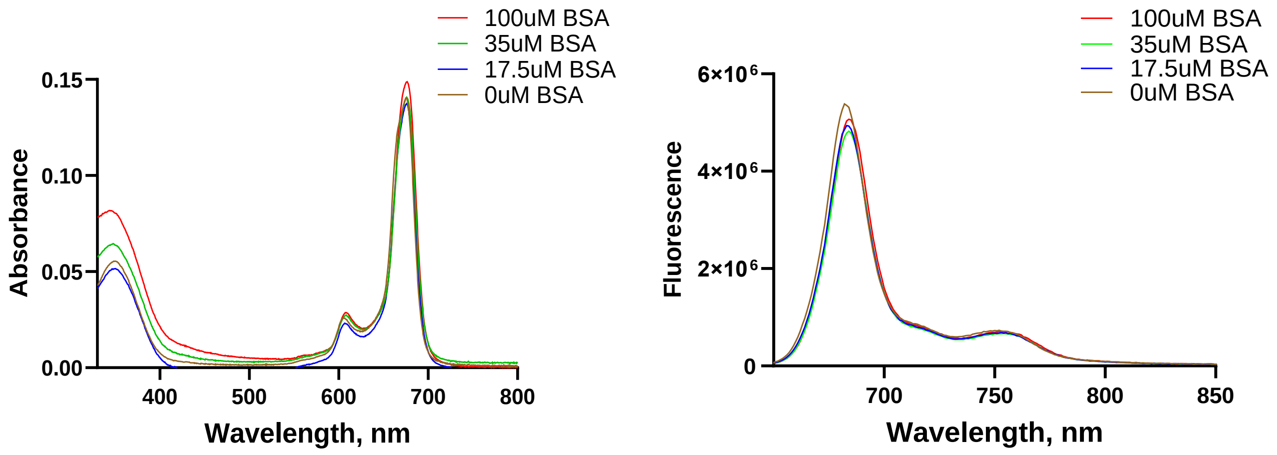

Fig. S54: Changes in absorption and fluorescence emission spectra of **A10Al** (1µM) upon addition of bovine-serum albumin (BSA).

Cationic derivatives

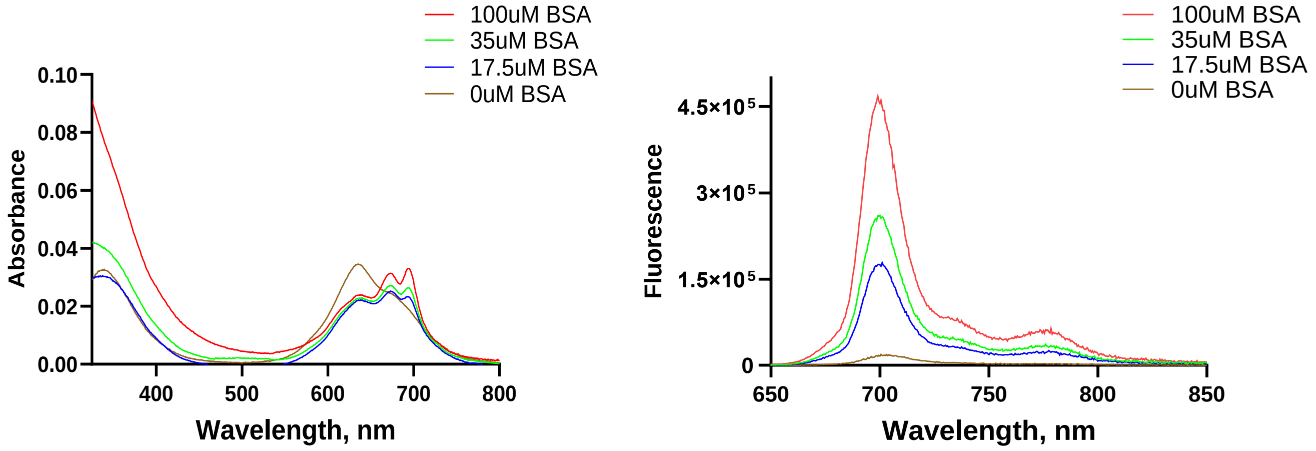

Fig. S55: Changes in absorption and fluorescence emission spectra of **C1Zn** (1µM) upon addition of bovine-serum albumin (BSA).

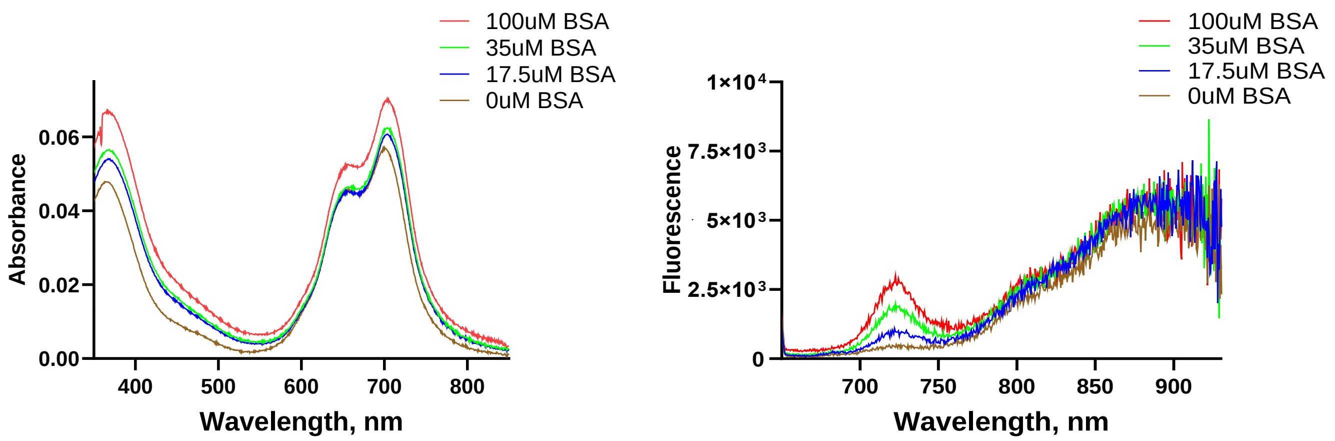

Fig. S56: Changes in absorption and fluorescence emission spectra of **C2Zn** (1µM) upon addition of bovine-serum albumin (BSA).

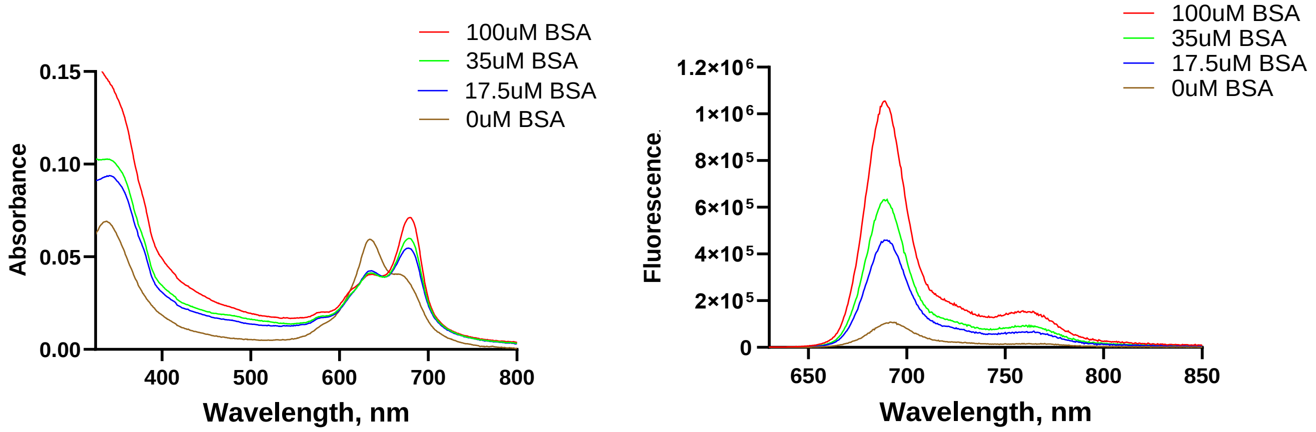

Fig. S57: Changes in absorption and fluorescence emission spectra of **C3Zn** (1µM) upon addition of bovine-serum albumin (BSA).

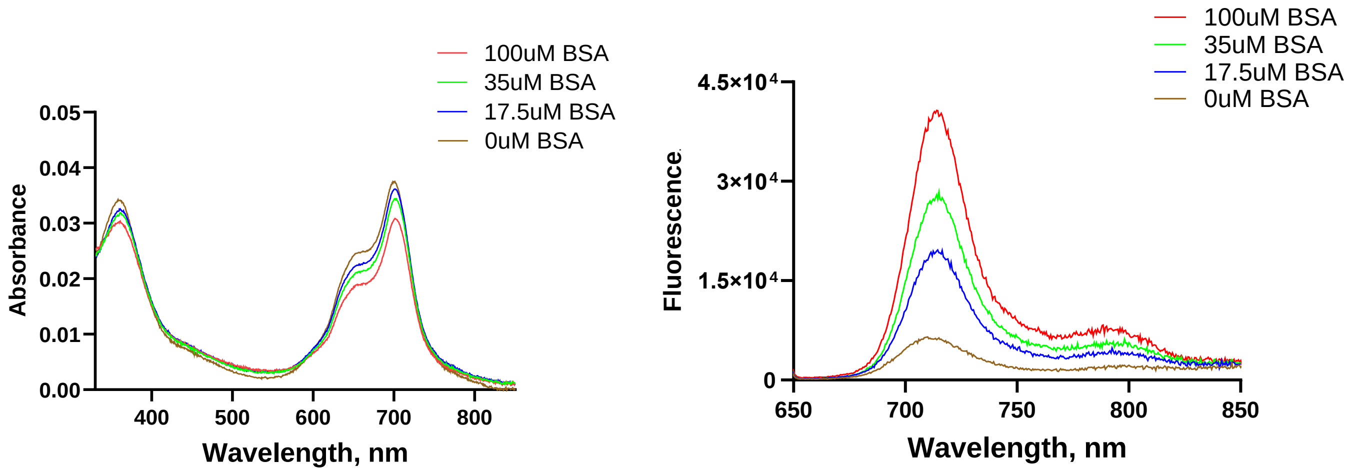

Fig. S58: Changes in absorption and fluorescence emission spectra of **C4Zn** (1µM) upon addition of bovine-serum albumin (BSA).

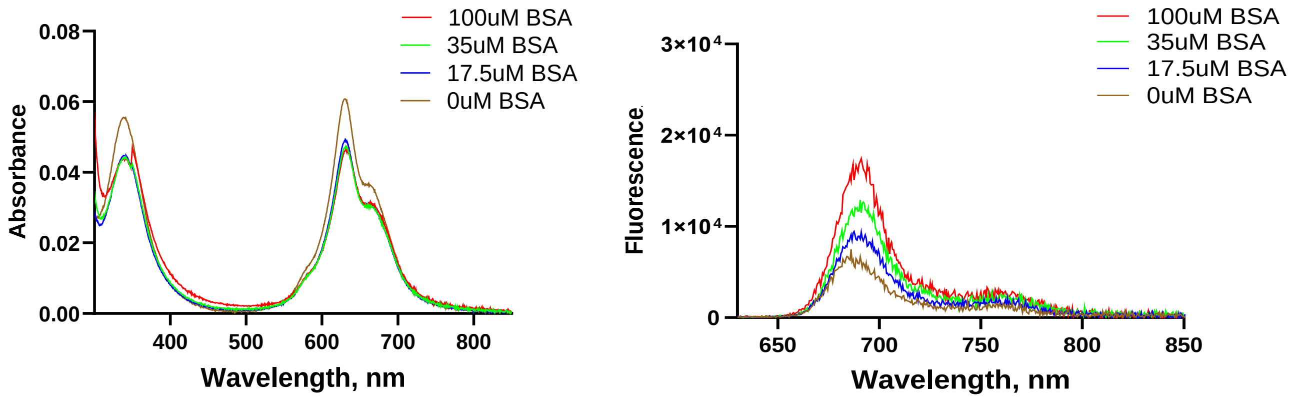

Fig. S59: Changes in absorption and fluorescence emission spectra of **C5Zn-I** (1μM) upon addition of bovine-serum albumin (BSA).

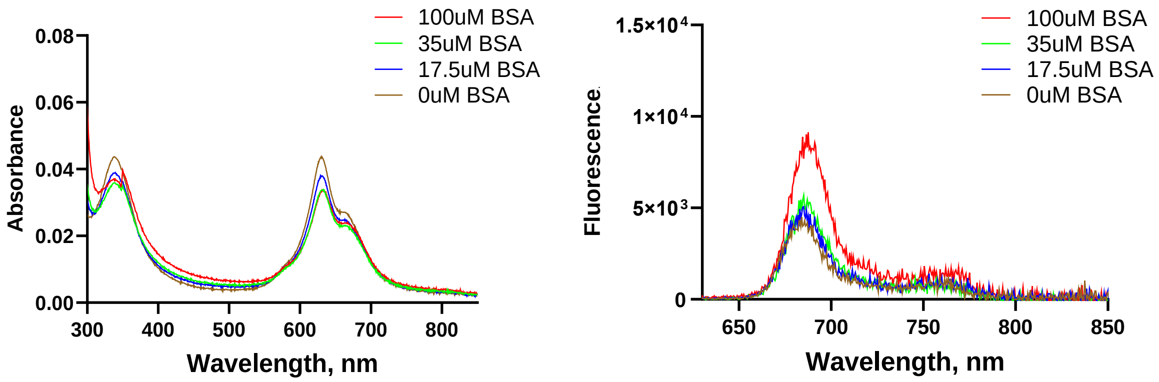

Fig. S60: Changes in absorption and fluorescence emission spectra of **C5Zn-PF$_6$** (1μM) upon addition of bovine-serum albumin (BSA).

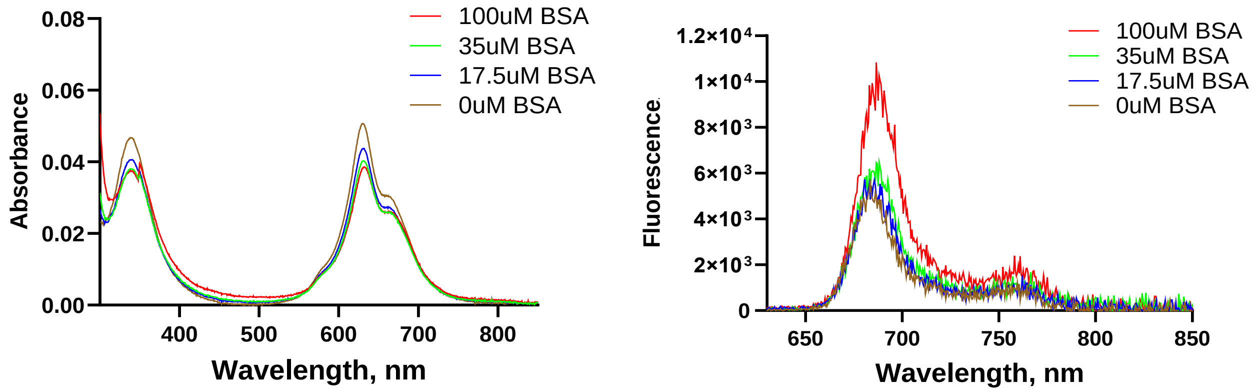

Fig. S61: Changes in absorption and fluorescence emission spectra of **C5Zn-SO$_4$** (1μM) upon addition of bovine-serum albumin (BSA).

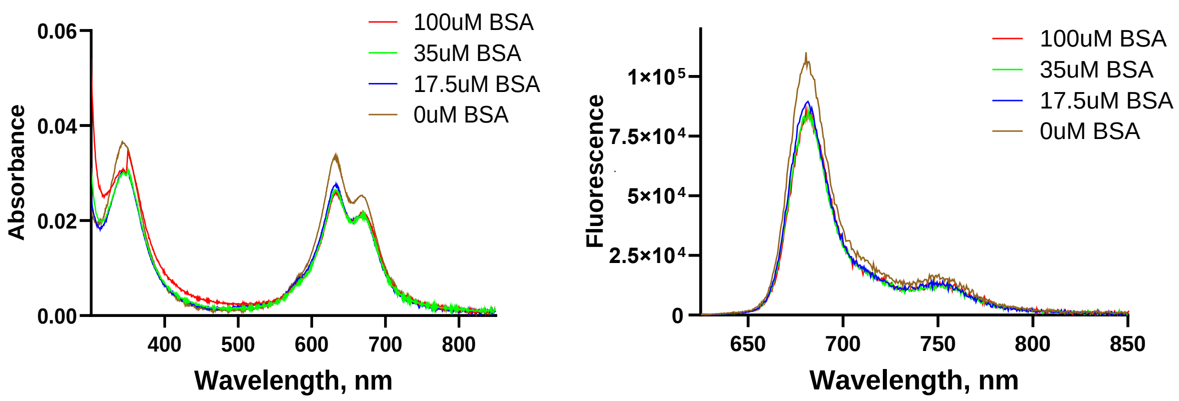

Fig. S62: Changes in absorption and fluorescence emission spectra of **C6Zn** (1µM) upon addition of bovine-serum albumin (BSA).

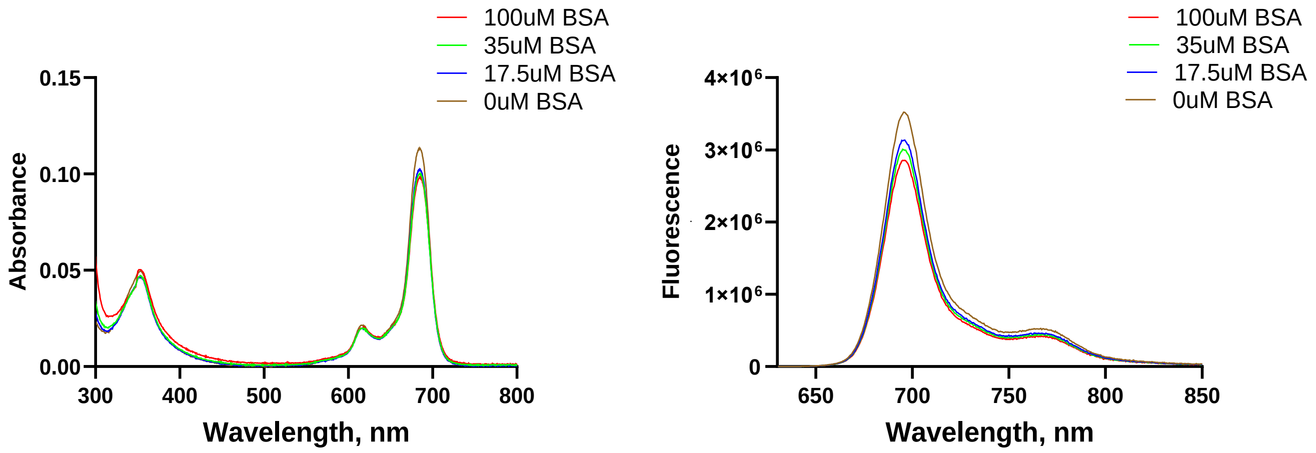

Fig. S63: Changes in absorption and fluorescence emission spectra of **C7Zn** (1µM) upon addition of bovine-serum albumin (BSA).

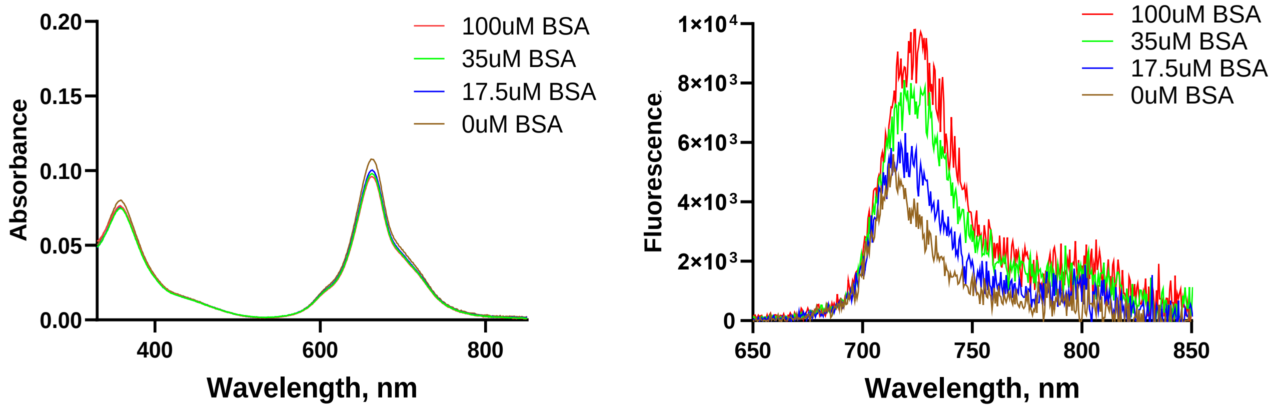

Fig. S64: Changes in absorption and fluorescence emission spectra of **C8Zn** (1µM) upon addition of bovine-serum albumin (BSA).

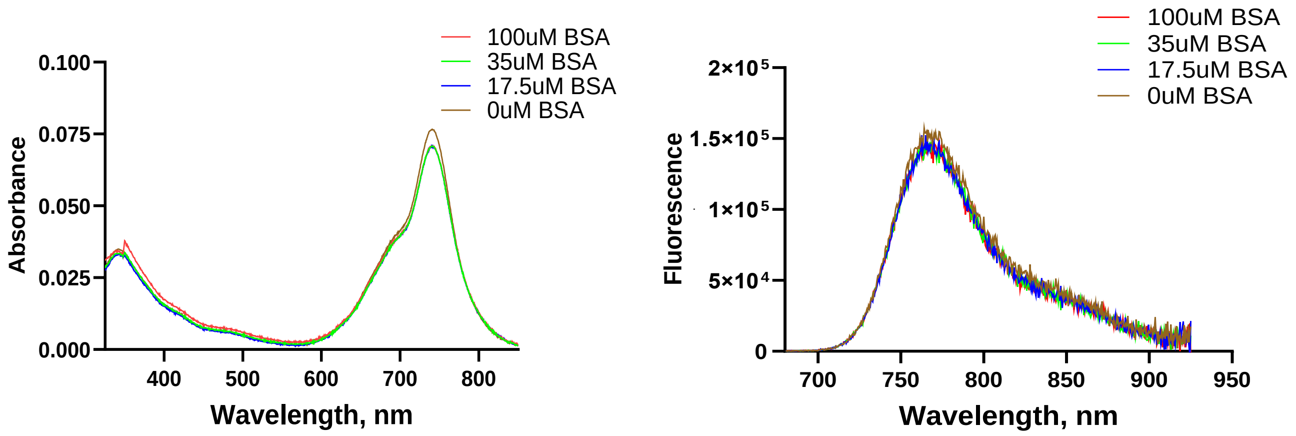

Fig. S65: Changes in absorption and fluorescence emission spectra of **C9Zn** (1μM) upon addition of bovine-serum albumin (BSA).

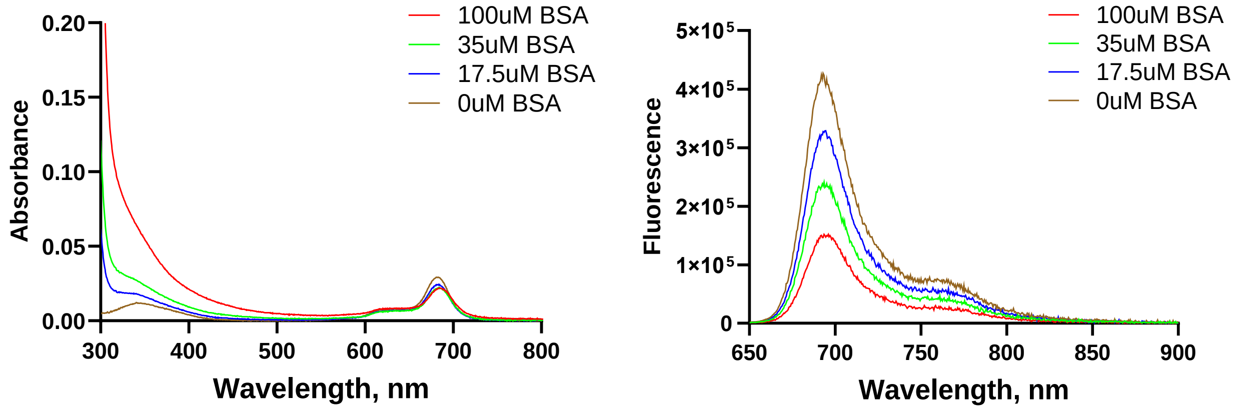

Fig. S66: Changes in absorption and fluorescence emission spectra of **C10Zn** (1μM) upon addition of bovine-serum albumin (BSA).

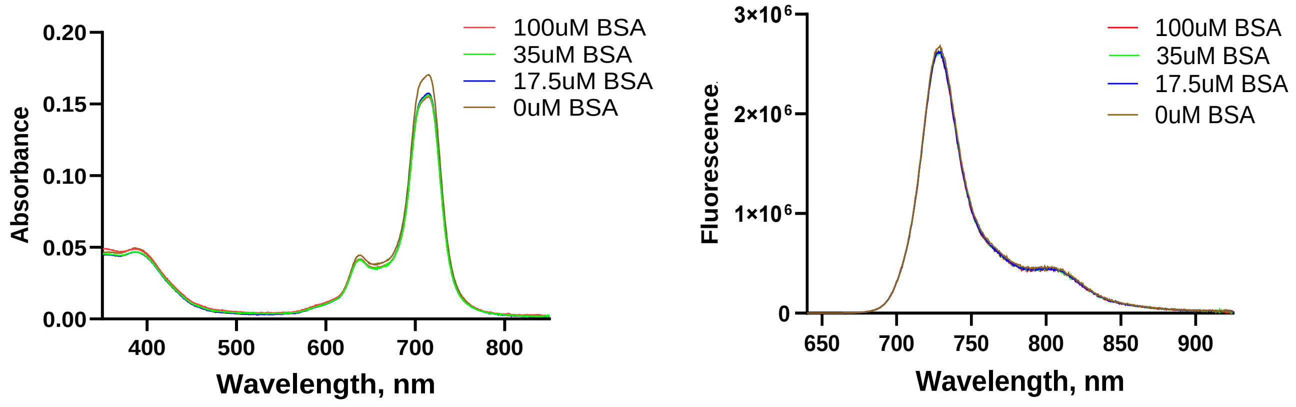

Fig. S67: Changes in absorption and fluorescence emission spectra of **C11Zn** (1μM) upon addition of bovine-serum albumin (BSA).

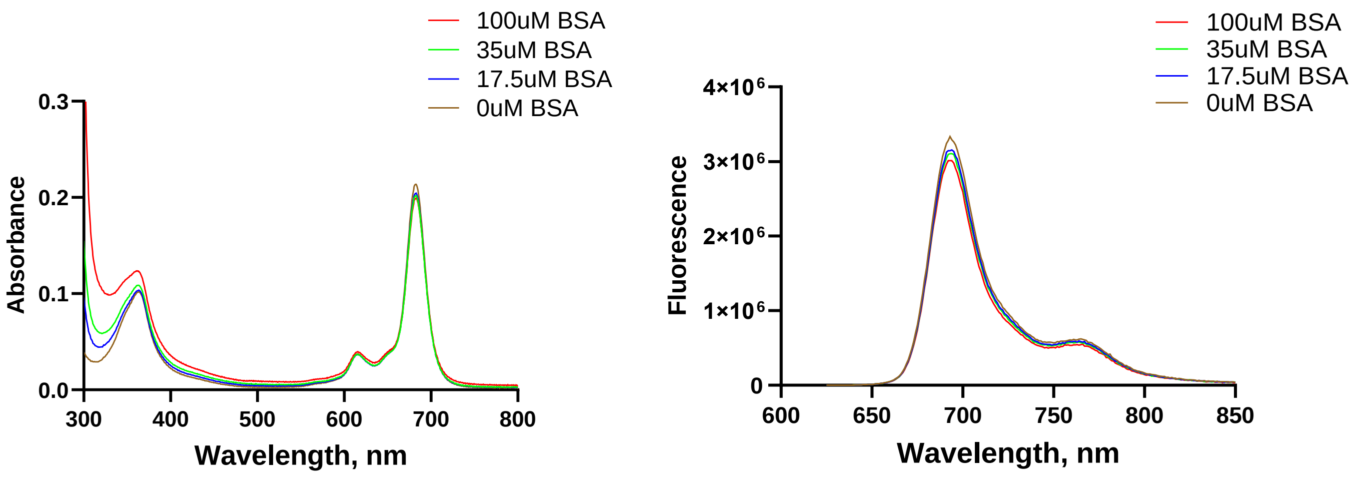

Fig. S68: Changes in absorption and fluorescence emission spectra of **C12Zn** (1μM) upon addition of bovine-serum albumin (BSA).

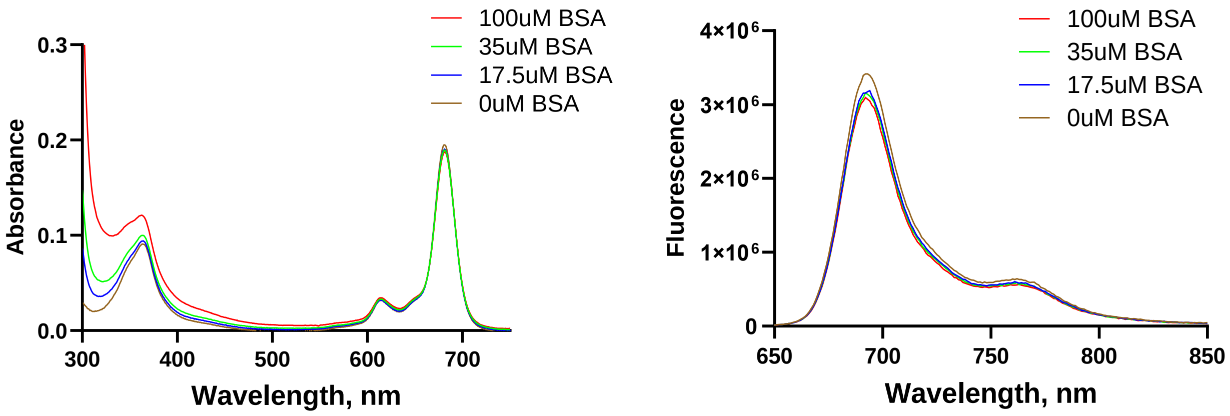

Fig. S69: Changes in absorption and fluorescence emission spectra of **C13Zn** (1μM) upon addition of bovine-serum albumin (BSA).

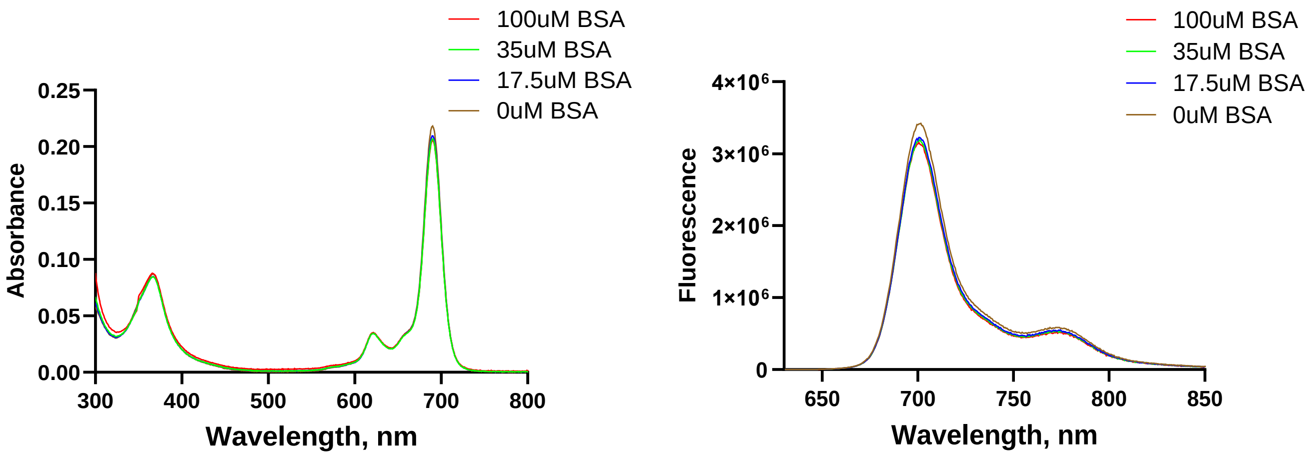

Fig. S70: Changes in absorption and fluorescence emission spectra of **C14Zn** (1μM) upon addition of bovine-serum albumin (BSA).

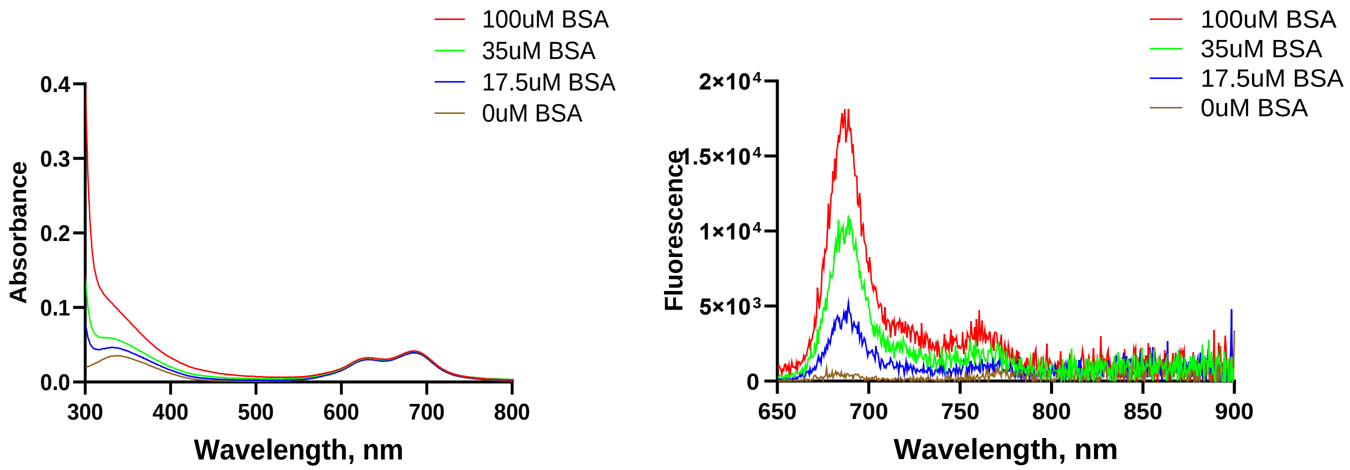

Fig. S71: Changes in absorption and fluorescence emission spectra of **C15Zn** (1µM) upon addition of bovine-serum albumin (BSA).

Neutral derivatives

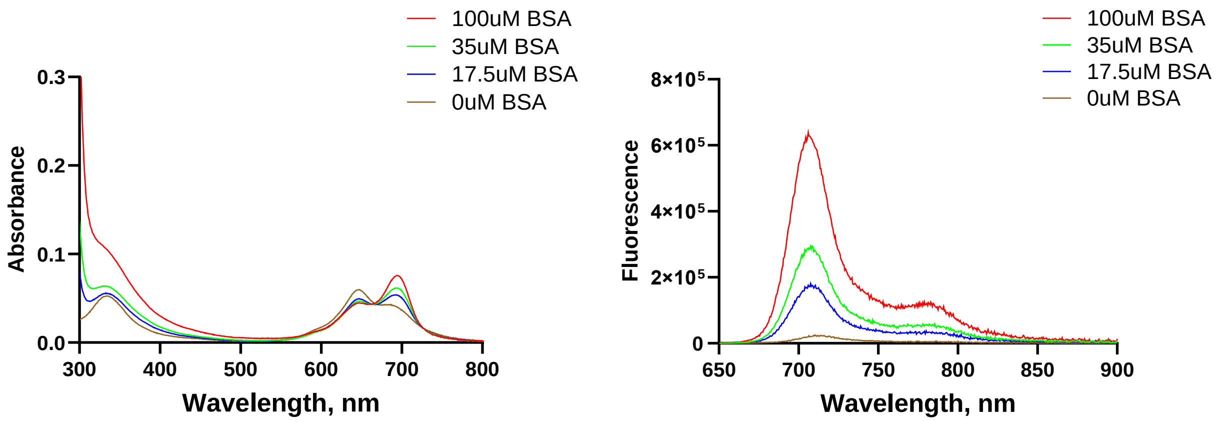

Fig. S72: Changes in absorption and fluorescence emission spectra of **N1Zn** (1µM) upon addition of bovine-serum albumin (BSA).

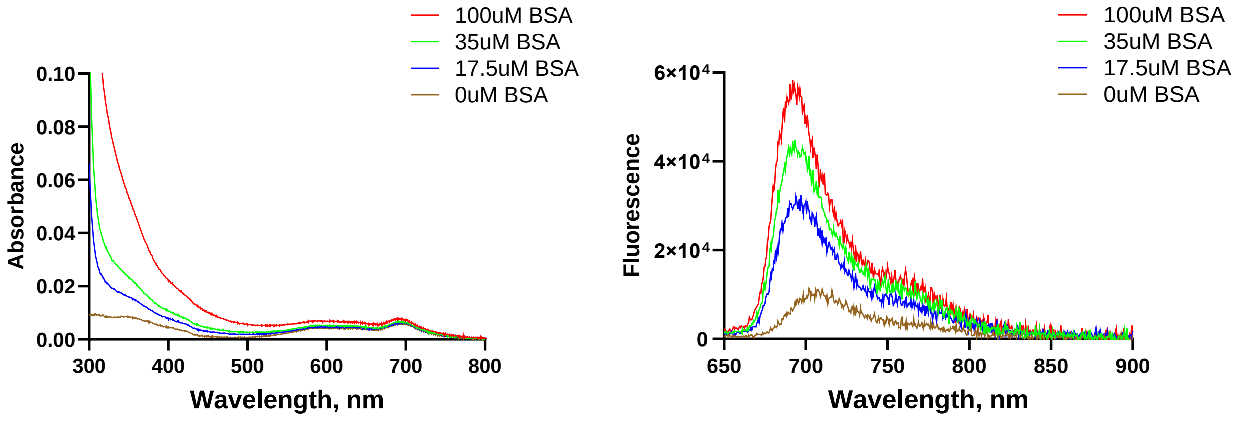

Fig. S73: Changes in absorption and fluorescence emission spectra of **N2Mg** (1µM) upon addition of bovine-serum albumin (BSA).

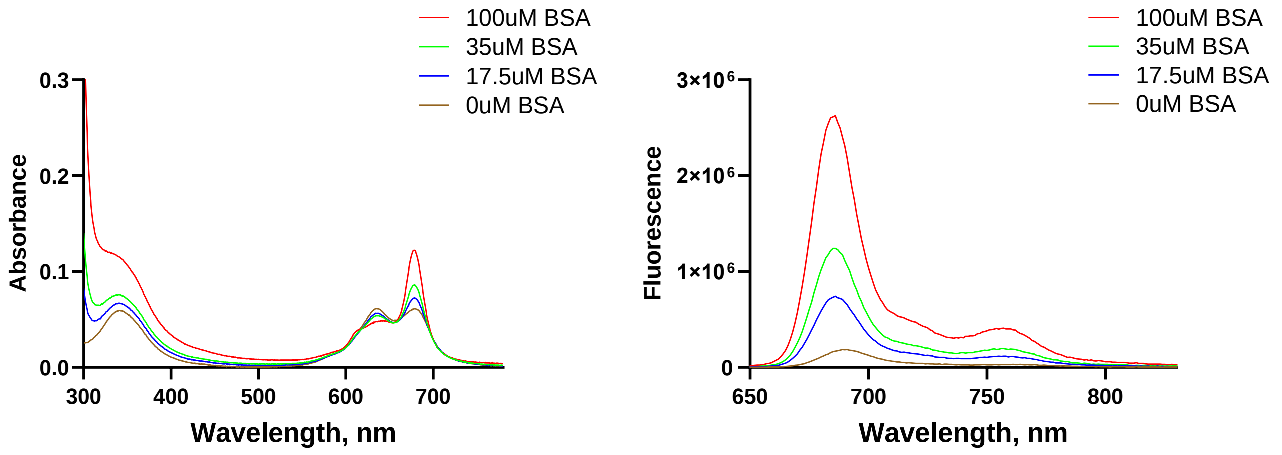

Fig. S74: Changes in absorption and fluorescence emission spectra of **N3Zn** (1μM) upon addition of bovine-serum albumin (BSA).

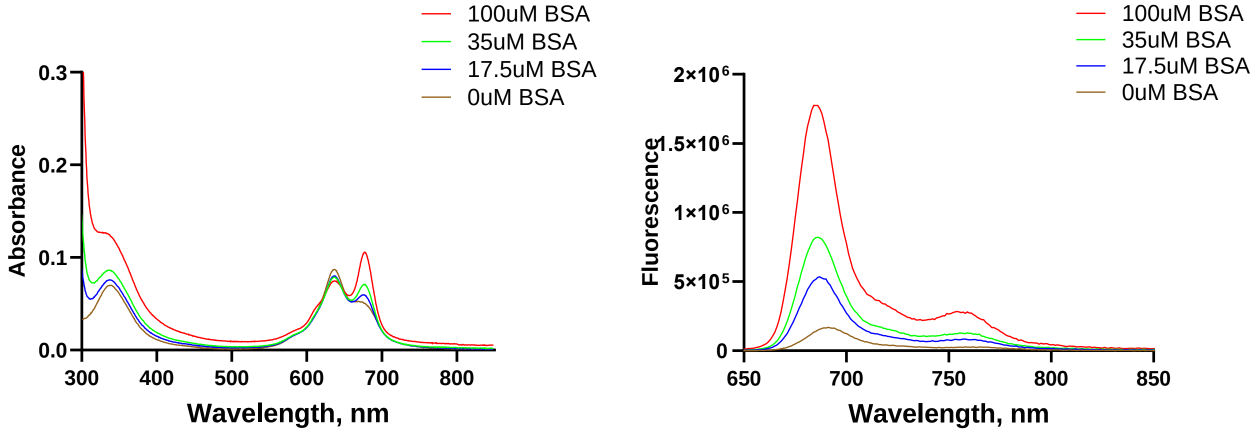

Fig. S75: Changes in absorption and fluorescence emission spectra of **N4Zn** (1μM) upon addition of bovine-serum albumin (BSA).

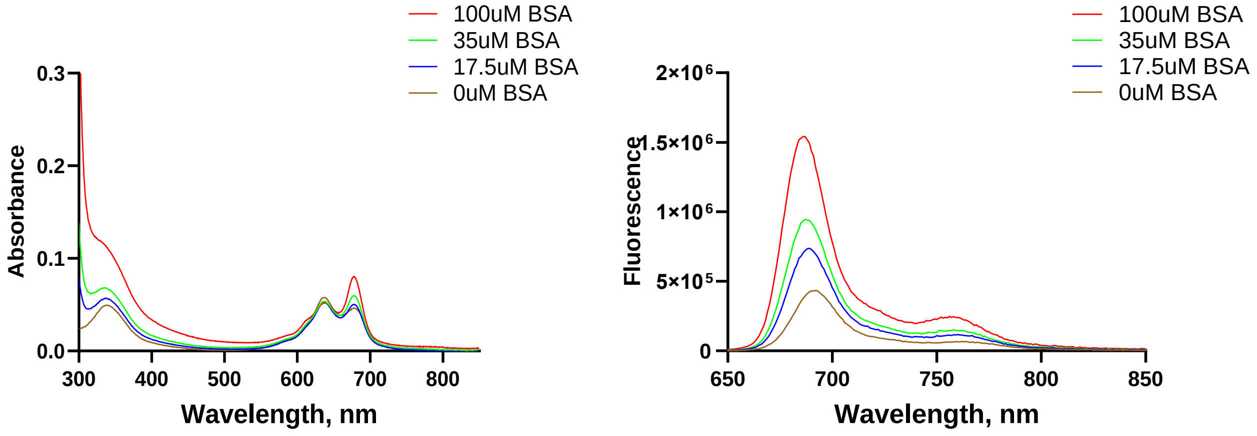

Fig. S76: Changes in absorption and fluorescence emission spectra of **N5n** (1μM) upon addition of bovine-serum albumin (BSA).

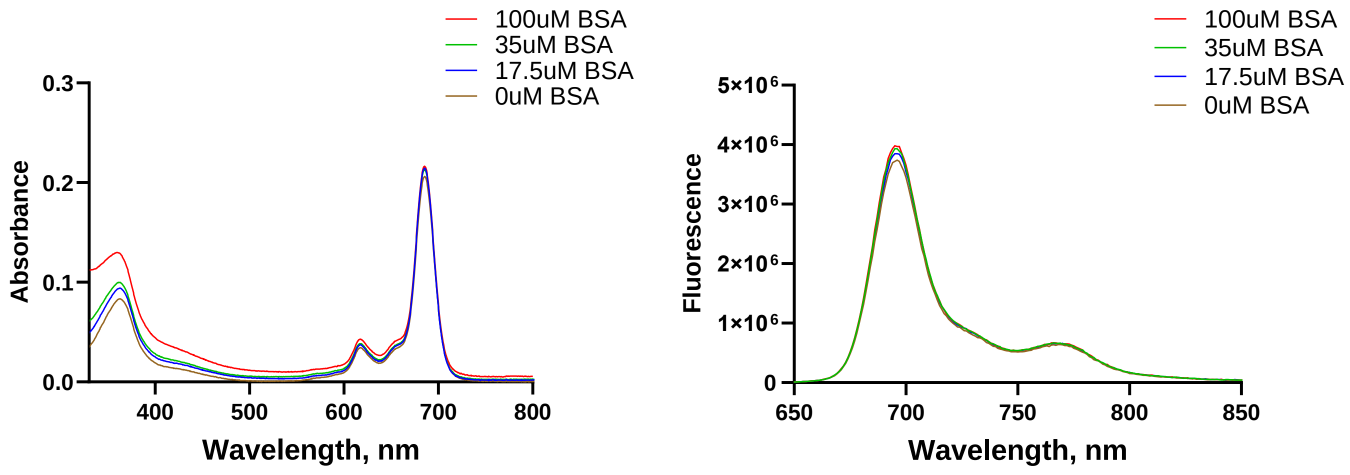

Fig. S77: Changes in absorption and fluorescence emission spectra of **N6Zn** (1µM) upon addition of bovine-serum albumin (BSA).

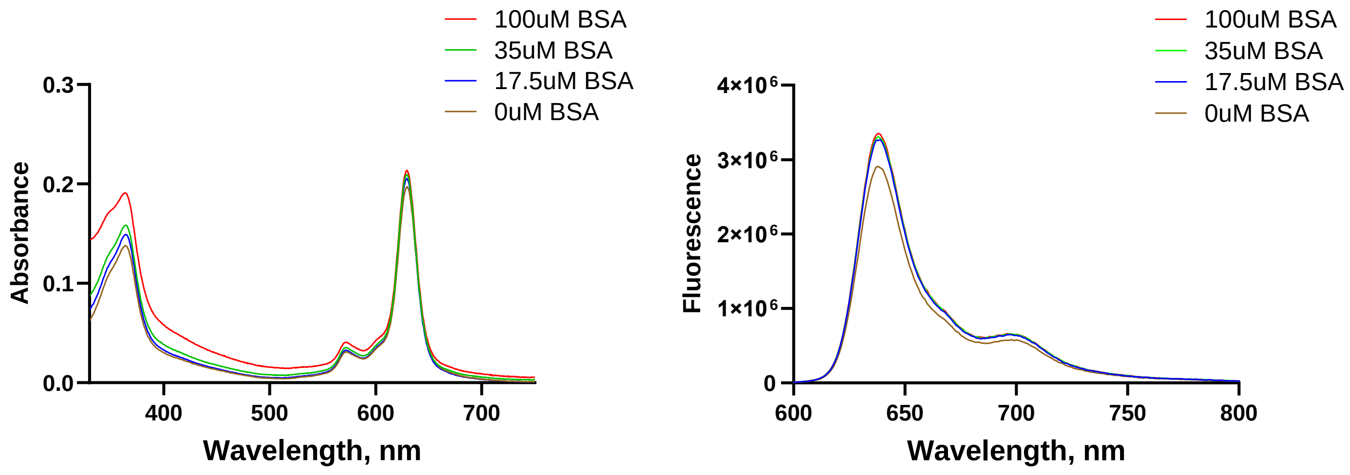

Fig. S78: Changes in absorption and fluorescence emission spectra of **N7Zn** (1µM) upon addition of bovine-serum albumin (BSA).

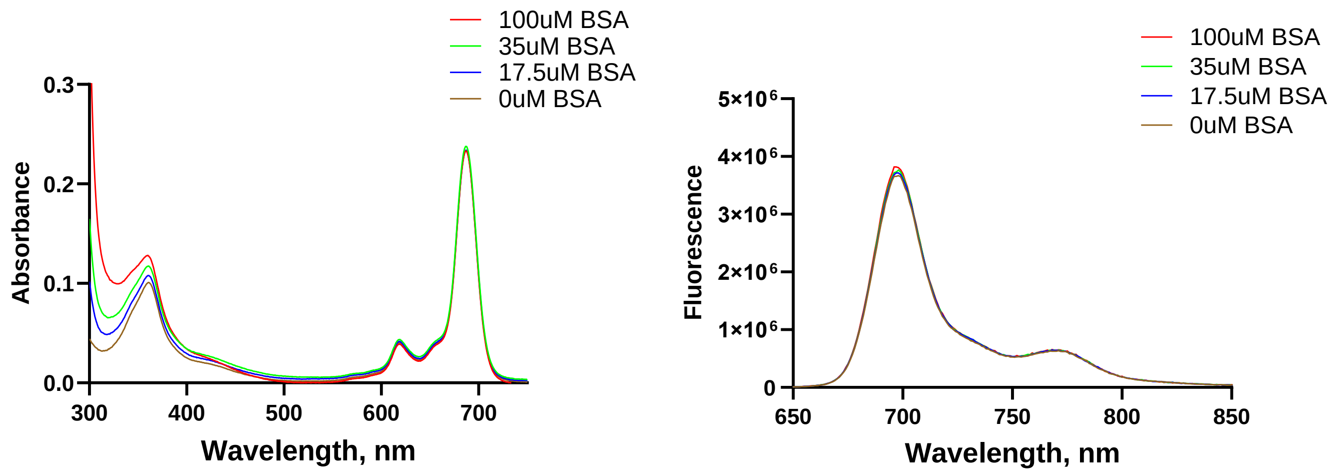

Fig. S79: Changes in absorption and fluorescence emission spectra of **N8Zn** (1µM) upon addition of bovine-serum albumin (BSA).

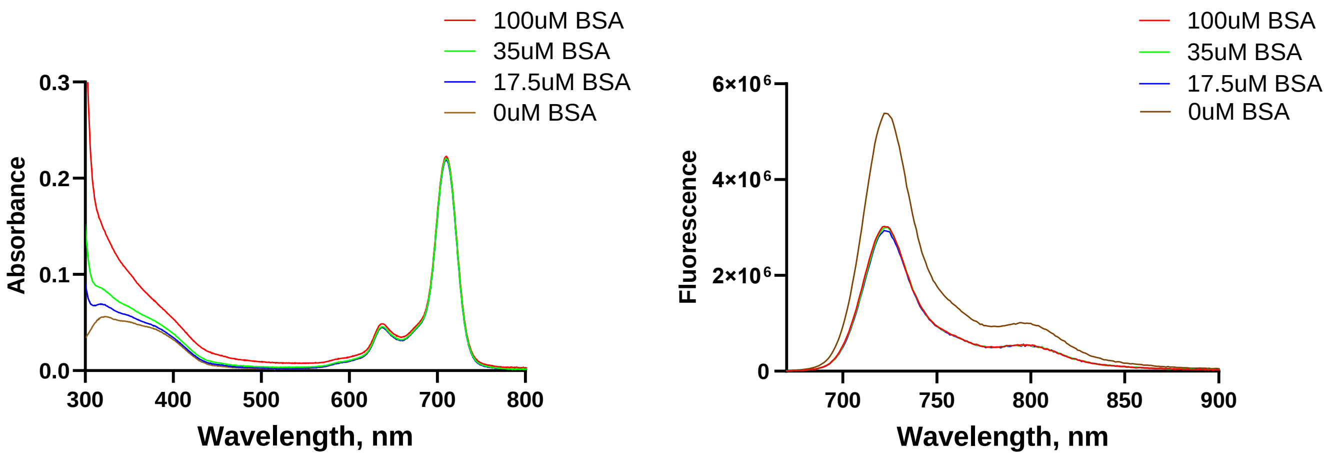

Fig. S80: Changes in absorption and fluorescence emission spectra of **N9Zn** (1μM) upon addition of bovine-serum albumin (BSA).

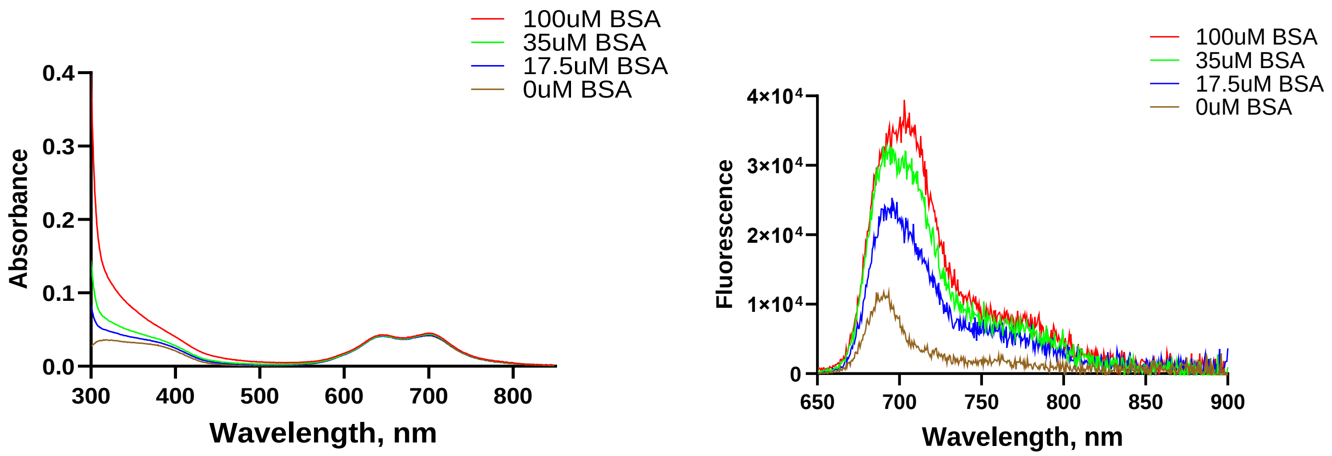

Fig. S81: Changes in absorption and fluorescence emission spectra of **N10Zn** (1μM) upon addition of bovine-serum albumin (BSA).

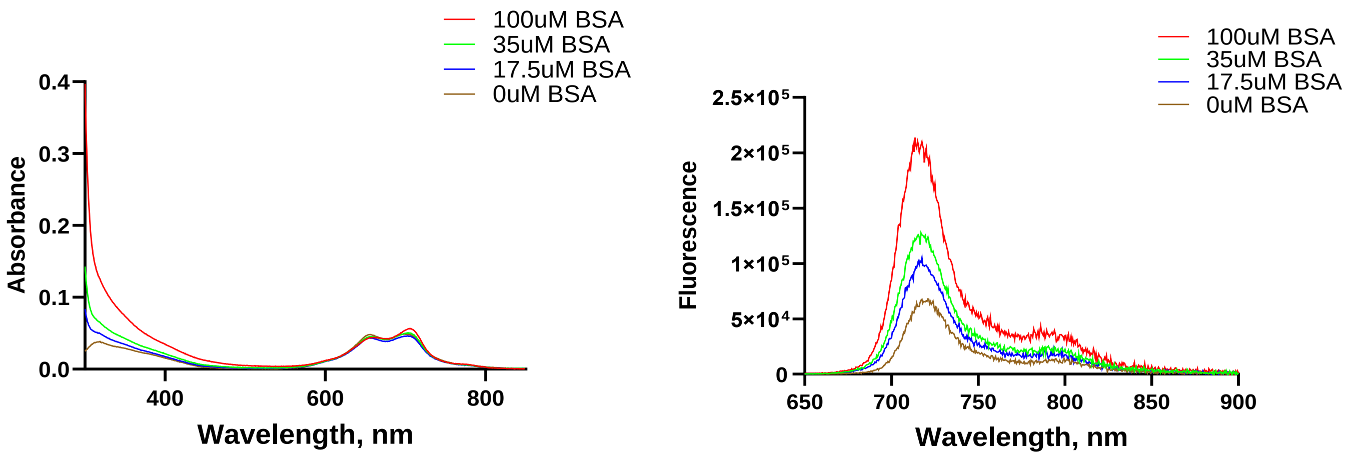

Fig. S82: Changes in absorption and fluorescence emission spectra of **N11Zn** (1μM) upon addition of bovine-serum albumin (BSA).

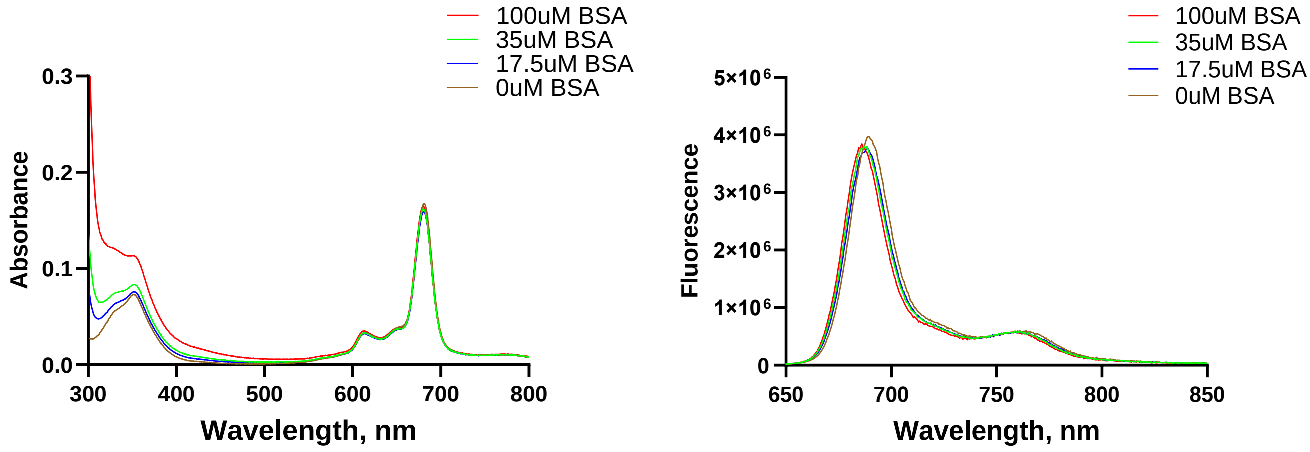

Fig. S83: Changes in absorption and fluorescence emission spectra of **Si1-C** (1µM) upon addition of bovine-serum albumin (BSA).

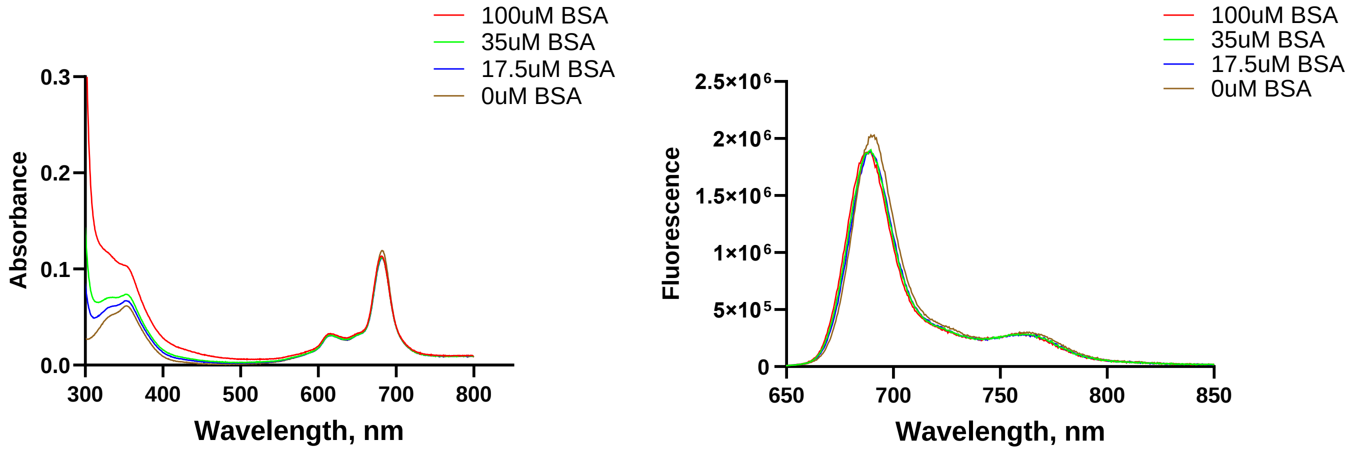

Fig. S84: Changes in absorption and fluorescence emission spectra of **Si2-C** (1µM) upon addition of bovine-serum albumin (BSA).

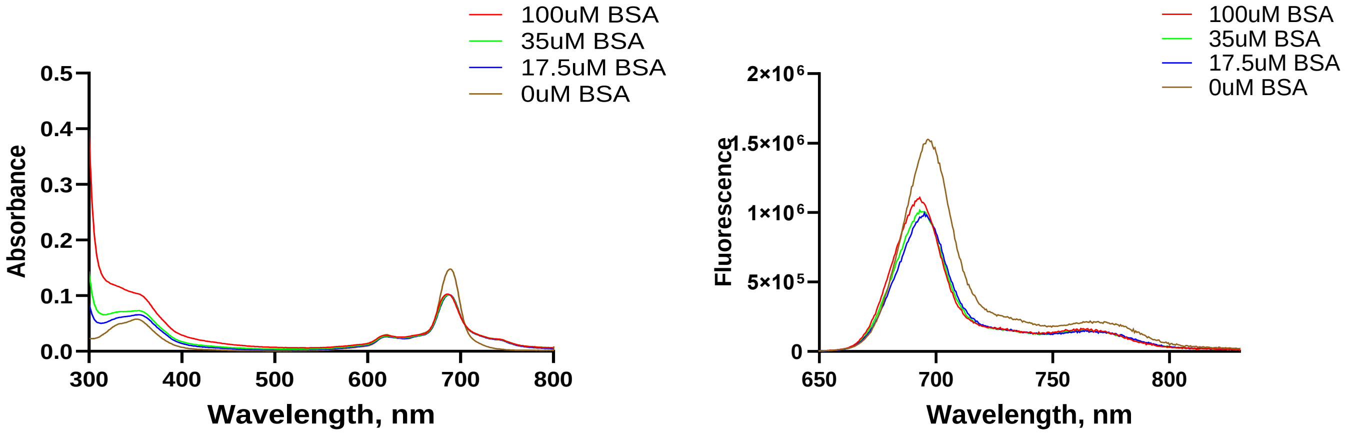

Fig. S85: Changes in absorption and fluorescence emission spectra of **Si3-C** (1µM) upon addition of bovine-serum albumin (BSA).

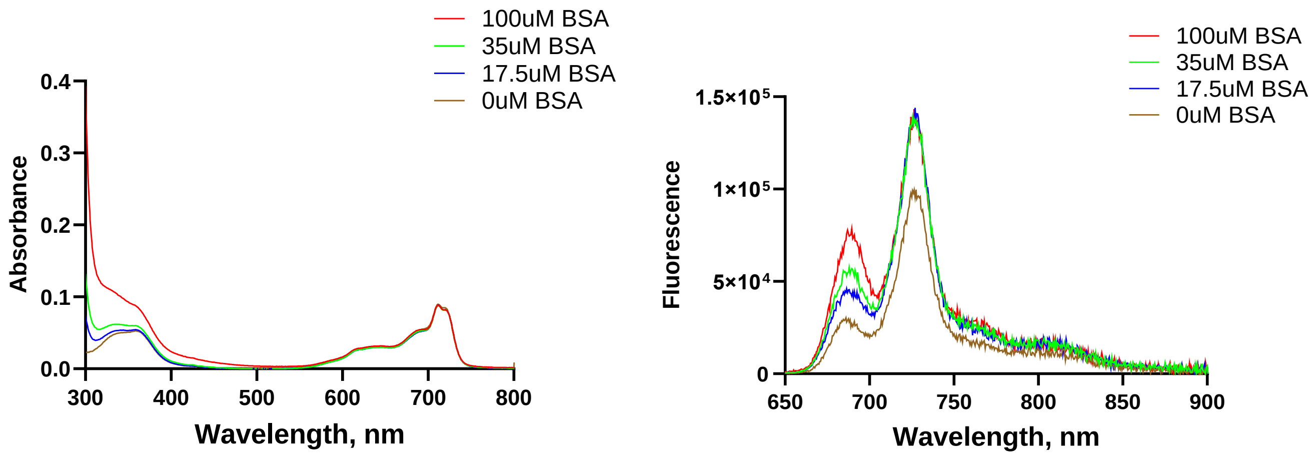

Fig. S86: Changes in absorption and fluorescence emission spectra of **Si4-C** (1µM) upon addition of bovine-serum albumin (BSA).

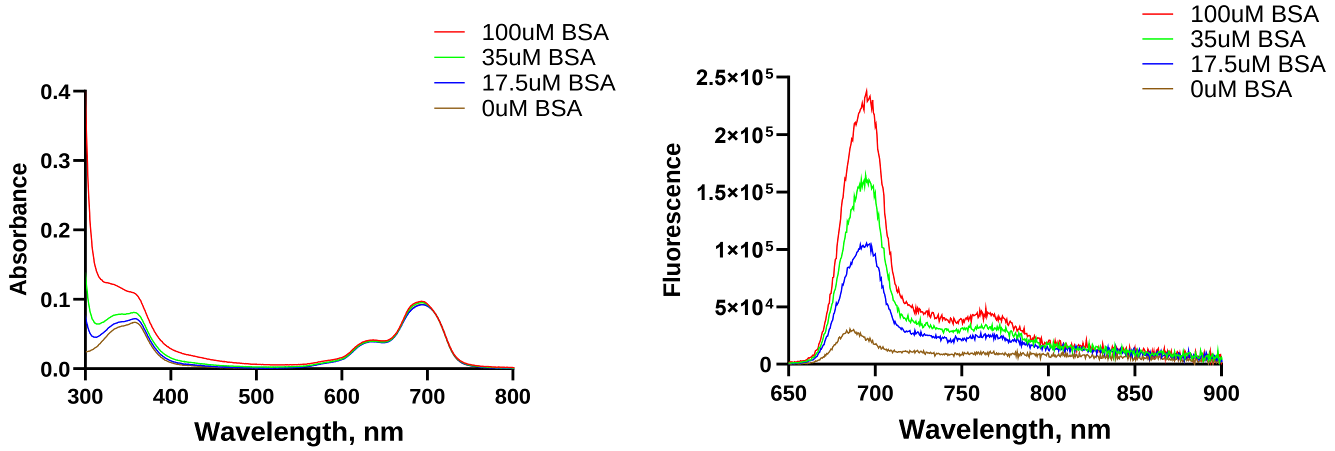

Fig. S87: Changes in absorption and fluorescence emission spectra of **Si5-N** (1µM) upon addition of bovine-serum albumin (BSA).

In vitro assessment of EC_{50} values on HeLa, SK-MEL-28 and MCF-7 cell lines

Anionic derivatives

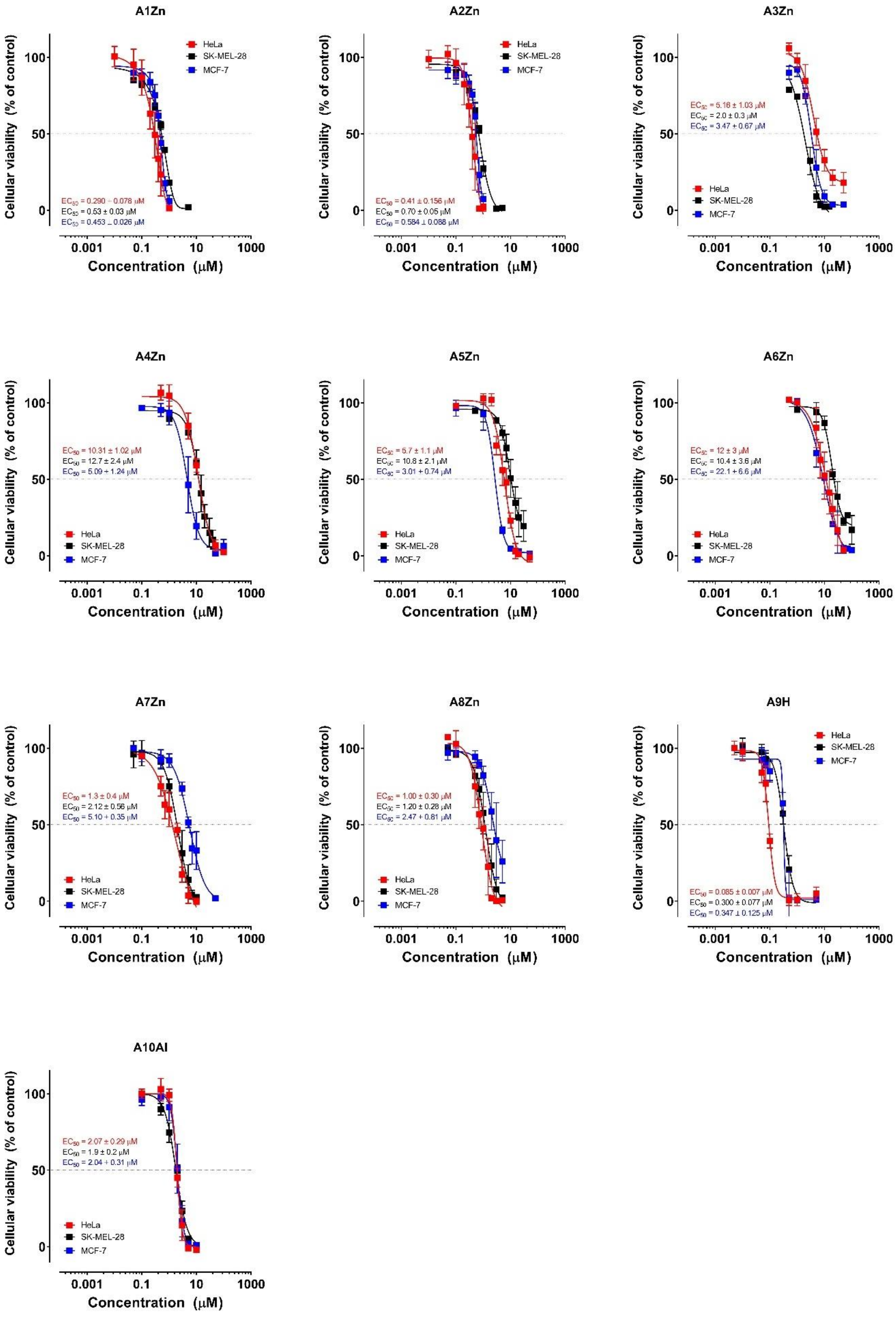

Fig. S88: Phototoxicity (EC_{50} values = mean ± SD, normalized to untreated control) of anionic derivatives on HeLa (red), SK-MEL-28 (black) and MCF-7 (blue) cell lines.

Cationic derivatives

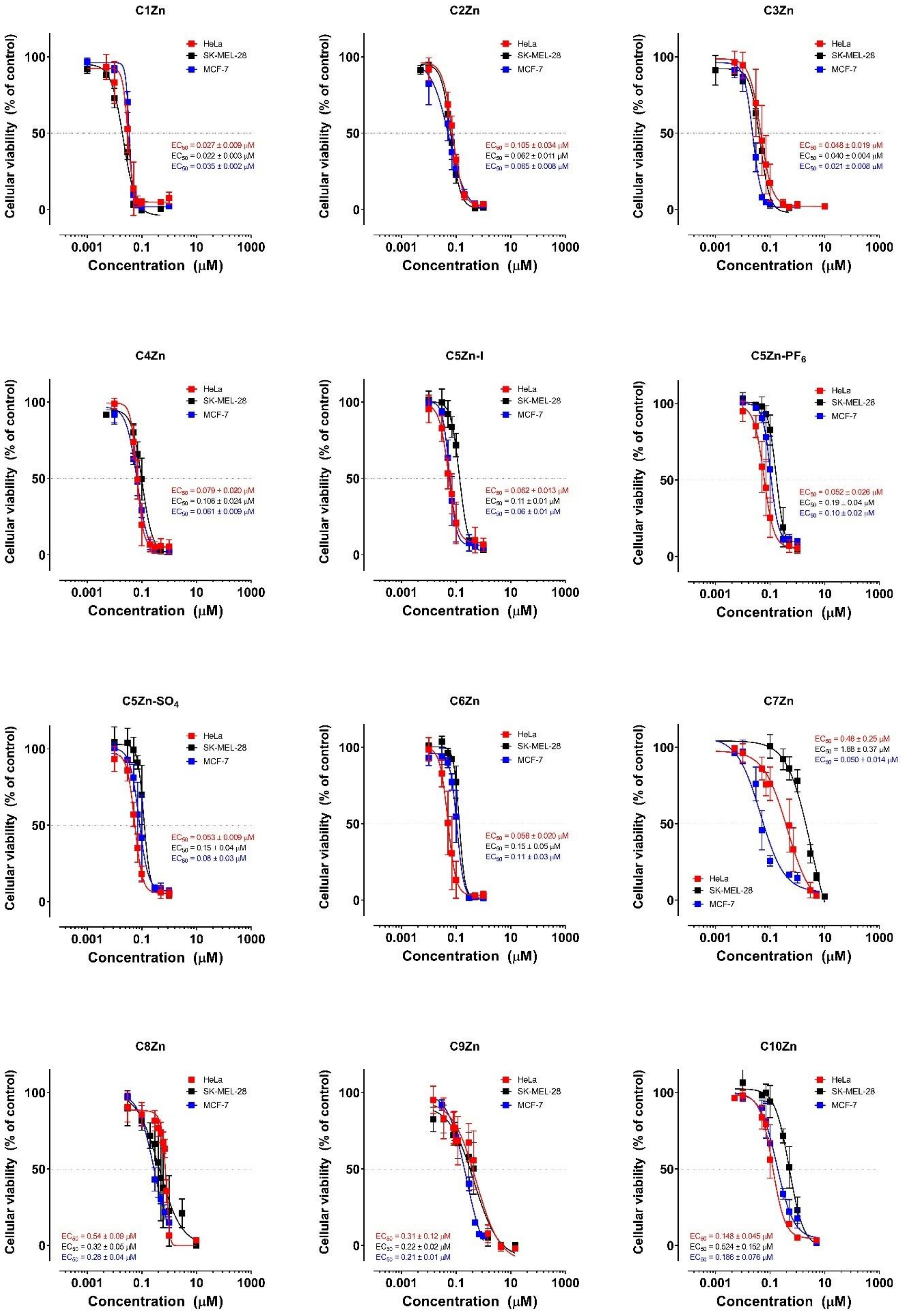

Fig. S89: Phototoxicity (EC$_{50}$ values = mean ± SD, normalized to untreated control) of cationic derivatives on HeLa (red), SK-MEL-28 (black) and MCF-7 (blue) cell lines.

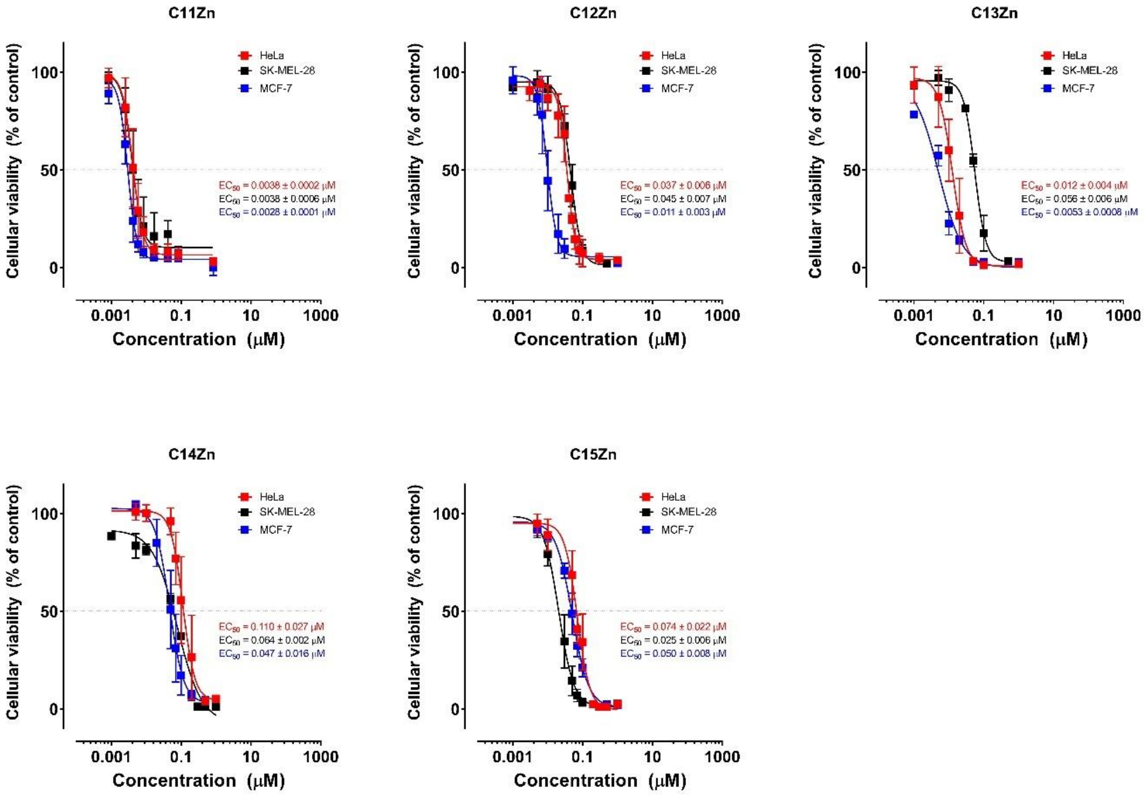

Fig. S90: Phototoxicity (EC_{50} values = mean ± SD, normalized to untreated control) of cationic derivatives on HeLa (red), SK-MEL-28 (black) and MCF-7 (blue) cell lines.

Neutral derivatives

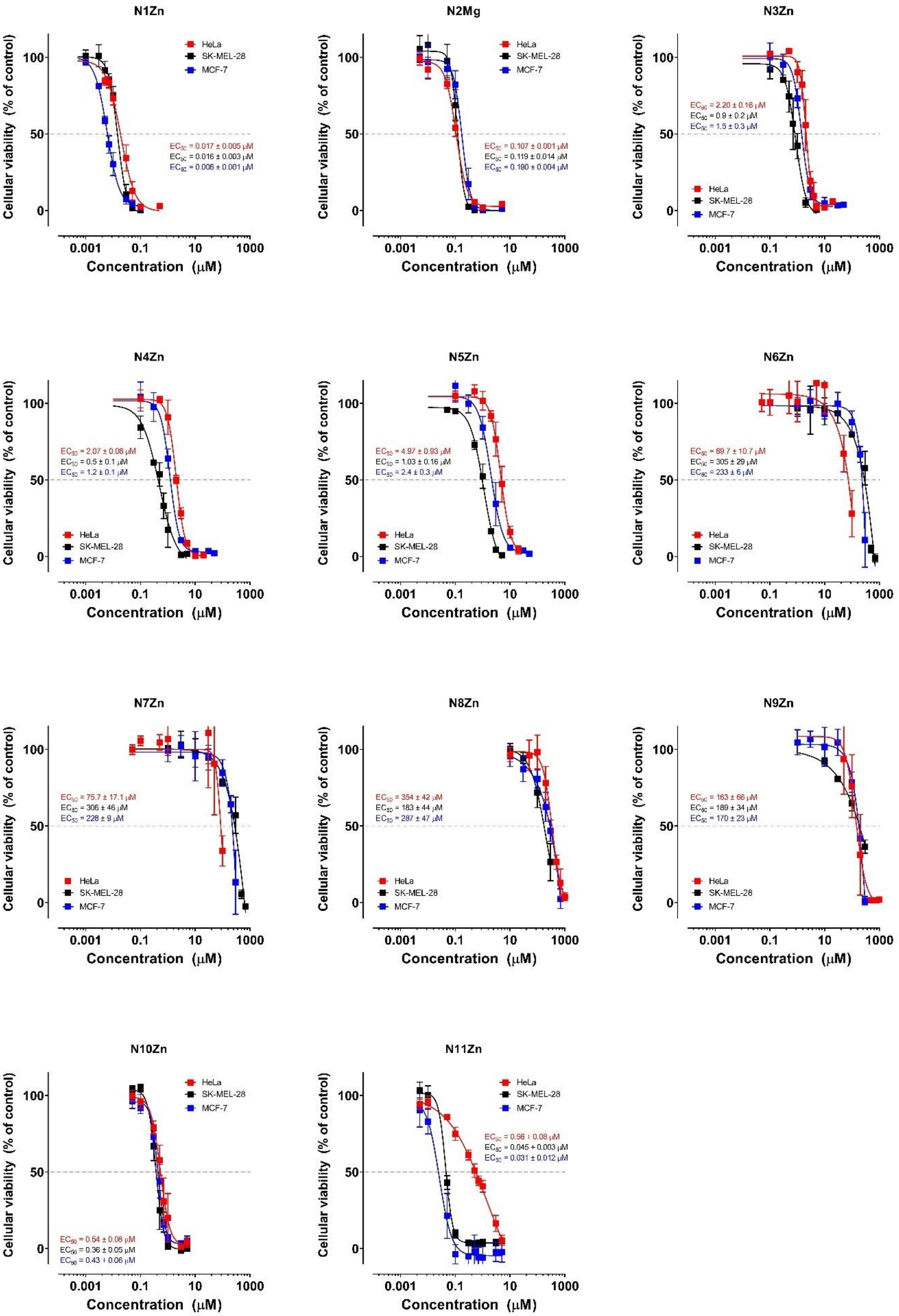

Fig. S91: Phototoxicity (EC_{50} values = mean ± SD, normalized to untreated control) of neutral derivatives on HeLa (red), SK-MEL-28 (black) and MCF-7 (blue) cell lines.

Si(IV) Pcs derivatives

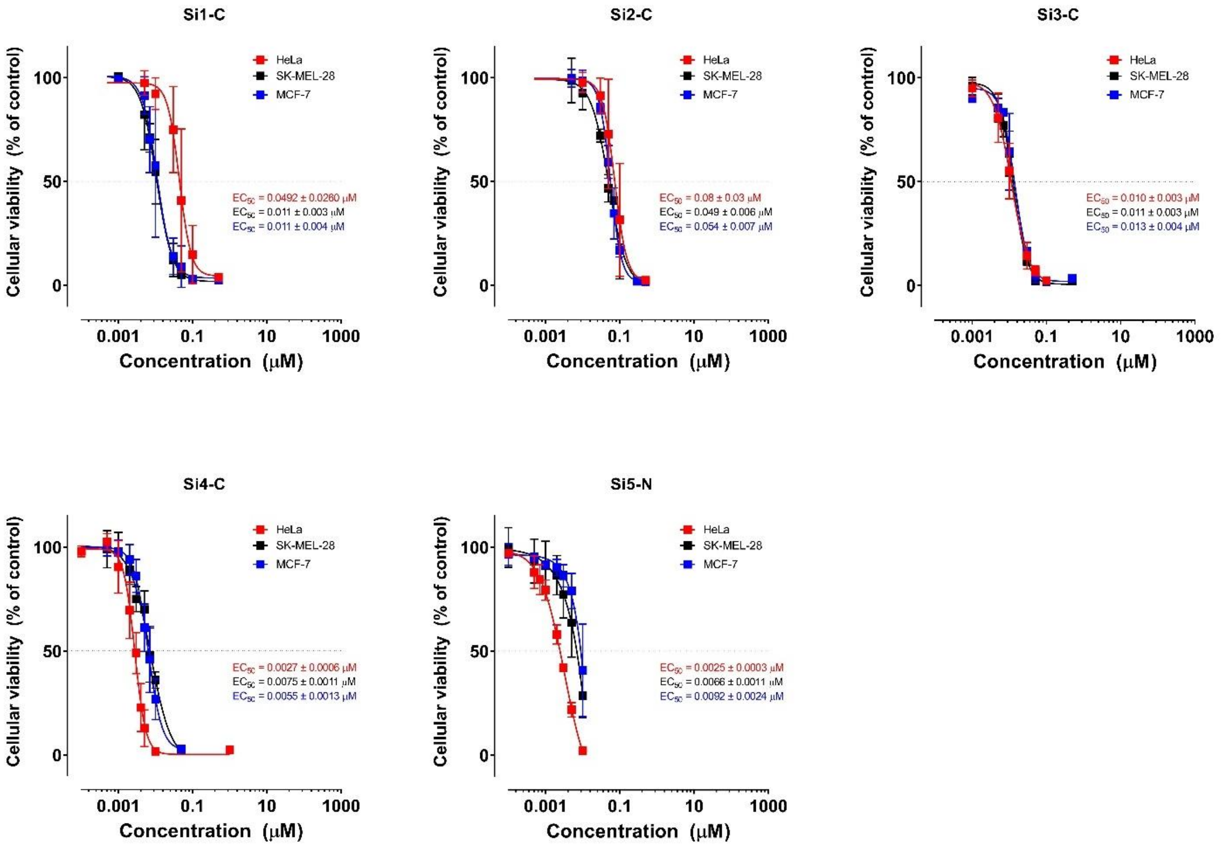

Fig. S92: Phototoxicity (EC_{50} values = mean ± SD, normalized to untreated control) of Si(IV) Pcs derivatives on HeLa (red), SK-MEL-28 (black) and MCF-7 (blue) cell lines.

Protoporphyrin IX, temoporfin and verteporfin

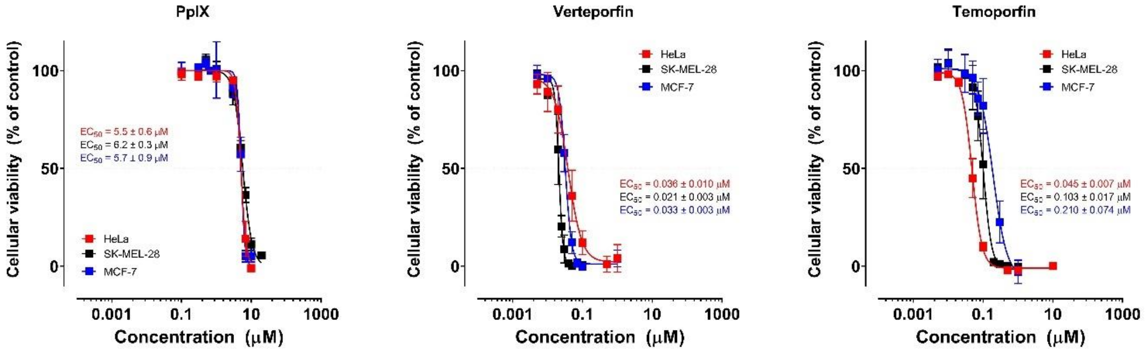

Fig. S93: Phototoxicity (EC_{50} values = mean ± SD, normalized to untreated control) of protoporphyrin IX (PpIX, an active form of prodrug 5-aminolevulinic acid), verteporfin and temoporfin on HeLa (red), SK-MEL-28 (black) and MCF-7 (blue) cell lines.

Subcellular localization on HeLa cells

Anionic derivatives

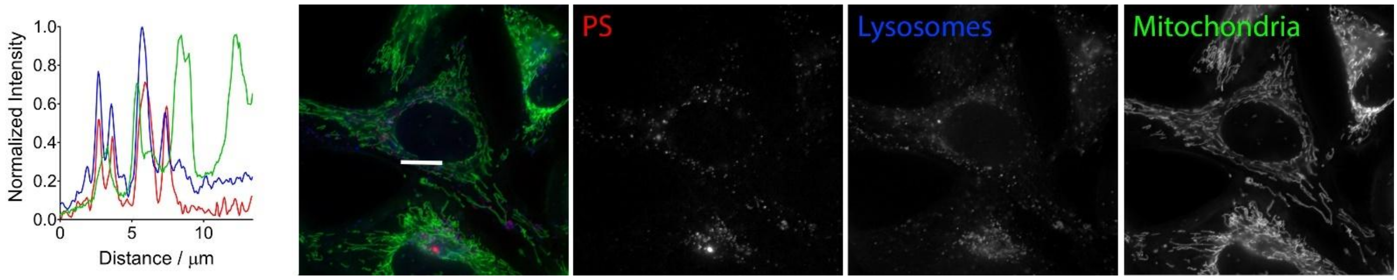

Fig. S94: Subcellular localization of **A6Zn** (0.1 μM). White bar in merged image represents 13.4 μm and indicates intensity profile measurement area. Green – mitochondria; blue – endolysosomal compartment; red – photosensitizer.

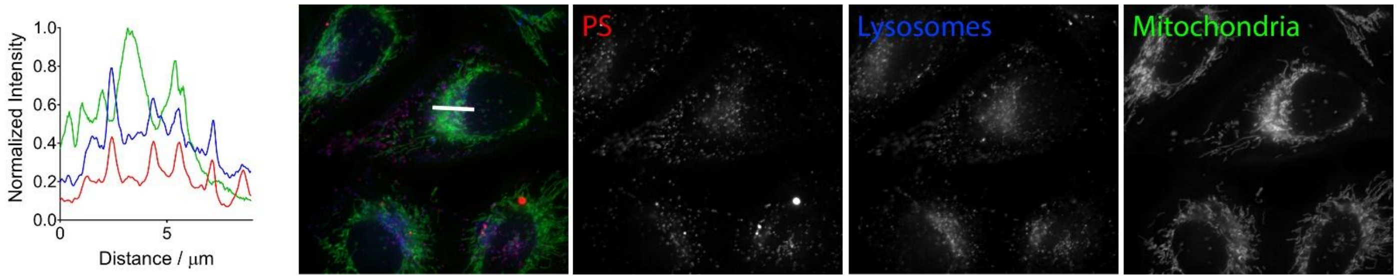

Fig. S95: Subcellular localization of **A7Zn** (0.1 μM). White bar in merged image represents 8.9 μm and indicates intensity profile measurement area. Green – mitochondria; blue – endolysosomal compartment; red – photosensitizer.

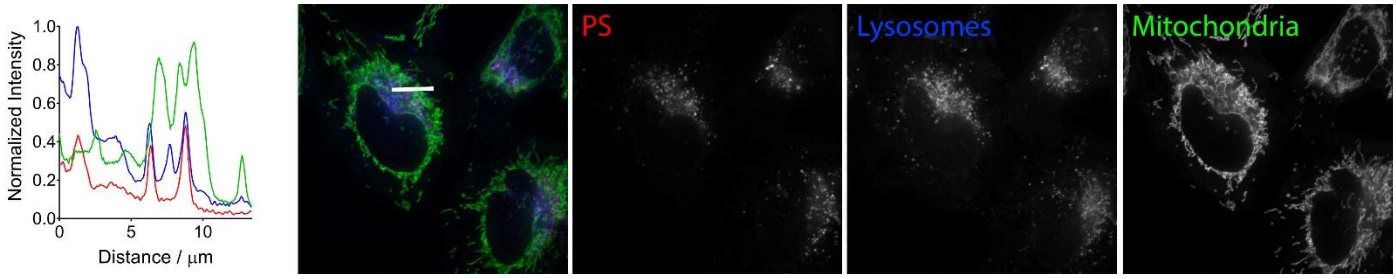

Fig. S96: Subcellular localization of **A8Zn** (0.1 µM). White bar in merged image represents 13.4 µm and indicates intensity profile measurement area. Green – mitochondria; blue – endolysosomal compartment; red – photosensitizer.

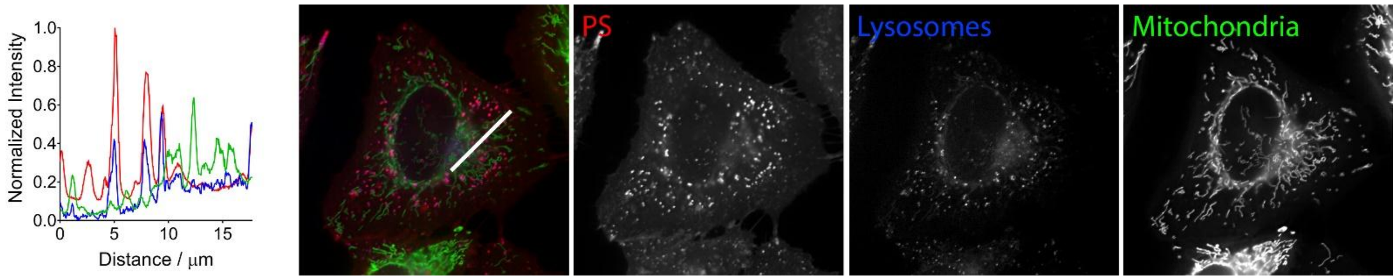

Fig. S97: Subcellular localization of **A9Zn** (0.1 µM). White bar in merged image represents 17.7 µm and indicates intensity profile measurement area. Green – mitochondria; blue – endolysosomal compartment; red – photosensitizer.

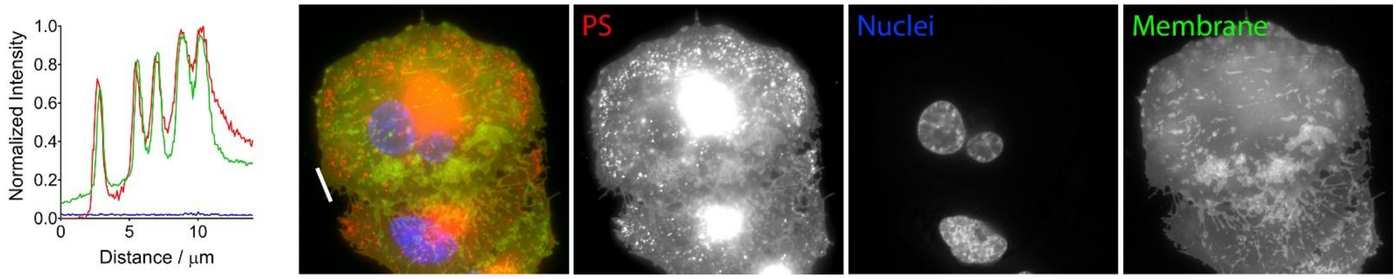

Fig. S98: Subcellular localization of **A9Zn** (1 µM). White bar in merged image represents 14.0 µm and indicates intensity profile measurement area. Green – membrane; blue – nuclei; red – photosensitizer.

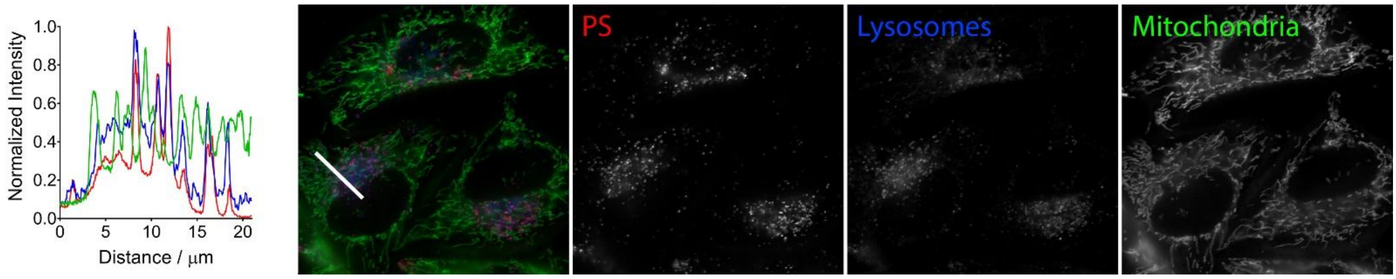

Fig. S99: Subcellular localization of **A10Zn** (0.1 µM). White bar in merged image represents 20.9 µm and indicates intensity profile measurement area. Green – mitochondria; blue – endolysosomal compartment; red – photosensitizer.

Cationic derivatives

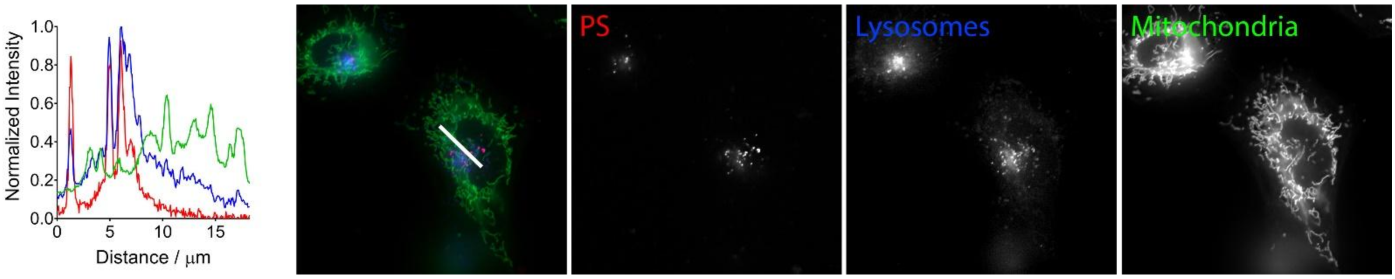

Fig. S100: Subcellular localization of **C5Zn-I** (0.1 µM). White bar in merged image represents 18.3 µm and indicates intensity profile measurement area. Green – mitochondria; blue – endolysosomal compartment; red – photosensitizer.

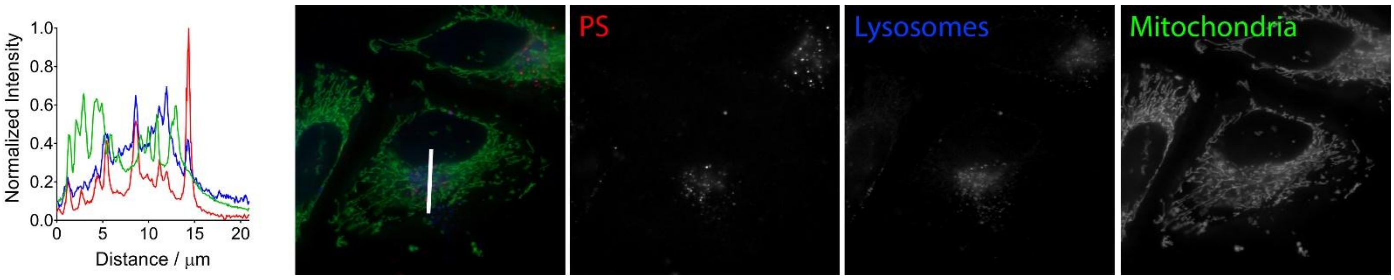

Fig. S101: Subcellular localization of **C5Zn-PF$_6$** (0.1 µM). White bar in merged image represents 20.9 µm and indicates intensity profile measurement area. Green – mitochondria; blue – endolysosomal compartment; red – photosensitizer.

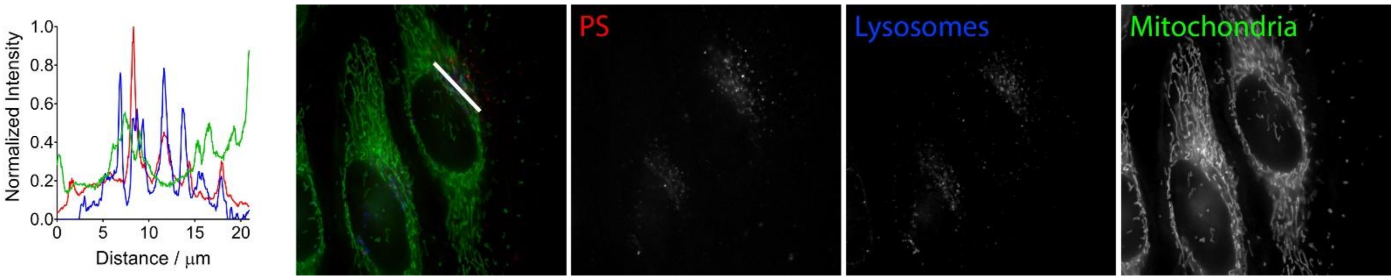

Fig. S102: Subcellular localization of **C5Zn-SO$_4$** (0.1 µM). White bar in merged image represents 20.9 µm and indicates intensity profile measurement area. Green – mitochondria; blue – endolysosomal compartment; red – photosensitizer.

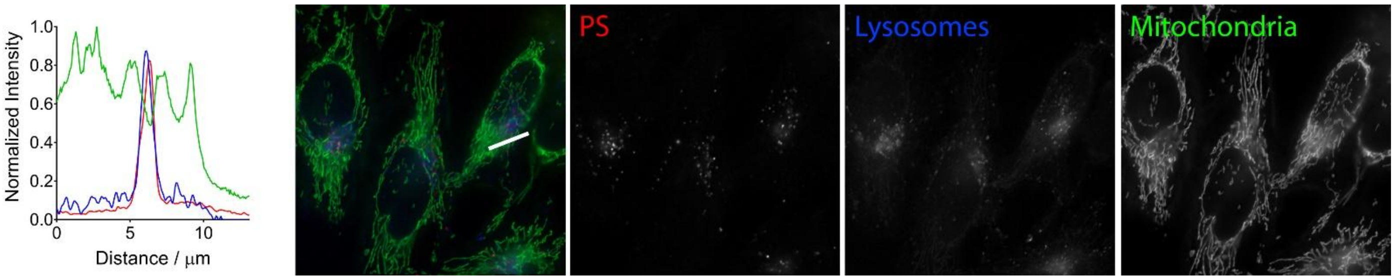

Fig. S103: Subcellular localization of **C6Zn** (0.5 µM). White bar in merged image represents 13.1 µm and indicates intensity profile measurement area. Green – mitochondria; blue – endolysosomal compartment; red – photosensitizer.

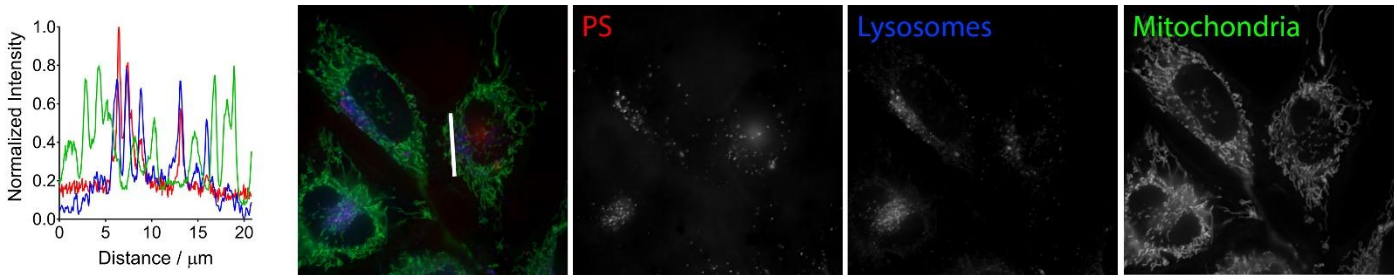

Fig. S104: Subcellular localization of **C10Zn** (0.1 µM). White bar in merged image represents 20.8 µm and indicates intensity profile measurement area. Green – mitochondria; blue – endolysosomal compartment; red – photosensitizer.

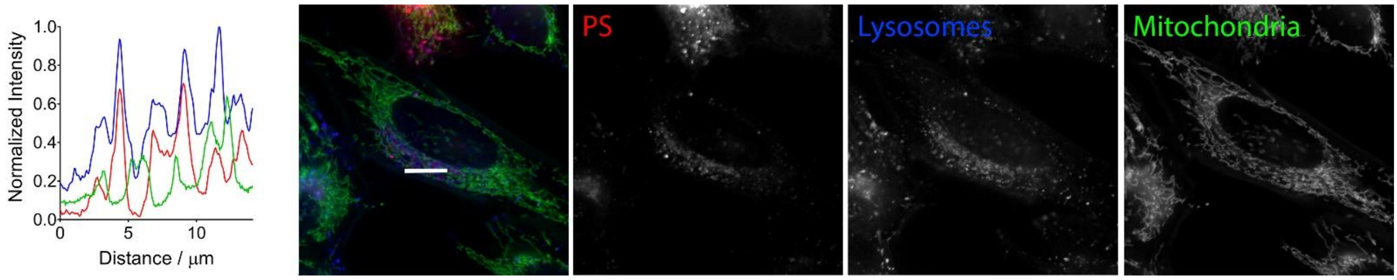

Fig. S105: Subcellular localization of **C15Zn** (0.1 µM). White bar in merged image represents 14.1 µm and indicates intensity profile measurement area. Green – mitochondria; blue – endolysosomal compartment; red – photosensitizer.

Neutral derivatives

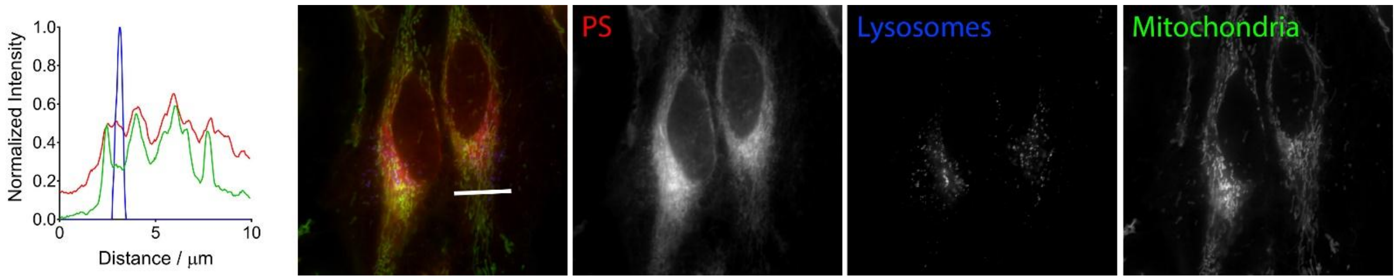

Fig. S106: Subcellular localization of **N1Zn** (1 μM). White bar in merged image represents 9.9 μm and indicates intensity profile measurement area. Green – mitochondria; blue – endolysosomal compartment; red – photosensitizer.

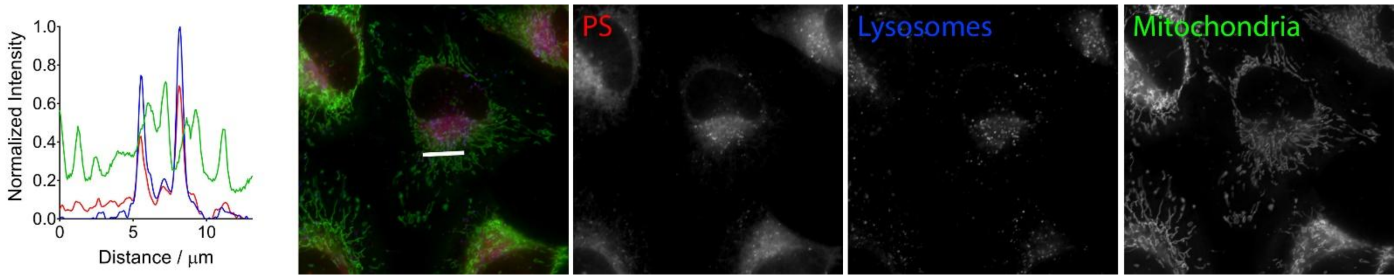

Fig. S107: Subcellular localization of **N2Mg** (1 μM). White bar in merged image represents 13.1 μm and indicates intensity profile measurement area. Green – mitochondria; blue – endolysosomal compartment; red – photosensitizer.

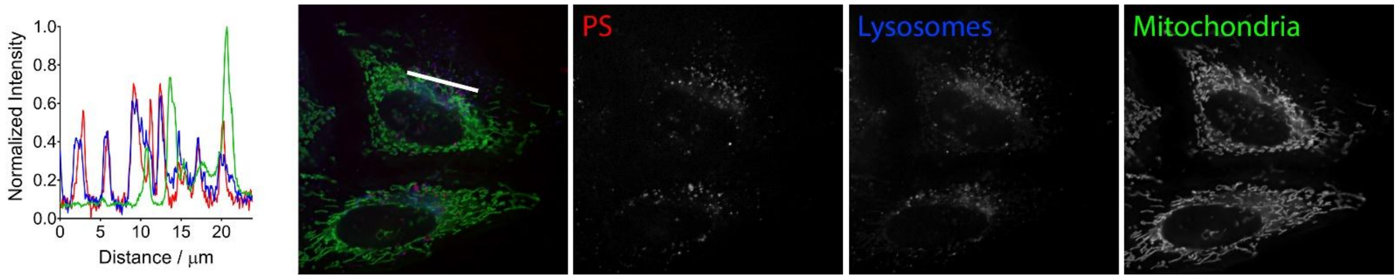

Fig. S108: Subcellular localization of **N6Zn** (0.1 μM). White bar in merged image represents 23.8 μm and indicates intensity profile measurement area. Green – mitochondria; blue – endolysosomal compartment; red – photosensitizer.

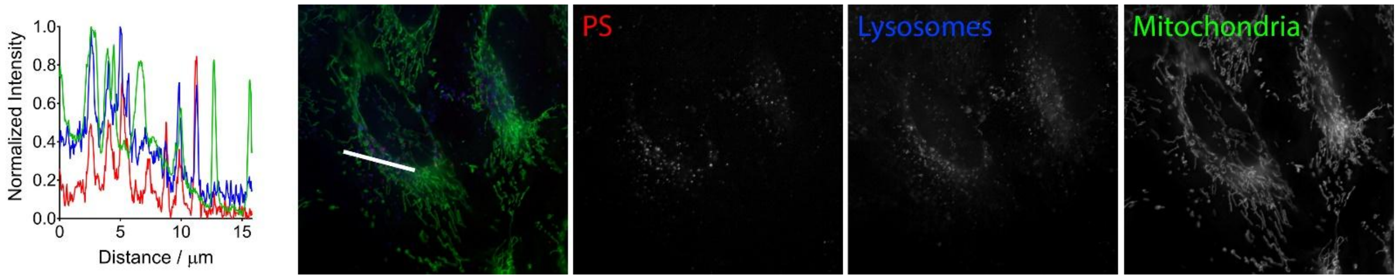

Fig. S109: Subcellular localization of **N7Zn** (0.1 μM). White bar in merged image represents 15.9 μm and indicates intensity profile measurement area. Green – mitochondria; blue – endolysosomal compartment; red – photosensitizer.

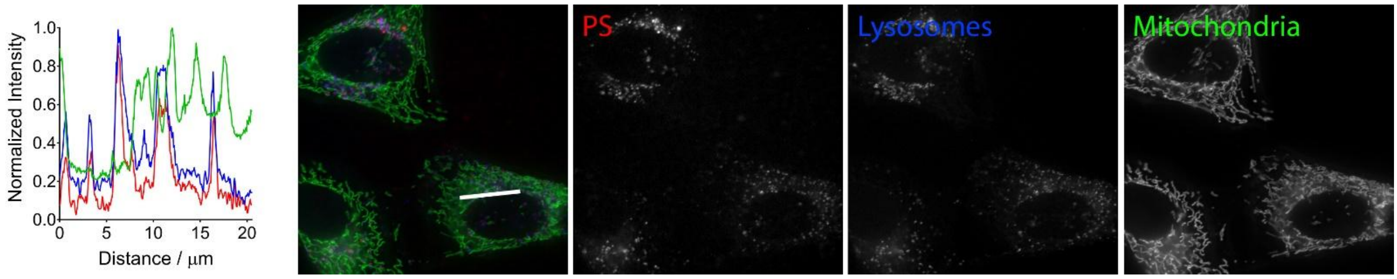

Fig. S110: Subcellular localization of **N8Zn** (1 μM). White bar in merged image represents 20.5 μm and indicates intensity profile measurement area. Green – mitochondria; blue – endolysosomal compartment; red – photosensitizer.

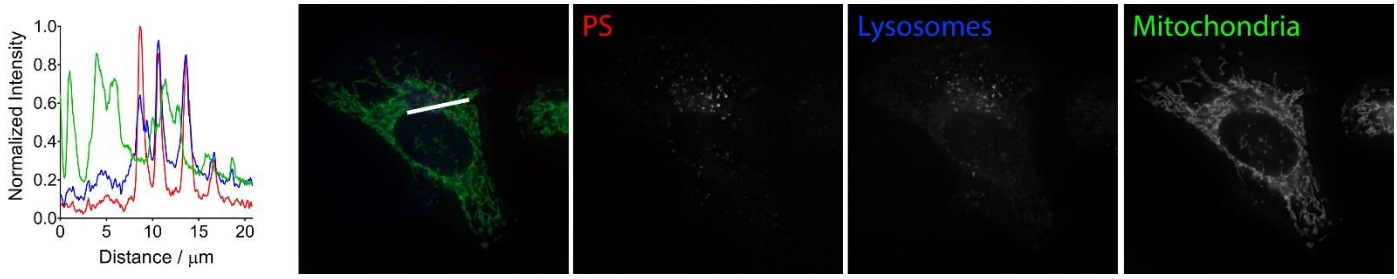

Fig. S111: Subcellular localization of **N9Zn** (0.1 μM). White bar in merged image represents 20.9 μm and indicates intensity profile measurement area. Green – mitochondria; blue – endolysosomal compartment; red – photosensitizer.

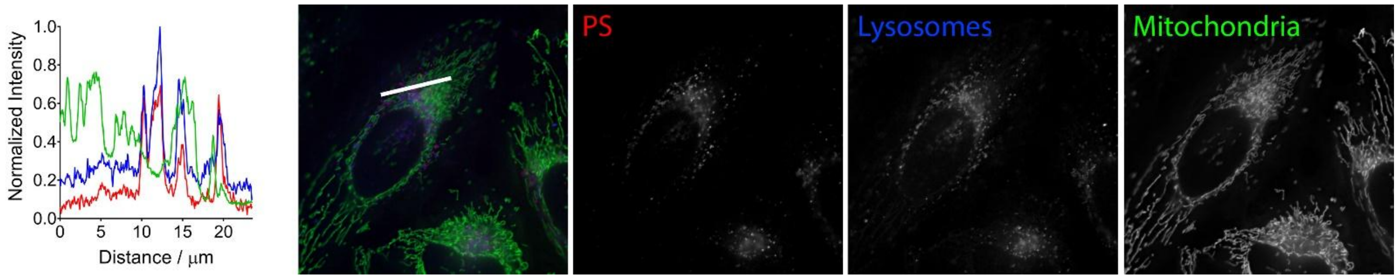

Fig. S112: Subcellular localization of **N10Zn** (0.1 μM). White bar in merged image represents 23.6 μm and indicates intensity profile measurement area. Green – mitochondria; blue – endolysosomal compartment; red – photosensitizer.

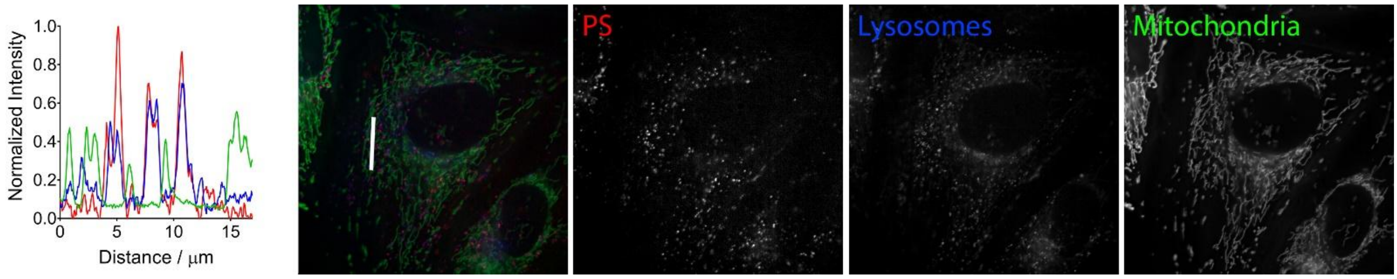

Fig. S113: Subcellular localization of **N11Zn** (0.1 μM). White bar in merged image represents 16.9 μm and indicates intensity profile measurement area. Green – mitochondria; blue – endolysosomal compartment; red – photosensitizer.

Si(IV) Pcs derivatives

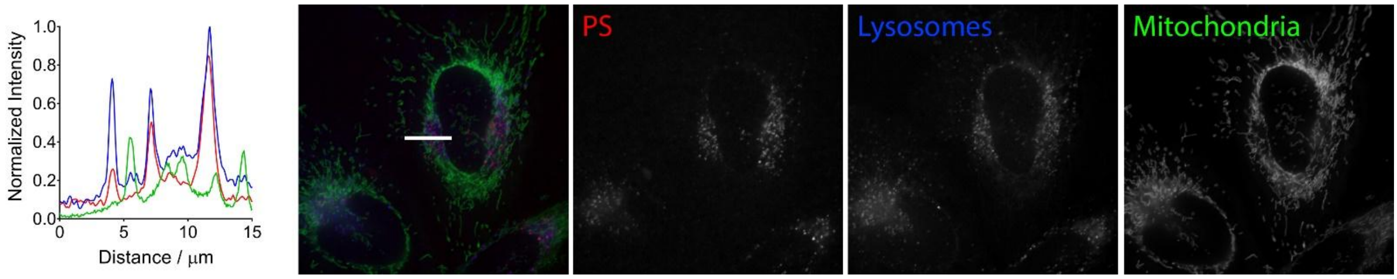

Fig. S114: Subcellular localization of **Si1-C** (0.1 μM). White bar in merged image represents 15.0 μm and indicates intensity profile measurement area. Green – mitochondria; blue – endolysosomal compartment; red – photosensitizer.

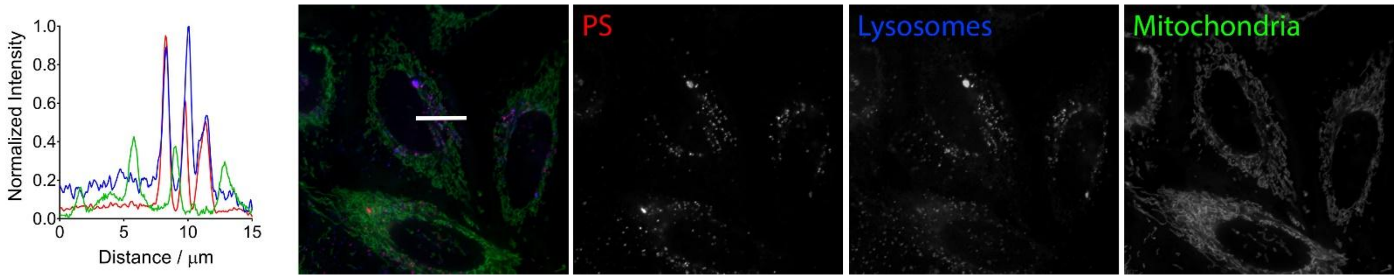

Fig. S115: Subcellular localization of **Si2-C** (0.1 μM). White bar in merged image represents 15.0 μm and indicates intensity profile measurement area. Green – mitochondria; blue – endolysosomal compartment; red – photosensitizer.

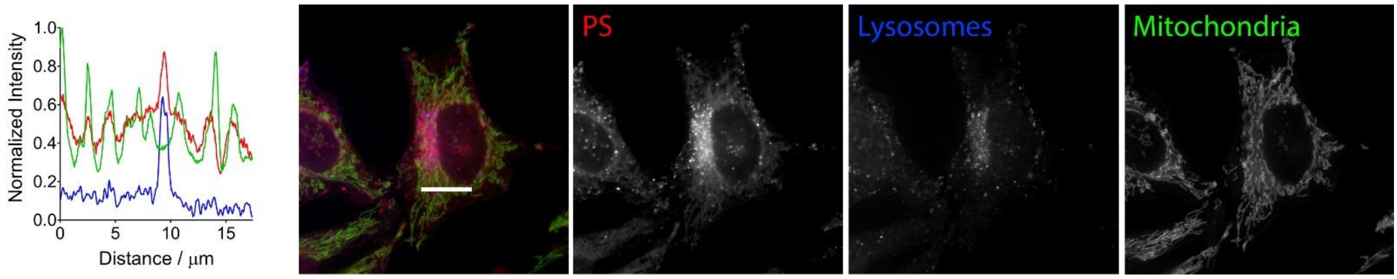

Fig. S116: Subcellular localization of **Si3-C** (0.1 μM). White bar in merged image represents 17.4 μm and indicates intensity profile measurement area. Green – mitochondria; blue – endolysosomal compartment; red – photosensitizer.

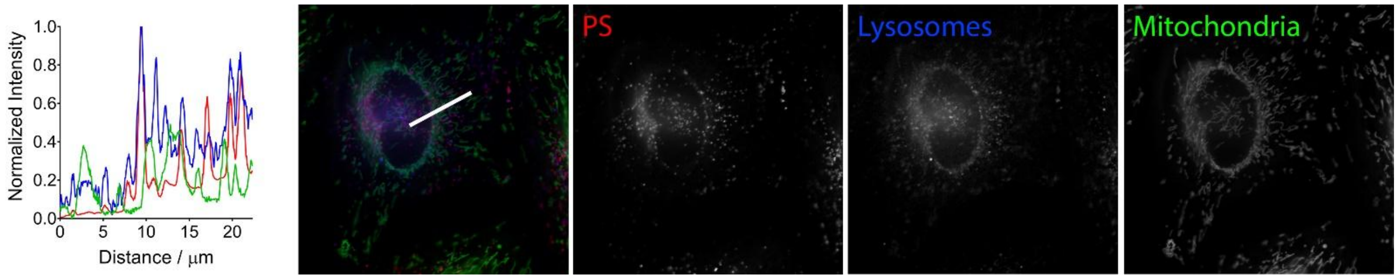

Fig. S117: Subcellular localization of **Si4-C** (0.05 μM). White bar in merged image represents 22.3 μm and indicates intensity profile measurement area. Green – mitochondria; blue – endolysosomal compartment; red – photosensitizer.

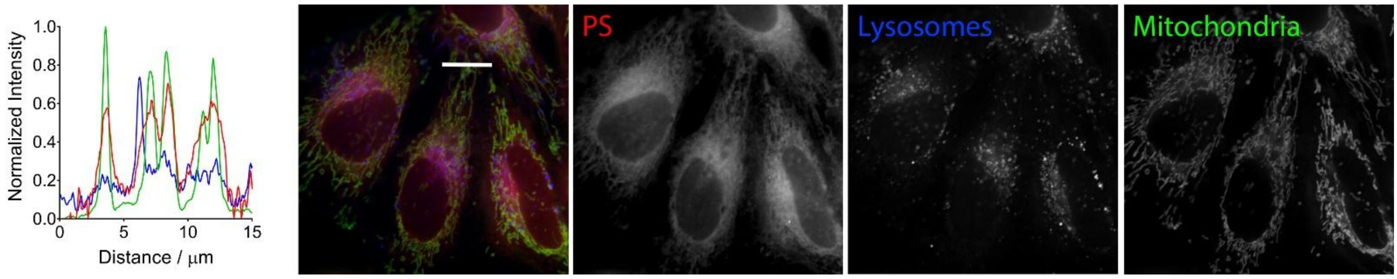

Fig. S118: Subcellular localization of **Si5-N** (0.1 μM). White bar in merged image represents 15.1 μm and indicates intensity profile measurement area. Green – mitochondria; blue – endolysosomal compartment; red – photosensitizer.

Statistical analysis

Comparing the effects of counterion using one-way ANOVA

EC_{50} values for C5Zn-I, C5Zn-PF_6 and C5Zn-SO_4 were compared to each other to reveal whether the counterion plays a significant role in photodynamic activity of these compounds using GraphPad Prism software version 10.4.1 (GraphPad Software, Boston, MA, USA). First, the data were subjected to Shapiro-Wilk test to assess whether the data distribution is normal (Gaussian). Since all data distribution was Gaussian and p-value for all data sets was $p > 0.05$, ordinary one-way ANOVA with *post hoc* Tukey test was used (multiple comparisons – mean of each group was compared with the mean of every other group). Analysis was performed on the results obtained from all cell lines – HeLa, MCF-7 and SK-MEL-28. In all cases, no statistical significance was found (Fig. S119).

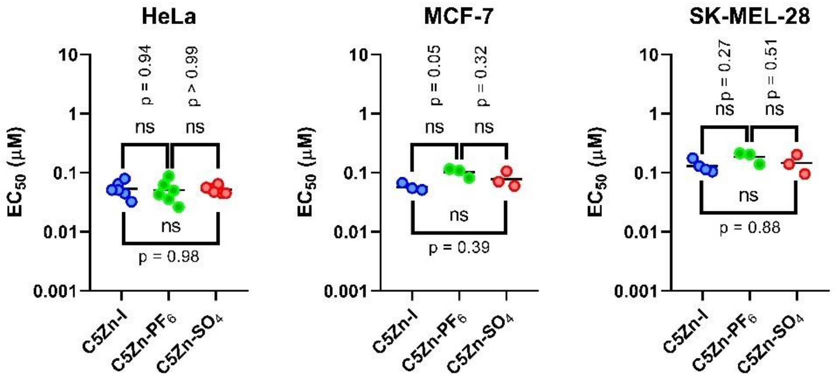

Fig. S119: Comparison of the effect of counterion in the cationic compounds C5Zn.

Comparing groups of photosensitizers using t-test

T-test was used to compare two groups of photosensitizers (their EC_{50} values) based on various structural features. First, Shapiro-Wilk test was used to determine the Gaussian distribution. If the $p \leq 0.05$ (non-Gaussian distribution) we proceeded with one-tailed Mann-Whitney test and if the $p > 0.05$ (Gaussian distribution) we proceeded with either one-tailed unpaired t-test (if the F value gives $p > 0.05$ indicating equal variance) or one-tailed Welch's t-test (if the F value gives $p \leq 0.05$ indicating non-equal variance). Note that some groups contain only two EC_{50} values (two compounds). In this case, one-tailed Welch's t-test was used (if the distribution of the second group was Gaussian) or one-tailed Mann-Whitney (if the distribution of the second group was non-Gaussian). These results are marked red in the graphs. Analysis was performed on HeLa (Fig. 6), MCF-7 (Fig. S120) and SK-MEL-28 (Fig. S121) cells. Compounds C5Zn-I, C5Zn-PF_6 and C5Zn-SO_4 are represented by mean value of their IC_{50} values (56, 80 and 150 nM for HeLa, MCF-7 and SK-MEL-28, respectively) as one compound, since ANOVA test didn't show any statistically significant differences (see Fig. S119).

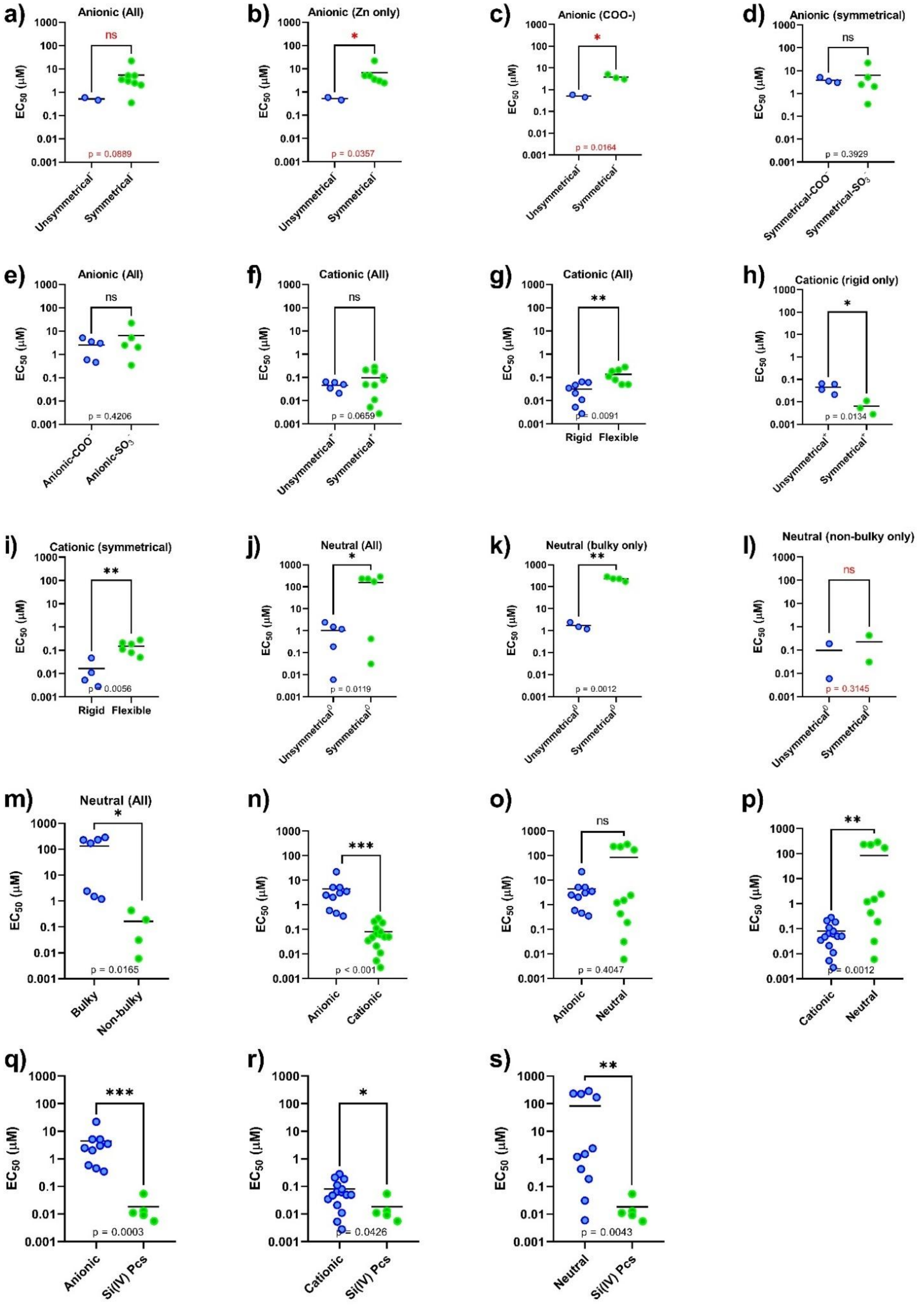

Fig. S120: Comparison of the photodynamic activities of different groups of photosensitizers against MCF-7 cells. Analysis is marked red, if at least one group contains only two members. a)-e) anionic compounds, f)-i) cationic compounds, j)-m) neutral compounds. n)-s) comparison of all main groups.

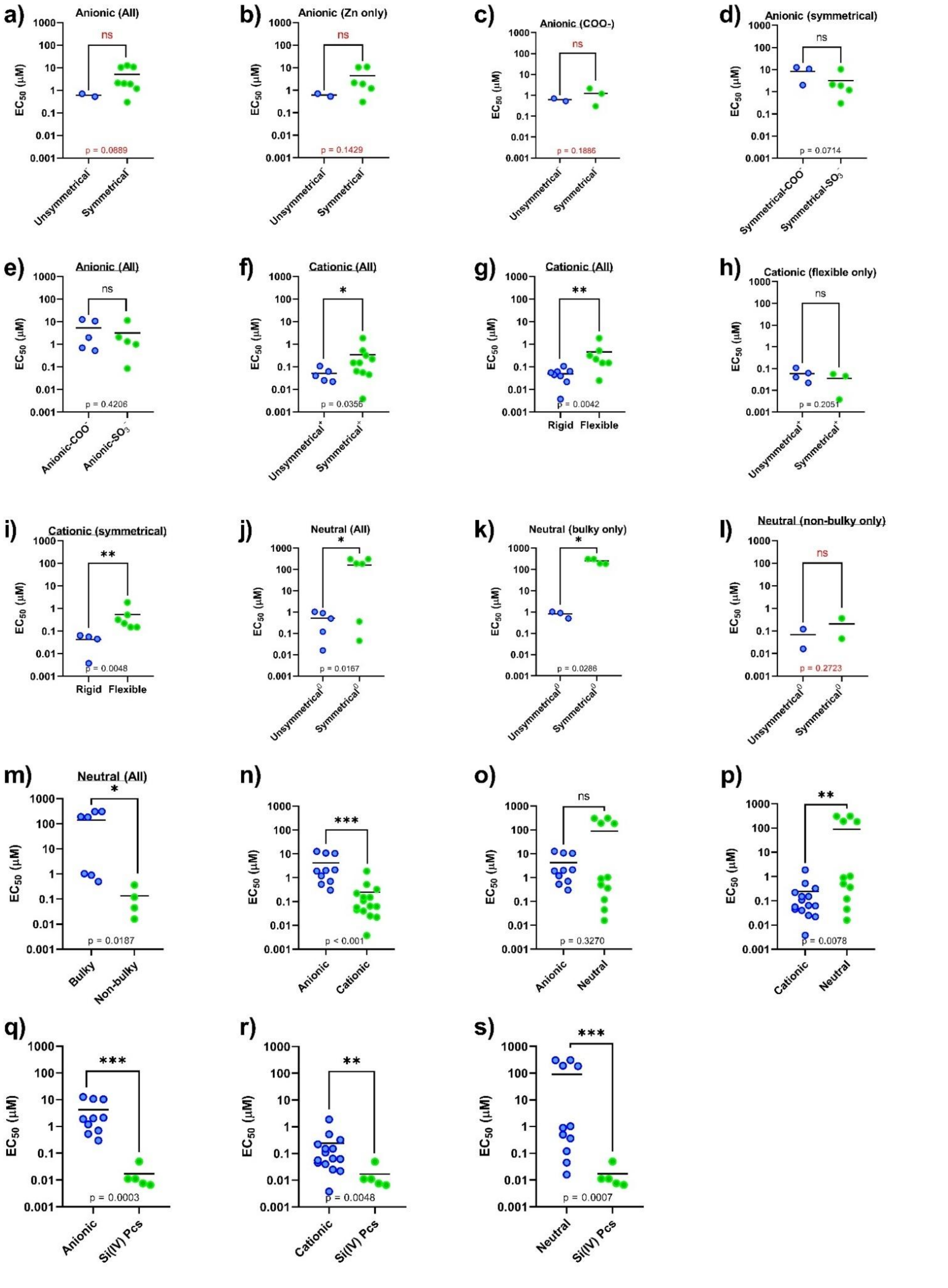

Fig. S121: Comparison of the photodynamic activities of different groups of photosensitizers against SK-MEL-28 cells. Analysis is marked red, if at least one group contains only two members. a)-e) anionic compounds, f)-i) cationic compounds, j)-m) neutral compounds. n)-s) comparison of all main groups.

Overview of EC_{50} values

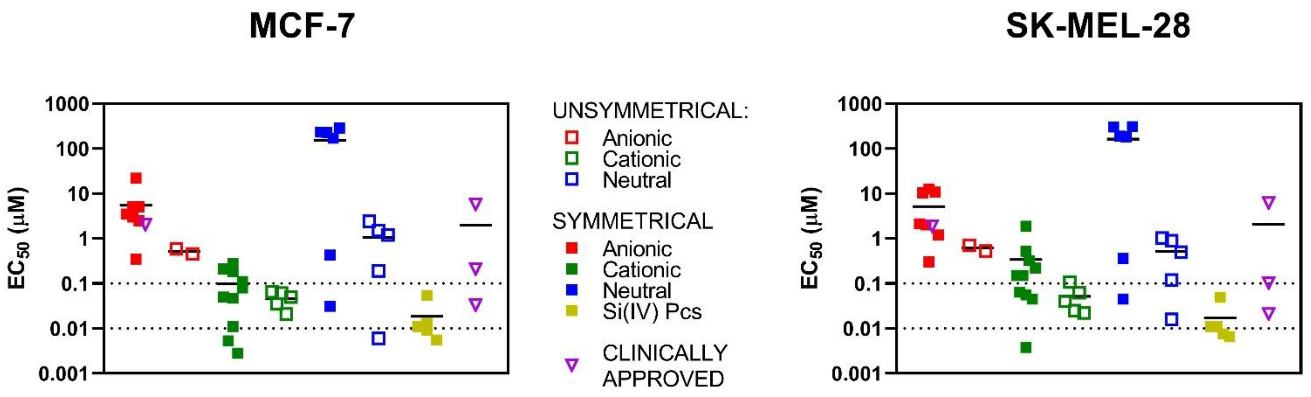

Fig. S122: Graphical overview of EC_{50} of all studied compounds on MCF-7 and SK-MEL-28 cell lines (HeLa is depicted on Fig. 4 A in the article). Compound **A10Al** (Photosens) is included in "symmetrical anionic" group (red full squares) and marked as a purple open triangle (clinically approved).

Overview of the differences among cell lines

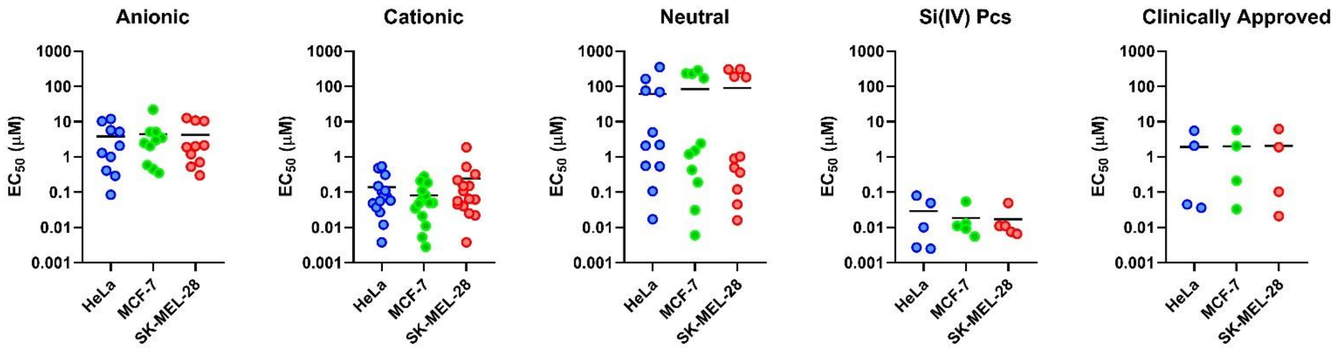

Fig. S123: Graphical interpretation of the differences in EC_{50} values between HeLa (blue), MCF-7 (green) and SK-MEL-28 (red) cell lines.

Statistical evaluation of differences in EC_{50} values of individual compounds among cell lines using one-way ANOVA

Statistical analysis of EC_{50} values for each photosensitizer was performed using GraphPad Prism software version 10.4.1 (GraphPad Software, Boston, MA, USA). First, the data were subjected to Shapiro-Wilk test to assess whether the data distribution is normal (Gaussian). If the data distribution was Gaussian and F-value for all data sets was $p > 0.05$, ordinary one-way ANOVA with *post hoc* Tukey test was used. If the data distribution was not Gaussian, Kruskal-Wallis test with *post hoc* Dunn's test was used (Fig. S124-S128).

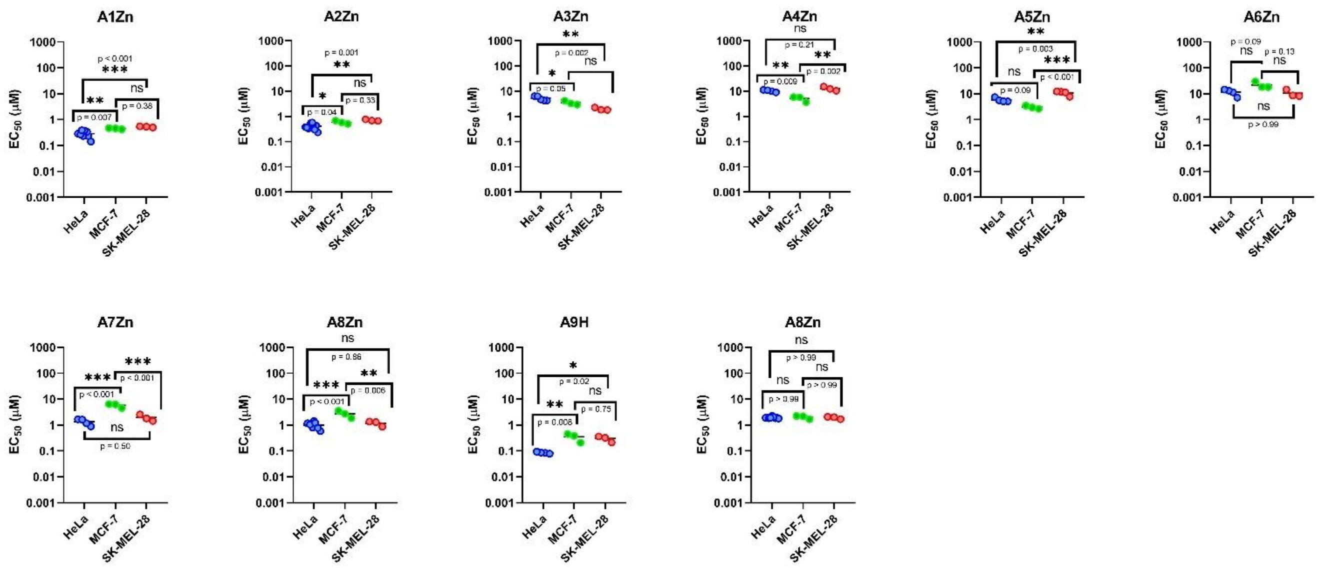

Fig. S124: Analysis of anionic compounds (A-series); HeLa (blue), MCF-7 (green) and SK-MEL-28 (red).

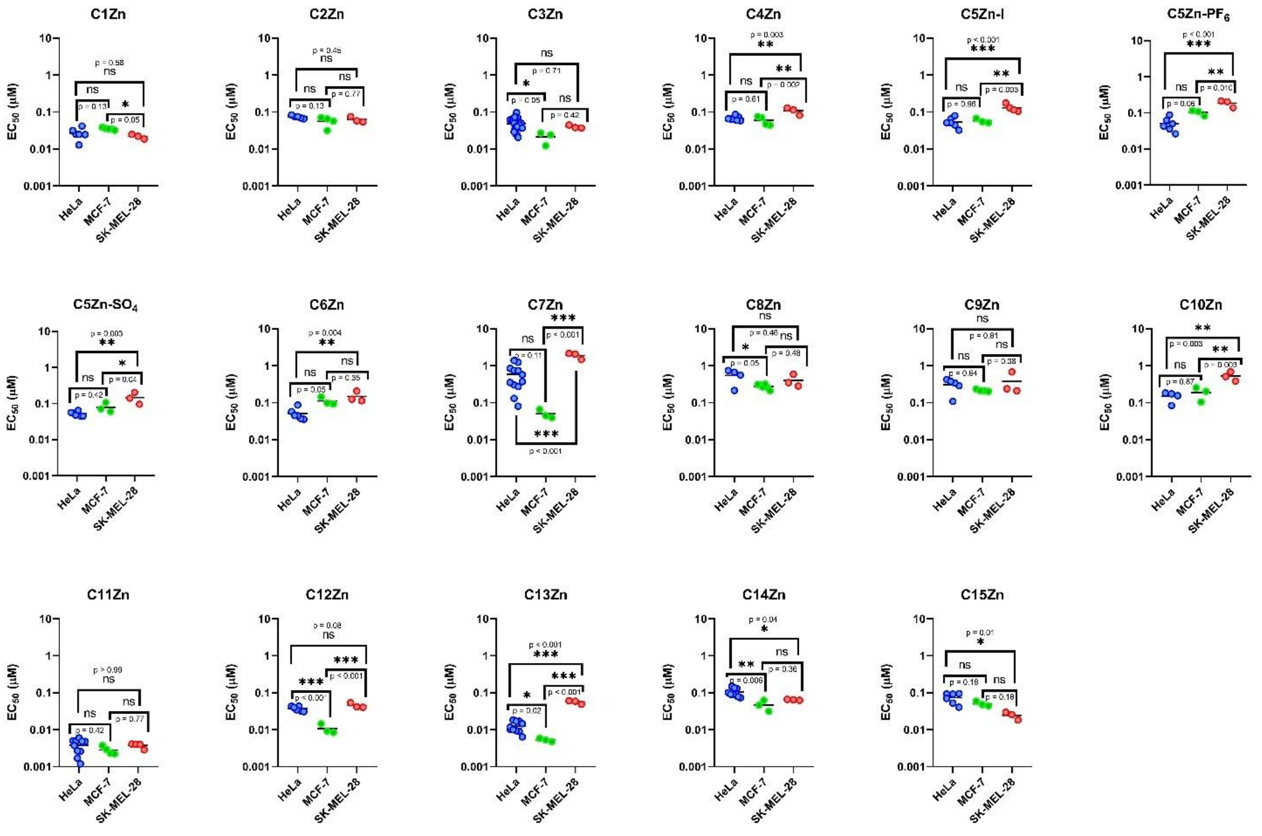

Fig. S125: Analysis of cationic compounds (C-series); HeLa (blue), MCF-7 (green) and SK-MEL-28 (red).

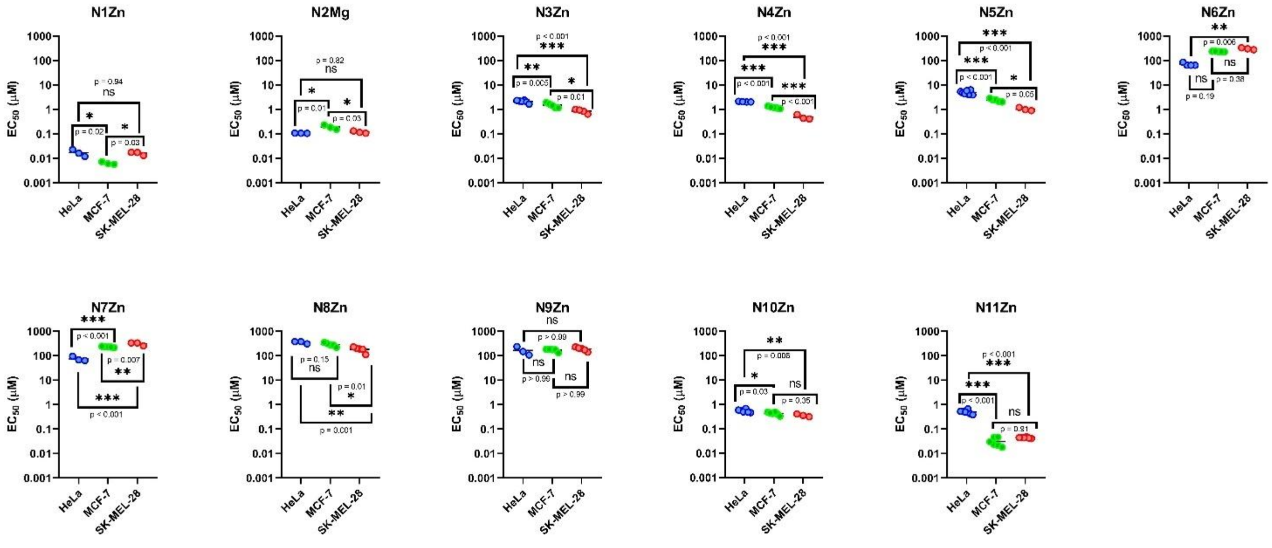

Fig. S126: Analysis of neutral compounds (N-series); HeLa (blue), MCF-7 (green) and SK-MEL-28 (red).

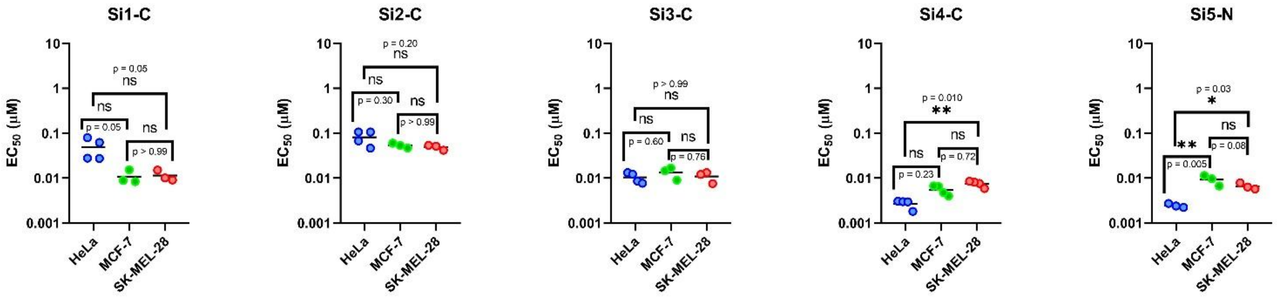

Fig. S127: Analysis of compounds with axial ligands (Si-series); HeLa (blue), MCF-7 (green) and SK-MEL-28 (red).

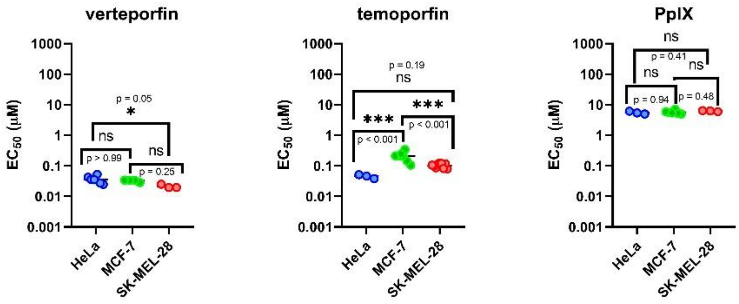

Fig. S128: Analysis of clinically used compounds (including A10-Al); HeLa (blue), MCF-7 (green) and SK-MEL-28 (red).

Spectrum of the lamp for *in vitro* studies

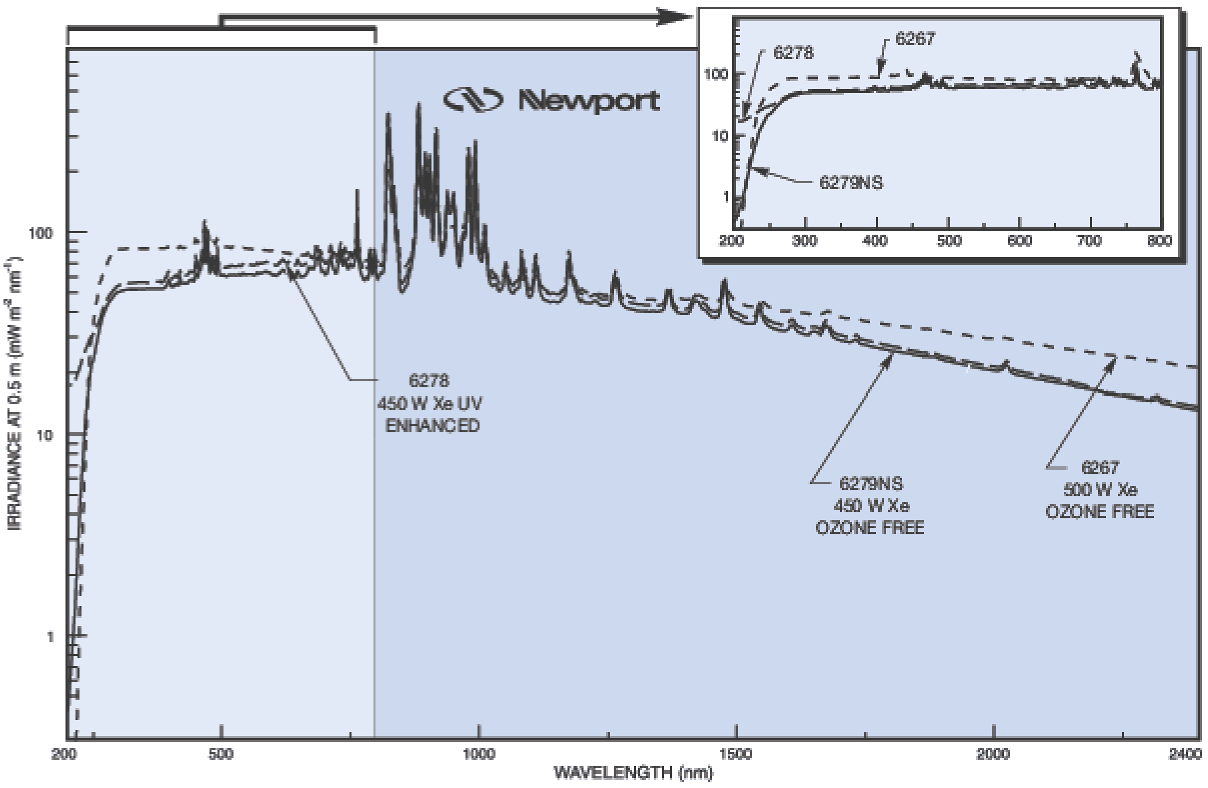

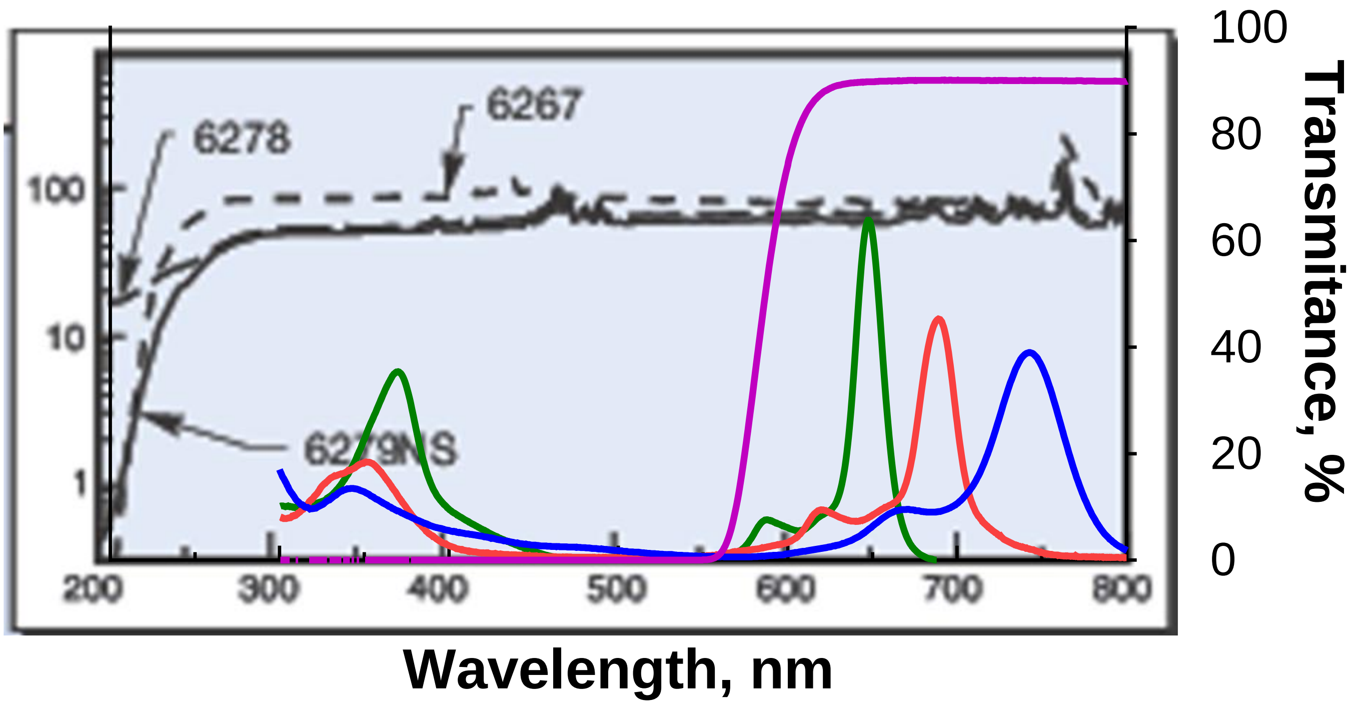

Fig. S129. Characteristics of the 450 W Xe – ozone free lamp (Newport, 6279NS) (black full line), together with transmittance of the Newport OG570 filter (magenta) and absorption spectra in water of three typical Pcs – **A5-Zn** with the lowest λ_{max} in the series (green), **C9-Zn** with the highest λ_{max} in the series (blue) and Si3-C with average λ_{max} in the series (red). The Y axis plots transmittance for filter, absorbance for dyes and irradiance for the lamp.

References

1 Michelsen, U., Kliesch, H., Schnurpfeil, G., Sobbi, A. K. & Wohrle, D. Unsymmetrically substituted benzonaphthoporphyrazines: A new class of cationic photosensitizers for the photodynamic therapy of cancer. *Photochemistry and Photobiology* **64**, 694–701 (1996). https://doi.org/10.1111/j.1751-1097.1996.tb03126.x

2 Redmond, R. W. & Gamlin, J. N. A compilation of singlet oxygen yields from biologically relevant molecules. *Photochemistry and Photobiology* **70**, 391–475 (1999). https://doi.org/10.1111/j.1751-1097.1999.tb08240.x

3 Zimcik, P. *et al.* Magnesium Azaphthalocyanines: An Emerging Family of Excellent Red-Emitting Fluorophores. *Inorganic Chemistry* **51**, 4215–4223 (2012). https://doi.org/10.1021/ic2027016
